# Computed tomography reveals multiple origins of extreme caudal vertebral pneumaticity in sauropod dinosaurs

**DOI:** 10.1111/joa.70177

**Published:** 2026-05-31

**Authors:** Samantha L. Beeston, Daniela Schwarz, Paul Upchurch, Stephen F. Poropat, Patrick Asbach, Philip D. Mannion

**Affiliations:** ^1^ Department of Earth Sciences University College London London UK; ^2^ Museum für Naturkunde, Leibniz Institute for Research on Evolution and Biodiversity Berlin Germany; ^3^ Western Australian Organic and Isotope Geochemistry Centre, School of Earth and Planetary Sciences Curtin University Bentley WA Australia; ^4^ Department of Radiology, Charité—Universitätsmedizin Berlin Corporate Member of Freie Universität Berlin and Humboldt‐Universität Zu Berlin Berlin Germany

**Keywords:** caudal vertebrae, computed tomography, evolution, postcranial skeletal pneumaticity, Sauropoda

## Abstract

Postcranial skeletal pneumaticity (PSP) is common in the presacral vertebrae of sauropod dinosaurs, but seemingly rare in their caudal vertebrae. Where identified, evidence for caudal vertebral PSP in sauropods is primarily based on the presence of external features, such as lateral fossae. However, such fossae can only be regarded as unequivocally pneumatic if communication between them and internal pneumatic bone texture can be confirmed. Based upon evidence from internal imaging, caudal vertebral PSP was previously known only in a rebbachisaurid diplodocoid (anterior caudal centrum and neural arch), some somphospondylan titanosauriforms (anterior caudal neural arches only) and saltasaurine titanosaurs (anterior–posterior caudal centra and neural arches). Here, we present novel CT scan data of caudal vertebrae of six Middle–Late Jurassic sauropods, representing several eusauropod lineages. We synthesise these new data with a comprehensive critical appraisal of purported external and internal evidence for caudal vertebral PSP in Sauropodomorpha. Newly sampled specimens of the non‐neosauropod eusauropods *Cetiosaurus* sp. (anterior caudal centrum), ‘*Cetiosaurus glymptonensis*’ (middle caudal centrum) and the mamenchisaurid *Wamweracaudia keranjei* (anterior–middle caudal vertebrae), as well as the dicraeosaurine diplodocoid *Dicraeosaurus sattleri* (anterior caudal vertebra), are apneumatic. By contrast, the anterior–posterior caudal centra and neural arches of the diplodocine diplodocoid *Tornieria africana* possess deeply invasive external fossae that communicate with internal pneumatic chambers. Shallow external fossae on the centra and neural arches of at least the anteriormost 24 caudal vertebrae of the brachiosaurid titanosauriform *Giraffatitan brancai* communicate with internal pneumatic chambers. We observe a repeated pattern of PSP invading the anterior caudal vertebrae, with at least five independent acquisitions and/or reversals within Neosauropoda. Furthermore, for the first time, we demonstrate that extreme caudal vertebral PSP, in which pneumaticity extends into the middle–posterior region of the tail, is not restricted to saltasaurines, with this having also evolved independently in diplodocines and brachiosaurids. Finally, we find that both small‐ and large‐bodied sauropods, including those with relatively short and long tails, evolved (and lost) caudal vertebral PSP. Therefore, the development of caudal vertebral PSP in sauropods does not appear to correspond with changes in body shape or mass. Instead, it might result from the opportunistic nature of pneumatic diverticula. However, given the high degree of inter‐ and intraspecific plasticity in its phylogenetic and serial distribution, we recognise that the evolution of PSP into the tail of sauropods might have been driven by a set of as‐yet unknown, complex selective pressures.

## Graphical abstract

Here, we present novel CT scan data of caudal vertebrae of six Middle–Late Jurassic sauropods, representing several eusauropod lineages. We synthesise these new data with a comprehensive critical appraisal of purported external and internal evidence for caudal vertebral PSP in Sauropodomorpha. We observe a repeated pattern of PSP invading the anterior caudal vertebrae, with at least five independent acquisitions and/or reversals within Neosauropoda.

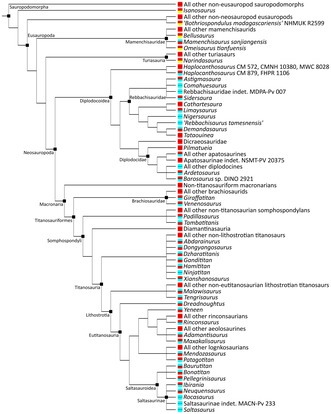

## 1 INTRODUCTION

Postcranial skeletal pneumaticity (PSP) occurs via pneumatic diverticula that extend from the lung and air sac systems, invading axial vertebral elements and replacing spongious bone texture with air (Maina, 2006; Moore & Schachner, 2025; Müller, 1908; O'Connor, 2006; O'Connor & Claessens, 2005; Schachner & Moore, 2025; Taylor & Wedel, 2021). These pneumatic diverticula adhere to external pneumatic fossae that communicate with internal pneumatic chambers (O'Connor, 2006). Today, PSP occurs only in Aves (O'Connor, 2006). However, PSP has been recognised in three extinct ornithodiran archosaur lineages, namely pterosauromorphs (Aureliano, Müller, et al., 2026; Buchmann et al., 2021; Butler et al., 2009), as well as non‐avialan theropod (Aranciaga Rolando et al., 2020; Aureliano, Almeida, et al., 2026; Benson et al., 2012; Britt, 1993; Gianechini & Zurriaguz, 2021; O'Connor & Claessens, 2005; Smith et al., 2021) and sauropodomorph dinosaurs (Aureliano et al., 2020, 2021, 2024; Beeston et al., 2026; Britt, 1993; Cerda et al., 2012; Schwarz et al., 2007; Schwarz & Fritsch, 2006; Wedel, 2003a, 2003b; Yates et al., 2012; Zurriaguz, 2025), for which avian‐like pneumatisation via air sac diverticula has been hypothesised. The development and distribution of PSP varies inter‐ and intra‐specifically in these ornithodiran clades, but the most commonly pneumatised elements are the presacral vertebrae (Benson et al., 2012; Britt, 1993; Moore & Schachner, 2025; O'Connor, 2004; Wedel, 2003a).

Based upon external morphology comprising lateral fossae and foramina, evidence for caudal vertebral PSP is seemingly rare in Pterosauromorpha (Buchmann et al., 2019) and Theropoda (Aranciaga Rolando et al., 2020; Benson et al., 2012; Kotevski et al., 2025), including extant Aves (Gutherz & O'Connor, 2022; O'Connor, 2004), although a lack of sampling means that the true distribution in theropods might be underestimated (see Gutherz & O'Connor, 2022). The condition in the caudal vertebrae of Sauropodomorpha is more variable, with several sauropod clades displaying evidence of PSP in the anterior, and, in rare instances, middle and posterior caudal vertebrae (Cerda et al., 2012; Wedel & Taylor, 2013). However, the evolutionary advantages and biomechanical implications of caudal vertebral PSP in sauropods remain uncertain (Wedel & Taylor, 2013).

Previous conclusions concerning the distribution of PSP in the caudal vertebrae of sauropods have been drawn primarily from observations of external indicators, such as lateral fossae on centra (e.g. Zurriaguz, 2025). Direct observation of communication between these external fossae and internal pneumatic chambers is rare, but is the only means by which we can confirm that these external indicators are evidence for unambiguous PSP (Beeston et al., 2026). Where such direct observations have been documented in sauropod vertebrae, via internal imaging such as CT or X‐ray scanning, or through natural breaks in bone, the results have provided otherwise inaccessible insights into the true distribution and extent of invasion of PSP into the caudal vertebrae of sauropods. The most notable instance of this pertains to the latest Cretaceous saltasaurine titanosaurs of South America, which possess the most extreme form of caudal vertebral PSP currently known among ornithodirans, extending from the anterior to posterior regions of the tail (Cerda et al., 2012; Zurriaguz & Cerda, 2017).

Here, we present new CT scan data of the caudal vertebrae of six species of sauropod dinosaurs. We combine these data with a comprehensive, critical review of the published literature of purported external and internal evidence for PSP in sauropodomorph caudal vertebrae. We utilise this new dataset to present a revised view on the distribution of caudal vertebral PSP in Sauropodomorpha, with implications for its evolution across the clade.

### 1.1 Institutional abbreviations

AMNH, American Museum of Natural History, New York City, New York, USA; AODF, Australian Age of Dinosaurs Fossil, Winton, Queensland, Australia; BAS, British Antarctic Survey, Cambridge, United Kingdom; BRSUG, University of Bristol Geology Department, Bristol, United Kingdom; BYU, Brigham Young University, Museum of Paleontology, Provo, Utah, USA; CH, Palaeontological collection, Department of Mineral Resources, Thailand; CM, Carnegie Museum of Natural History, Pittsburgh, Pennsylvania, USA; CMNH, Cleveland Museum of Natural History, Cleveland, Ohio, USA; CPPLIP, Centro de Pesquisas Paleontologicas ‘Llewelyn Ivor Price’, Universidade Federal do Triângulo Mineiro, Uberaba, Minas Gerais, Brazil; DINO, Dinosaur National Monument, Utah, USA; FHPR, Utah Field House of Natural History State Park Museum, Vernal, Utah, USA; FMNH, Field Museum of Natural History, Chicago, Illinois, USA; GMNH, Gunma Museum of Natural History, Gunma, Japan; LPP, Laboratório de Paleoecologia e Paleoicnologia, Universidade Federal de São Carlos, Brazil; MACN, Museo Argentino de Ciencias Naturales ‘Bernardino Rivadavia’, Buenos Aires, Argentina; MAL, Malawi Department of Antiquities Collection, Lilongwe and Nguludi, Malawi; MAU, Museo Municipal Argentino Urquiza, Rincón de los Sauces, Neuquén, Argentina; MB.R., Museum für Naturkunde, collection of fossil Reptilia, Berlin, Germany; MCS, Museo de Cinco Saltos, Río Negro, Argentina; MDPA, Museo del Desierto Patagónico de Añelo, Neuquén, Argentina; MfN, Museum für Naturkunde, Berlin, Germany; MLL, Museo Municipal de Las Lajas, Neuquén, Argentina; MNHAH, Museum of Nature and Human Activities, Hyogo, Japan; MNHN, Museum National d'Histoire Naturelle, Paris, France; MPCA, Museo Provincial ‘Carlos Ameghino’, Cipolletti, Río Negro, Argentina; MPMA, Museu de Paleontologia Prof. Antonio Celso de Arruda Campos, Monte Alto, Brazil; MSNM, Museo di Storia Naturale di Milano, Milan, Italy; MUPA, Museo de Paleontología de Castilla‐La Mancha, Cuenca, Spain; MWC, Museum of Western Colorado, Colorado, USA; NAMAL, Mountain America Museum of Ancient Life, Lehi, Utah, USA; NHMUK, Natural History Museum, London, United Kingdom; NSMT, National Museum of Nature and Science, Tokyo, Japan; OUMNH, Oxford University Museum of Natural History, Oxford, United Kingdom; QMF, Queensland Museum Fossil, Brisbane, Australia; SMA, Sauriermuseum Aathal, Aathal, Switzerland; ULBRA, Museu de Ciências Naturais, Universidade Luterana do Brasil, Canoas, Rio Grande do Sul, Brazil; UNPSJB, Universidad Nacional de la Patagonia San Juan Bosco, Comodoro Rivadavia, Argentina; YM‐INPC, Yamana locality collection, Instituto Nacional de Patrimonio Cultural, Ecuador; YPM, Yale Peabody Museum, New Haven, Connecticut, USA.

### 1.2 Anatomical abbreviations

ACDL‐F, anterior centrodiapophyseal lamina fossa; Ca, caudal vertebra; CDF, centrodiapophyseal fossa; CPRF, centroprezygapophyseal fossa; ch, chevron; LF, lateral foramen/ fossa; LPF, lateral pneumatic foramen/ fossa; na, neural arch; nc, neural canal; ns, neural spine; PACPRF, parapophyseal centroprezygapophyseal fossa; POCDF, postzygapophyseal centrodiapophyseal fossa; POSDF, postzygapophyseal spinodiapophyseal fossa; poz, postzygapophysis; PRCDF, prezygapophyseal centrodiapophyseal fossa; prz, prezygapophysis; PSP, postcranial skeletal pneumaticity; SDF, spinodiapophyseal fossa; SPOF, spinopostzygapophyseal fossa; SPOL‐F, spinopostzygapophyseal lamina fossa; SPRF, spinoprezygapophyseal fossa; SPRL‐F, spinoprezygapophyseal lamina fossa; tp, transverse process; vf, ventral fossa.

## 2 MATERIALS AND METHODS

### 2.1 Terminology

When describing evidence for the pneumaticity of a caudal vertebra, we separate the element into two regions: the centrum and the neural arch, which includes the neural spine. The terminology used to describe the vertebral laminae and fossae follows Wilson (1999) and Wilson et al. (2011), respectively. The pneumatic terminology used herein is defined in Table 1. We utilise the pneumaticity criterion outlined in Beeston et al. (2026), in which an element is regarded as pneumatic only if it possesses direct communication between external fossae and internal chambers and/or if these external fossae are subdivided (O'Connor, 2006; Wedel, 2007). We define ‘extreme’ caudal vertebral pneumaticity as occurring when middle and/or posterior caudal vertebrae are pneumatised, in addition to the anterior caudal vertebrae.

**TABLE 1:** Summary of pneumatic terms used herein.

Term	Definition
Camellate	Complex internal pneumatic structure entirely composed of camellae; neural arch laminae not reduced; large external fossae may also be present; small internal pneumatic chambers that range in size from 2 to 20 mm in diameter, and are separated by very thin bone walls; walls are generally angular, with no identifiable branching pattern, and range in thickness from 3 mm to less than 1 mm; numerous small cavities and angular walls produce a honeycombed pattern (Britt, 1993, 1997; Wedel et al., 2000; Wedel, 2003b)
Camerate	Simple internal pneumatic structure with a small number of relatively large, enclosed camerate chambers with regular branching pattern; camerae separated by relatively thick bone walls ranging in thickness from 2 to 10 mm; pneumatic chambers enclosed by ostial margins constituting a foramen; rounded and smoothly contoured chambers ranging in size from 5 mm to more than 150 mm; recognisable branching patterns with interconnecting pneumatic foramina and usually communicate with the lateral foramina; cameral generations usually limited to 3 (Britt, 1993, 1997; Wedel et al., 2000; Wedel, 2003b)
Foramen	Anatomical passage; a hole or opening passing through cortical bone (Britt, 1993; O'Connor, 2006)
Fossa	Surficial depression; a concavity positioned in an anatomical surface lacking a distinct rim of cortical bone (Britt, 1993; O'Connor, 2006)
Pneumatic fossa	Excavations that are broad in contour and are not enclosed by osteal margins to form a foramen (Wedel, 2003b)
Postcranial skeletal pneumaticity (PSP)	The modification of the postcranial skeleton by pneumatic diverticula of the respiratory system (Wedel & Taylor, 2013)
Protocamerate	Pneumatic tissue with properties of both camellae and camerae: structures which are not large enough to be considered camerae, but also present a camellate array internally (Aureliano et al., 2024)
Spongiosa/ Cancellous	Compact, apneumatic, spongy or cancellous bone wherein the volume of pore space is higher than the volume of bone tissue. Depending on the degree of porosity, spongiosa is subdivided into fine cancellous bone, coarse cancellous bone and trabecular bone, from lower to higher porosity (Francillon‐Vieillot et al., 1989; Wedel, 2005)
Trabecular bone	A type of cancellous bone that provides lightweight internal support wherein rod‐shaped bone tissue (=trabeculae) shows a precise three‐dimensional spatial arrangement that reflects mechanical forces acting on the bone (Francillon‐Vieillot et al., 1989; Woodruff et al., 2017)
True lateral pneumatic foramen (pleurocoel)	Depressions in the lateral walls of vertebrae with at least partially sharply‐outlined borders that cut deeply into the outer wall of the vertebra (Janensch, 1947)

### 2.2 New CT scans of sauropodomorph dinosaur caudal vertebrae

#### 2.2.1 Eusauropoda: *Cetiosaurus* sp.

*Cetiosaurus oxoniensis* is known from the Middle Jurassic of the UK and has a complicated taxonomic history (Upchurch & Martin, 2002, 2003). In most analyses, it is recovered as an early‐diverging non‐neosauropod eusauropod (e.g. Gomez et al., 2021). The lectotype comes from the Bathonian Forest Marble Formation and is accessioned at the OUMNH (Phillips, 1871; Upchurch & Martin, 2003). An anterior caudal centrum preserving its neural arch bases (OUMNH PAL‐J.50308; Table 2) from the same geographical area and approximate stratigraphic horizon lacks specific provenance information, but is accessioned as *Cetiosaurus* sp. Although we cannot be certain that it is referable to *Cetiosaurus*, we consider it representative of at least a *Cetiosaurus*‐like taxon.

**TABLE 2:** Summary of sauropodomorph caudal vertebrae examined herein and elements newly CT scanned.

Taxon	Specimen no.	Locality no.	Caudal no.	Elements CT scanned
Eusauropoda *Cetiosaurus* sp.	OUMNH PAL‐J.50308	—	Anterior	Anterior, OUMNH PAL‐J.50308
Eusauropoda *Cetiosaurus glymptonensis*	OUMNH PAL‐J.13750–J13758	—	Middle–posterior	Middle, OUMNH PAL‐J.13751
Mamenchisauridae *Wamweracaudia keranjei*	MB.R.2091.1–30	G	Anterior–posterior	Anterior, MB.R.2091.27; middle, MB.R.2091.16, MB.R.2091.19
Dicraeosaurinae *Dicraeosaurus sattleri*	MB.R.3681–3687	M	Ca1–Ca7	Ca3, MB.R.3683
Diplodocinae *Tornieria africana*	MB.R.2956.1–24, MB.R.2957–2958, MB.R.6082	dd	Anterior: MB.R.2956.1, MB.R.6082; middle: MB.R.2956.2–10, MB.R.2956.15, MB.R.2957–2958; posterior: MB.R.2956.11–14, MB.R.2956.16–24	MB.R.2956.1, MB.R.2956.4, MB.R.2956.5, MB.R.2956.6, MB.R.2956.12, MB.R.2956.14, MB.R.2956.19
Brachiosauridae *Giraffatitan brancai*	MB.R.5000	no	Ca2–Ca51	Ca2, MB.R.5000.1; Ca7, MB.R.5000.6; Ca15, MB.R.5000.14; Ca19, MB.R.5000.18; Ca24, MB.R.5000.23
	MB.R.2921	Aa	Ca1–Ca18	
	MB.R.3736	D	Ca1–Ca31	

#### 2.2.2 Eusauropoda: ‘*Cetiosaurus glymptonensis*’

Originally described as a species of *Cetiosaurus* (Phillips, 1871; Upchurch & Martin, 2003), ‘*Cetiosaurus glymptonensis*’ is represented by nine middle–posterior caudal vertebrae (OUMNH PAL‐J.13750–PAL‐J.13758; Table 2) from the Bathonian Forest Marble Formation of the UK. Upchurch and Martin (2003) suggested that it might represent an early diplodocoid, but others have suggested that it is best regarded as an indeterminate non‐neosauropod eusauropod that differs from *Cetiosaurus* (Mannion & Moore, 2025; Whitlock, 2011), which we follow here.

#### 2.2.3 Mamenchisauridae: *Wamweracaudia keranjei*

The holotypic caudal vertebral series of *Wamweracaudia keranjei* derives from locality ‘G’ of the Tithonian (Upper Jurassic) Upper Dinosaur Member of the Tendaguru Formation, Tanzania (Janensch, 1929; Mannion et al., 2019). *Wamweracaudia* has been consistently recovered as a non‐neosauropod eusauropod, nested within Mamenchisauridae (e.g. Mannion et al., 2019). The holotype is a series of 30 articulated anterior–posterior caudal vertebrae (MfN MB.R.2091.1–30; Table 2), with the anteriormost preserved element (MB.R.2091.30) representing one of the most proximal caudal vertebrae.

#### 2.2.4 Dicraeosaurinae: *Dicraeosaurus sattleri*

The holotype of *Dicraeosaurus sattleri* is a partial sauropod skeleton from locality ‘M’ of the Tithonian Upper Dinosaur Member of the Tendaguru Formation (Janensch, 1929). *Dicraeosaurus* is consistently positioned as a dicraeosaurine dicraeosaurid within Diplodocoidea in phylogenetic analyses (e.g. Mannion & Moore, 2025; Whitlock, 2011). The holotypic material of *Dicraeosaurus sattleri* analysed herein comprises seven anterior caudal vertebrae (Ca1, MB.R.3681; Ca2, MB.R.3682; Ca3, MB.R.3683; Ca4, MB.R.3684; Ca5, MB.R.3685; Ca6, MB.R.3686; Ca7, MB.R.3687; Janensch, 1929: plate VII; Table 2).

#### 2.2.5 Diplodocinae: *Tornieria africana*

Anterior–posterior caudal vertebrae (MB.R.2956.1–24, 2957–2958, 6082; Table 2) from locality ‘dd’ of the upper Kimmeridgian Middle Dinosaur Member of the Tendaguru Formation were referred to *Tornieria africana* by Remes (2006). Those authors described the caudal vertebrae as discontinuous and belonging to more than one individual. Herein, we follow the serial position of each element as assigned by Remes (2006). *Tornieria* is recovered as a diplodocine diplodocid within Diplodocoidea in phylogenetic analyses (e.g. Mannion et al., 2019; Tschopp et al., 2015).

#### 2.2.6 Brachiosauridae: *Giraffatitan brancai*

Janensch (1929) referred three caudal vertebral series to *Giraffatitan brancai* (Table 2). Each caudal vertebral series derives from a different locality within the Tendaguru Formation: locality ‘no’ from the Tithonian Upper Dinosaur Member comprising Ca2–Ca51 (MB.R.5000; part of the mounted skeleton on display at the MfN); locality ‘Aa’ from the Kimmeridgian Middle Dinosaur Member comprising Ca1–Ca18 (MB.R.2921); and locality ‘D’ from the Tithonian Upper Dinosaur Member comprising Ca1–Ca31 (MB.R.3736). *Giraffatitan* is consistently recovered within the titanosauriform clade Brachiosauridae in phylogenetic analyses (e.g. Carballido & Sander, 2014; Mannion et al., 2017).

### 2.3 Previously CT scanned sauropodomorph dinosaur caudal vertebrae

We attempted to obtain internal imaging data of all previously published scanned sauropodomorph caudal vertebrae to include in this contribution (Table 3). However, in some instances, raw data had been lost and were inaccessible (Saegusa & Ikeda, 2014; Wedel, 2009; Wedel et al., 2021; Zurriaguz et al., 2017; Zurriaguz & Cerda, 2017), or were not made available (Mocho, Perez‐Garcia, et al., 2019). Data from these instances have not been included in the figures presented herein.

**TABLE 3:** Summary of previously published CT scanned sauropodomorph caudal vertebrae.

Taxon	Specimen no.	Caudal position	References	Notes
Sauropodomorpha *Pampadromaeus barberenai*	ULBRA‐PVT016	Two anterior	Aureliano et al. (2022)	
Sauropodomorpha *Thecodontosaurus antiquus*	BRSUG Cb 4164, 4714	Six anterior–middle	Beeston et al. (2026)	
Diplodocoidea *Haplocanthosaurus* sp.	MWC 8028	Ca3	Wedel et al. (2021)	Not included herein
Rebbachisauridae indet.	MDPA‐Pv 007	Ca1 or Ca2	Windholz et al. (2024)	
Diplodocidae *Pilmatueia faundezi*	MLL‐Pv‐016	Middle	Windholz et al. (2020)	
Diplodocinae *Barosaurus* sp. (immature individual)	DINO 2921	Anterior	Melstrom et al. (2016)	
Diplodocinae *Barosaurus* sp.	NAMAL‐106	Terminal two	van der Linden et al. (2026)	
Euhelopodidae *Tambatitanis amicitiae*	MNHAH D‐1029280	Ca1–Ca6	Saegusa and Ikeda (2014)	Not included herein
Diamantinasauria *Wintonotitan wattsi*	QMF7292	Anterior–posterior	Hocknull et al. (2021)	
Titanosauria. indet.	MSNM V7157	Anterior	Dal Sasso et al. (2016)	
Lithostrotia indet.	MAU‐Pv‐LI‐601	Anterior	Cruzado‐Caballero et al. (2023)	
Lithostrotia indet.	MAU‐Pv‐LJ‐472/1	Middle	Cruzado‐Caballero et al. (2023)	
Lithostrotia indet.	MUPA ALG 143, 144	Anterior–middle	Mocho, Perez‐Garcia, et al. (2019)	Not included herein
Lithostrotia *Malawisaurus dixeyi*	MAL‐200	Anterior	Wedel (2009)	Not included herein
Eutitanosauria indet.	BAS D.8621.25	Anterior	Barrett et al. (2026)	
Saltasaurinae indet.	MACN‐Pv 233–5	Posterior	Zurriaguz et al. (2017)	Not included herein
Saltasaurinae *Ibirania parva*	MPMA 08–0060‐07	Middle	Navarro et al. (2022)	
Saltasaurinae *Neuquensaurus australis*	MCS‐Pv 5/1, 5/8	Anterior, middle	Zurriaguz and Cerda (2017)	MCS‐Pv 5/1 not included herein
Saltasaurinae *Rocasaurus muniozi*	MPCA‐Pv 47, 57, 58	Anterior–middle	Zurriaguz and Cerda (2017)	
Saltasaurinae *Yamanasaurus lojaensis*	YM‐INPC‐014	Middle	Apesteguía et al. (2020)	

### 2.4 CT scanning protocol

The anterior caudal centrum of *Cetiosaurus* sp. (OUMNH PAL‐J.50308) and one middle caudal centrum of ‘*Cetiosaurus glymptonensis*’ (OUMNH PAL‐J.13751) were scanned on a Nikon XT H 225 ST 2x micro‐CT scanner at the University of Birmingham, UK, in March, 2026, with a tube voltage of 185‐kV for OUMNH PAL‐J.50308 and 200‐kV for OUMNH PAL‐J.13751, and a voxel size of 0.09 mm for OUMNH PAL‐J.50308 and 0.07 mm for OUMNH PAL‐J.13751. Three anterior–middle caudal vertebrae of *Wamweracaudia* (anterior, MB.R.2091.27; middle, MB.R.2091.16; middle, MB.R.2091.19), one anterior caudal vertebra of *Dicraeosaurus sattleri* (Ca3, MB.R.3683), seven anterior–posterior caudal vertebrae of *Tornieria africana* (anterior, MB.R.2956.1; middle, MB.R.2956.4; middle, MB.R.2956.5; middle, MB.R.2956.6; posterior, MB.R.2956.12; posterior, MB.R.2956.14; posterior, MB.R.2956.19) and five anterior–middle caudal vertebrae of *Giraffatitan brancai* (Ca2, MB.R.5000.1; Ca7, MB.R.5000.6; Ca15, MB.R.5000.14; Ca19, MB.R.5000.18; Ca24, MB.R.5000.23) were CT scanned at the Department of Radiology at the Charité in Berlin, Germany, in November 2024, February 2025 and October 2025, using a 320‐section multidetector CT unit (Aquilion Prime; Canon Medical Systems, Otawara, Japan). A helical scan mode with a rotation time of 1.0 s was chosen. The tube voltage was set to 135‐kV and the voxel size varied between 0.2 and 0.7 mm.

CT scans were analysed, and screenshots of CT slices taken, in Avizo 9.7 (FEI Visualization Science Group; https://www.thermofisher.com) and 3D Slicer 5.8.0 (https://www.slicer.org/). Figures were assembled in Inkscape 1.4 (https://inkscape.org/). All newly presented CT scan data are available via MorphoSource (Project ID: 000857117; https://www.morphosource.org/projects/000857117; Supplemental Data).

## 3 RESULTS

### 3.1 Assessment of postcranial skeletal pneumaticity in newly CT scanned sauropodomorph dinosaur caudal vertebrae

#### 3.1.1 *Cetiosaurus* sp.

**FIGURE 1:**
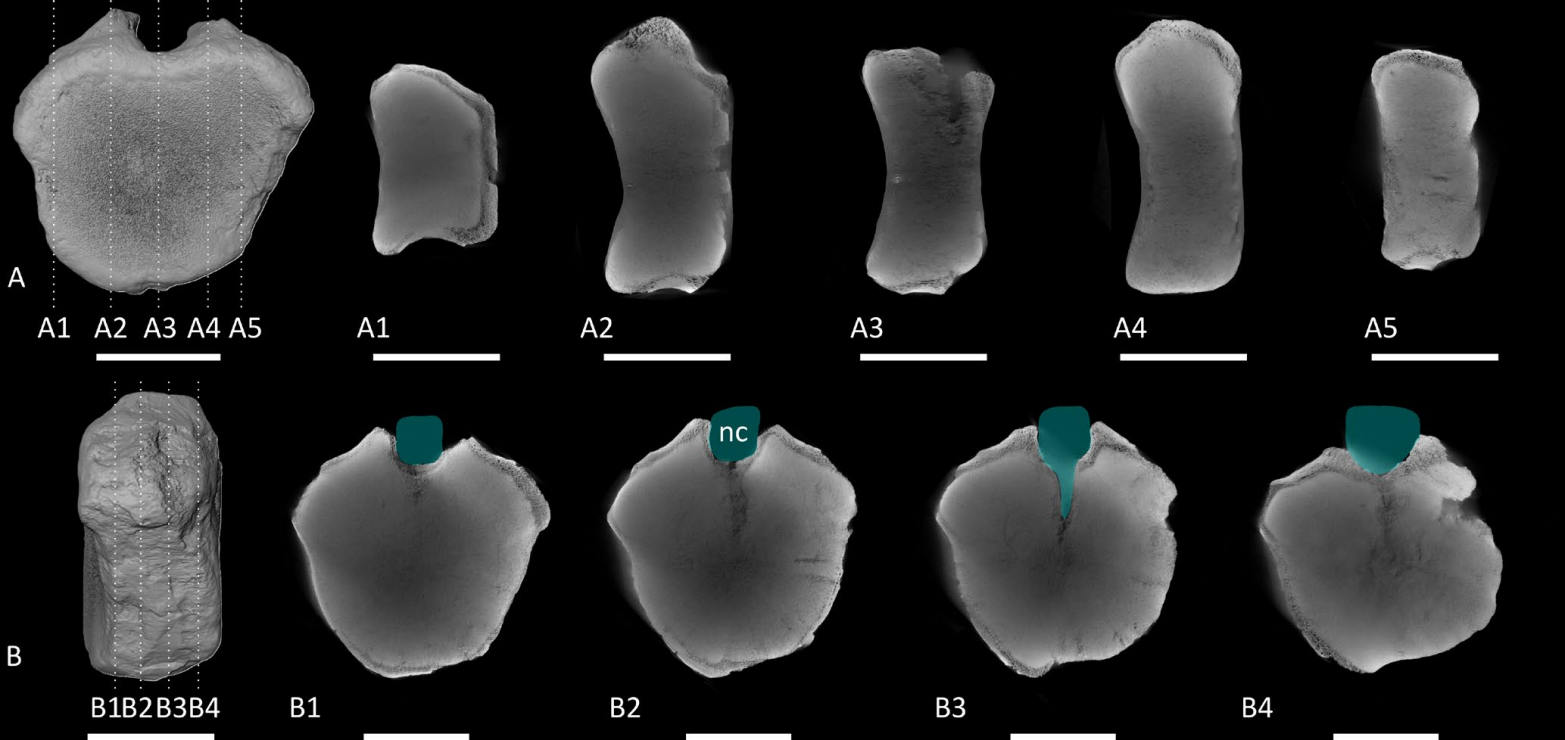
CT scan of *Cetiosaurus* sp. anterior caudal vertebra OUMNH PAL‐J.50308. (A, B) 3D rendering (A) anterior view, (B) left lateral view. (A1–A5) CT scan slices in parasagittal cross‐section. (B1–B4) CT scan slices in transverse cross‐section. nc, neural canal. Key: blue highlight. Scale bars = 100 mm.

The lateral surface of the anterior caudal centrum (OUMNH PAL‐J.50308) is shallowly concave anteroposteriorly. Eroded surfaces around the edges of the centrum and the broken neural arch bases reveal a dense, spongious internal texture and the apneumatic nature of the element is confirmed from CT scans (Figure 1). Vascularisation increases around the edges of the element and the neural canal (Figure 1). Several nutrient foramina pierce the lateral and ventral surfaces of the centrum and the floor of the neural canal; CT scans reveal that some of these foramina extend a considerable distance inside the apneumatic centra (e.g. see Figure 1B2,B3).

#### 3.1.2 ‘*Cetiosaurus glymptonensis*’

**FIGURE 2:**
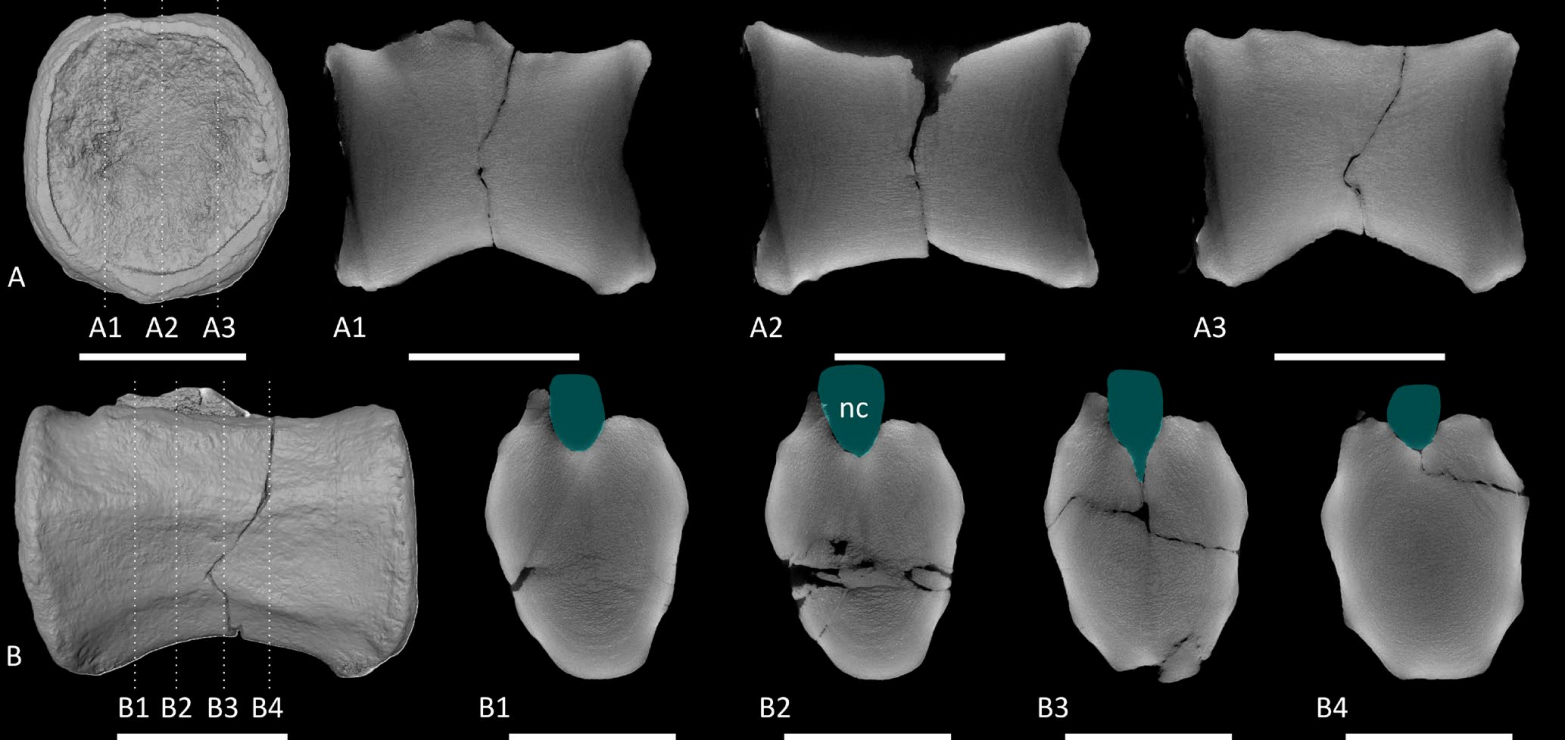
CT scan of ‘*Cetiosaurus glymptonensis*’ middle caudal vertebra OUMNH PAL‐J.13751. (A–B) 3D rendering (A) anterior view, (B) left lateral view. (A1–A3) CT scan slices in parasagittal cross‐section. (B1–B4) CT scan slices in transverse cross‐section. nc, neural canal. Key: blue highlight. Scale bars = 100 mm.

The middle–posterior caudal centra of ‘*Cetiosaurus glymptonensis*’ (OUMNH PAL‐J.13750–PAL‐J.13758) are shallowly anteroposteriorly concave and lack external evidence for PSP. Broken and eroded surfaces of the centra and neural arch bases reveal a compact, apneumatic spongious internal texture. CT scans of a middle caudal centrum that preserves its neural arch bases (OUMNH PAL‐J.13751) confirm this apneumaticity (Figure 2). Vascularisation increases minimally in the centre of the element (Figure 2A2,B1–B3). Nutrient foramina on the lateral surfaces of OUMNH PAL‐J.13751 extend a considerable distance inside the centrum (e.g. see Figure 2B1,B2).

#### 3.1.3 Wamweracaudia keranjei

**FIGURE 3:**
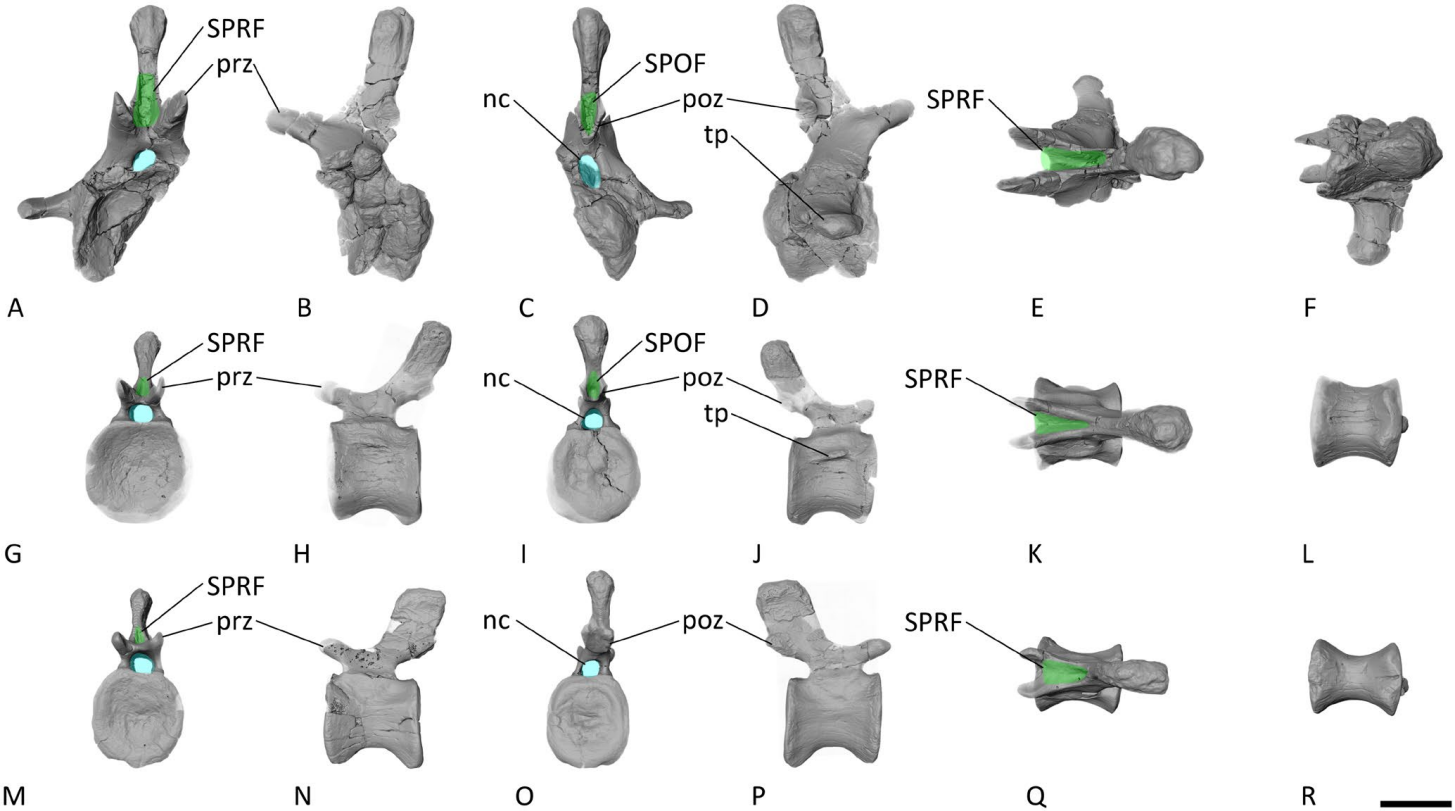
3D renderings of CT scanned *Wamweracaudia keranjei* caudal vertebrae MB.R.2091. (A–F) anterior caudal vertebra MB.R. 2091.27, (G–L) middle caudal vertebra MB.R. 2091.19, (M–R) middle caudal vertebra MB.R. 2091.16; (A, G, M) anterior view, (B, H, N) left lateral view, (C, I, O) posterior view, (D, J, P) right lateral view, (E, K, Q) dorsal view, (F, L, R) ventral view. nc, neural canal; poz, postzygapophysis; prz, prezygapophysis; SPOF, spinopostzygapophyseal fossa; SPRF, spinoprezygapophyseal fossa; tp, transverse process. Key: Blue highlight, neural canal; green highlight, neural spine fossa. Scale bar = 100 mm.

Each centrum of MB.R.2091.20–23 possesses a shallow lateral fossa on the left and right surfaces, immediately ventral to the transverse processes. These fossae are not true lateral pneumatic fossae/foramina (LPF) as they lack defined rims, and they are absent in all other elements (Figure 3). The anterior–middle caudal vertebrae each possess a spinoprezygapophyseal fossa (SPRF) and a spinopostzygapophyseal fossa (SPOF). However, these fossae have been artificially reconstructed in some elements, making their precise distribution unknown.

**FIGURE 4:**
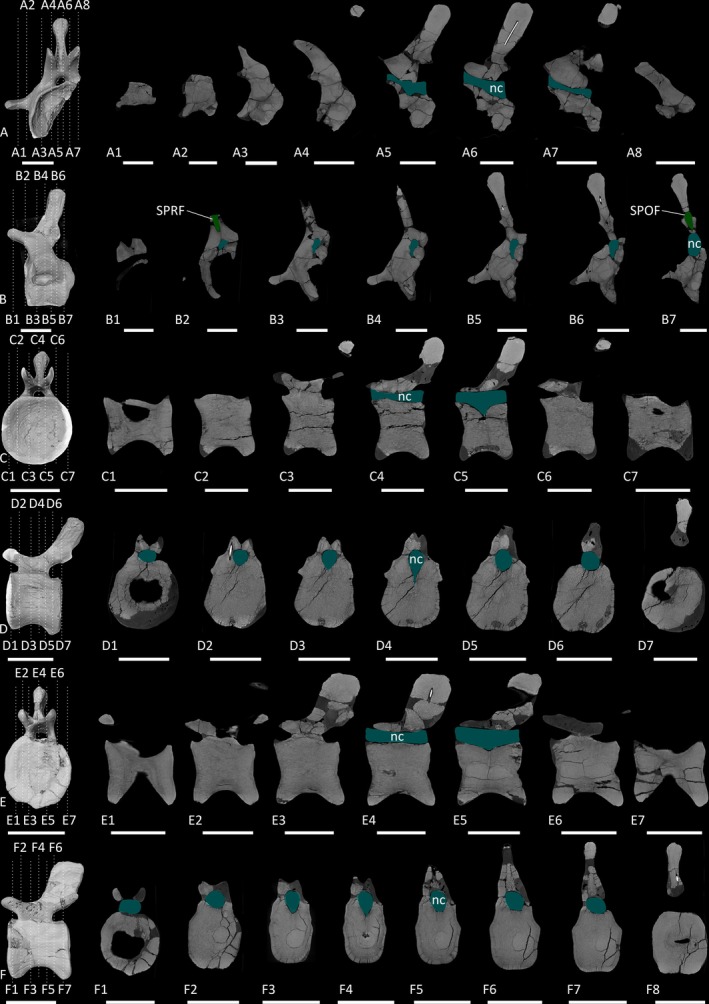
CT scan of *Wamweracaudia keranjei* anterior and middle caudal vertebrae. (A–F) 3D rendering (A, C, E) anterior view, (B, D, F) left lateral view; (A, B) anterior caudal vertebra MB.R. 2091.27, (C, D), middle caudal vertebra MB.R. 2091.19, (E, F) middle caudal vertebra MB.R. 2091.16; (A1–A8, C1–C7, E1–E7) CT scan slices in parasagittal cross‐section, (B1–B7, D1–D7, F1–F8) CT scan slices in transverse cross‐section. nc, neural canal; SPOF, spinopostzygapophyseal fossa; SPRF, spinoprezygapophyseal fossa. Key: Blue highlight, neural canal; green highlight, neural spine fossa. Scale bars = 100 mm.

Nutrient foramina are present on the lateral and/or ventrolateral surfaces of the centra of MB.R.2091.14, MB.R.2091.16, MB.R.2091.19, MB.R.2091.20 and MB.R.2091.27. CT scans reveal that some of these nutrient foramina pierce the cortical bone, whereas others do not. The CT scans of anterior (MB.R.2091.27) and middle (MB.R.2021.19, MB.R.2021.16) caudal vertebrae reveal a dense, apneumatic spongious internal texture in the centra and neural arches, as interpreted by Mannion et al. (2019) from external observations and broken surfaces (Figure 4). The prezygapophyses, transverse process and neural spine of MB.R.2091.27 are less vascularised than the centrum and neural arch bases (Figure 4A1–A8,B1–B7).

#### 3.1.4 Dicraeosaurus sattleri

**FIGURE 5:**
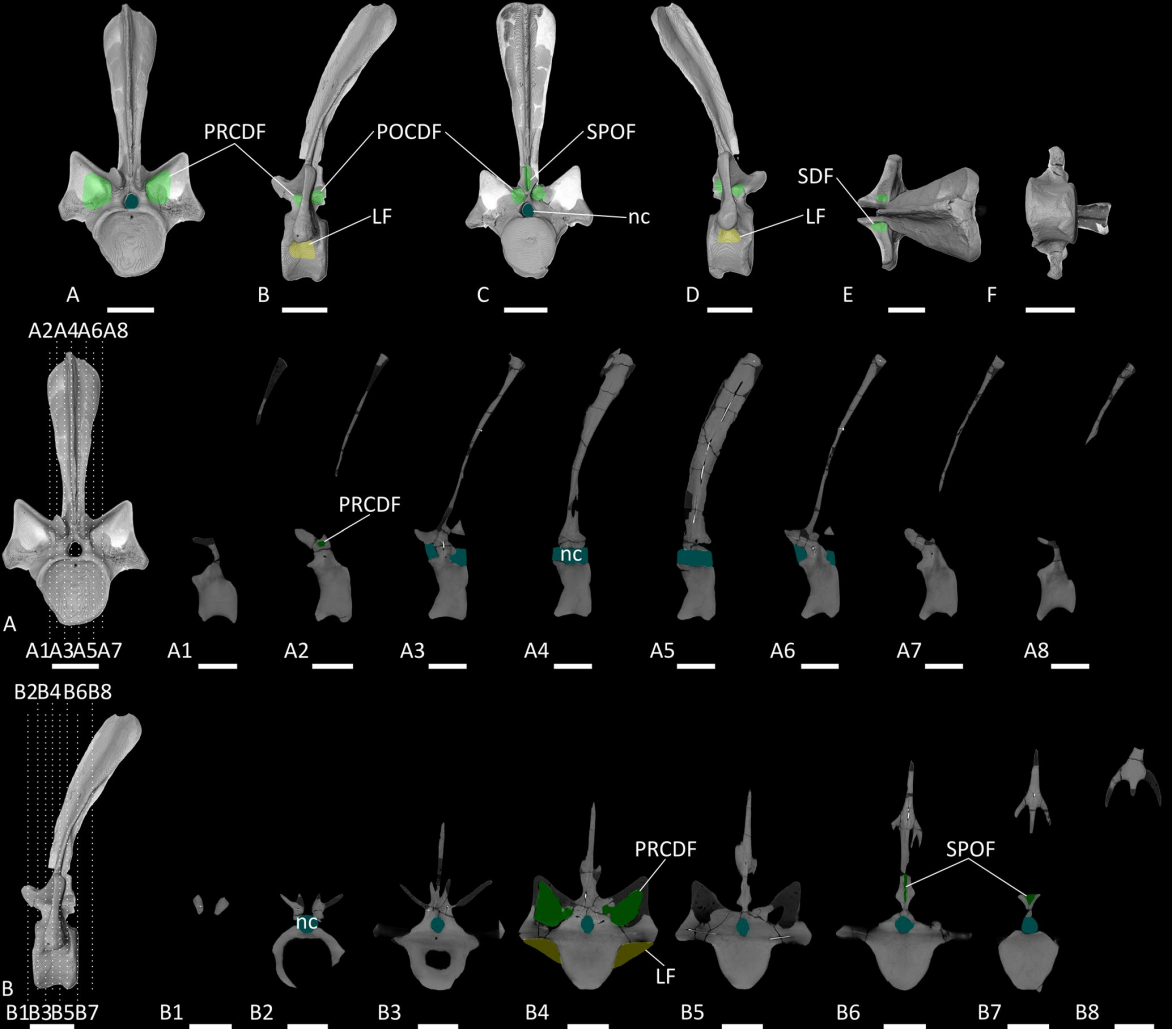
CT scan of *Dicraeosaurus sattleri* caudal vertebra 3 MB.R.3683. (A–F) 3D rendering (A) anterior view, (B) left lateral view, (C) posterior view, (D) right lateral view, (E) dorsal view, (F) ventral view; (A1–A8) CT scan slices in parasagittal cross‐section, (B1–B8) CT scan slices in transverse cross‐section. LF, lateral fossa; nc, neural canal; POCDF, postzygapophyseal centrodiapophyseal fossa; PRCDF, prezygapophyseal centrodiapophyseal fossa; SDF, spinodiapophyseal fossa; SPOF, spinopostzygapophyseal fossa. Key: Blue highlight, neural canal; green highlight, neural spine fossa; yellow highlight, centrum fossa. Scale bars = 100 mm.

The seven anterior caudal vertebrae of *Dicraeosaurus*, evaluated herein, are generally well preserved. However, some elements are reconstructed with plaster, creating artificial fossae and laminae that reflect true fossae and laminae present in other elements (see Janensch, 1929: plate VII). Ca2–Ca4 each possess a shallow lateral fossa that is not a true LPF, whereas the lateral surfaces of the centra of Ca1 and Ca5–Ca7 are flat. The ventral surface of Ca3 is flat to shallowly convex transversely (Figure 5), whereas the ventral surfaces of Ca4–Ca7 are transversely concave and laterally bounded by parallel anteroposterior ridges. Unfortunately, the ventral surfaces of Ca1–Ca2 are inaccessible. The anterior surfaces of the transverse processes of all elements are concave. Ca3 possesses a prezygapophyseal centrodiapophyseal fossa (PRCDF) that is deep on the right side and shallow on the left side. The right PRCDF excavates the ventrolateral surface of the corresponding prezygapophysis. The presence or absence of a PRCDF in Ca1–Ca2 cannot be determined due to reconstruction, and Ca4–Ca7 lack a PRCDF. Ca3–Ca4 possess a postzygapophyseal centrodiapophyseal fossa (POCDF) ventrolateral to each postzygapophysis, whereas Ca5–Ca6 lack a POCDF; reconstruction in the corresponding area of Ca1–Ca2 and Ca7 inhibits assessment of the presence or absence of this fossa.

All elements lack a SPRF and Ca2–Ca6 each possess a SPOF. The corresponding areas of Ca1 and Ca7 are incomplete and reconstructed, respectively. As such, the presence or absence of a SPOF cannot be determined in these elements. A spinodiapophyseal fossa (SDF) occurs in all elements except for Ca1, potentially owing to incomplete preservation of this area in the latter. Eroded surfaces on the prezygapophyses, transverse process tips and the dorsal surface of the neural spines reveal a spongious internal texture. CT scans of Ca3 confirm this, with the centrum and neural arch possessing a dense, apneumatic spongious internal texture (Figure 5). Vascularisation increases in the dorsal half of the centrum and the neural arch bases (Figure 5).

#### 3.1.5 Tornieria africana

**FIGURE 6:**
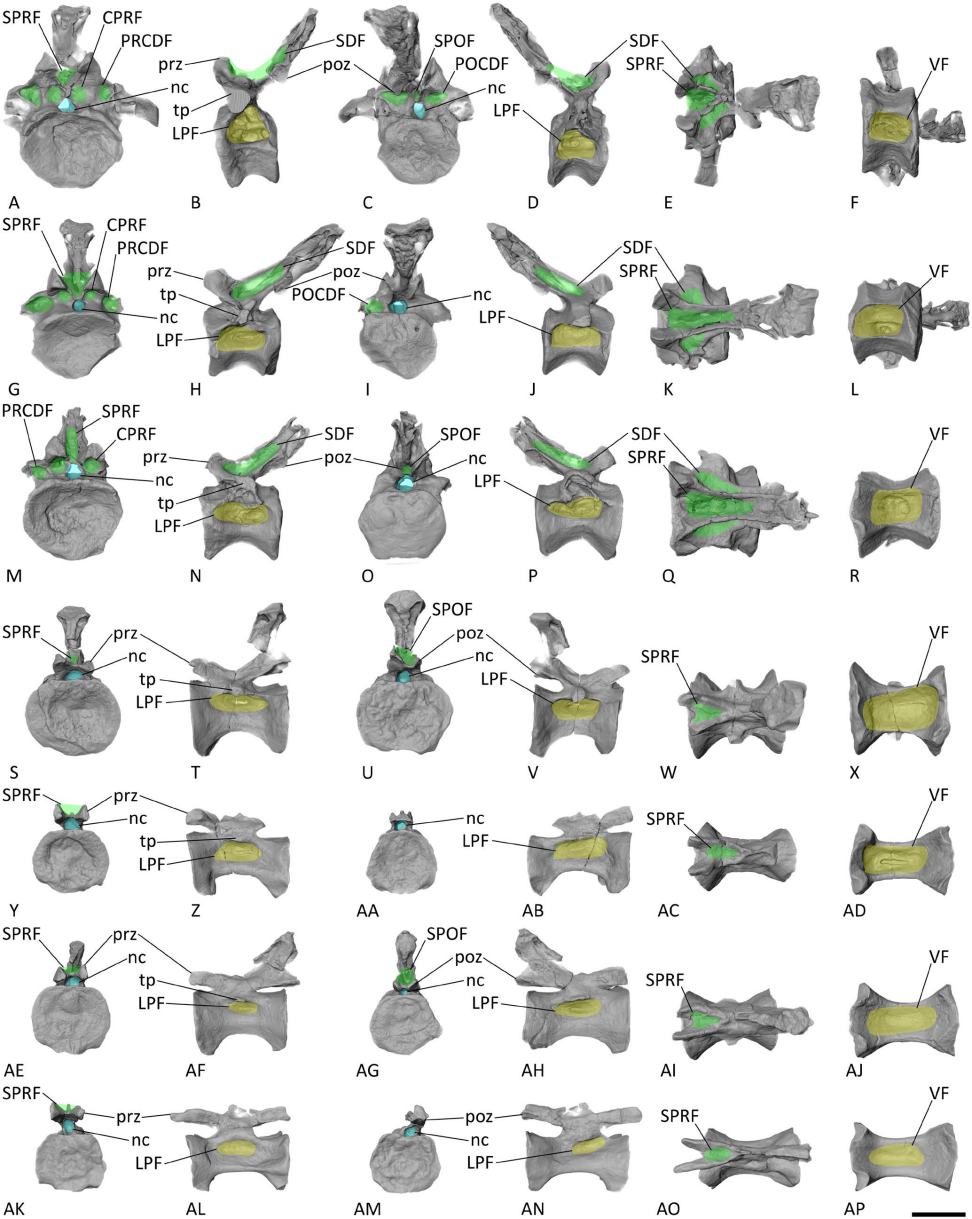
3D rendering of CT scanned *Tornieria africana* caudal vertebrae MB.R. 2956. (A–F) anterior caudal vertebra MB.R.2956.1, (G–L) middle caudal vertebra MB.R. 2956.4, (M–R) middle caudal vertebra MB.R. 2956.5, (S–X) middle caudal vertebra MB.R. 2956.6, (Y–AD) posterior caudal vertebra MB.R.2956.12, (AE–AJ) posterior caudal vertebra MB.R.2956.14, (AK–AP) posterior caudal vertebra MB.R.2956.19; (A, G, M, S, Y, AE, AK) anterior view, (B, H, N, T, Z, AF, AL) left lateral view, (C, I, O, U, AA, AG, AM) posterior view, (D, J, P, V, AB, AH, AN) right lateral view, (E, K, Q, W, AC, AI, AO) dorsal view, (F, L, R, X, AD, AJ, AP) ventral view. CPRF, centroprezygapophyseal fossa; LF, lateral fossa; nc, neural canal; POCDF, postzygapophyseal centrodiapophyseal fossa; poz, postzygapophysis; PRCDF, prezygapophyseal centrodiapophyseal fossa; prz, prezygapophysis; SDF, spinodiapophyseal fossa; SPOF, spinopostzygapophyseal fossa; SPRF, spinoprezygapophyseal fossa; tp, transverse process; VF, ventral fossa. Key: Blue highlight, neural canal; green highlight, neural spine fossa; yellow highlight, centrum fossa. Scale bar = 100 mm.

Based on comparisons with more complete caudal vertebral series in other diplodocines (e.g. Hatcher, 1901), among the elements CT scanned, MB.R.2956.1 is interpreted as an anterior caudal vertebra, MB.R.2956.4 and MB.R.2956.5 are proximal‐middle elements, and MB.R.2956.6 is a distal‐middle element. MB.R.2956.12 and MB.R.2956.14 are regarded as proximal‐posterior caudal vertebrae owing to the reduction of transverse processes to prong‐like ridges, and a well‐developed but dorsoventrally short neural spine in MB.R.2956.14 (note that the neural spine of MB.R.2956.12 is broken; Figure 6). MB.R.2956.19 is regarded as a distal‐posterior element based upon the absence of transverse processes and a highly reduced neural spine that is barely distinguishable from the zygapophyseal table.

Except for MB.R.6053 (Ca1), all anterior caudal centra possess a deep LPF that ramifies into several subfossae. In MB.R.6053, the lateral surface is flat to shallowly concave, lacking a LPF. Given this, *Tornieria* might have possessed a pneumatic hiatus (sensu Wedel & Taylor, 2013) between the sacral and caudal centra. However, this is speculative, since no sacral vertebrae are currently known for *Tornieria*, and MB.R.6053 was not CT scanned. In the middle caudal centra, the LPF is undivided and shallower than in the preceding elements, and it is further reduced to a shallow fossa in the posterior caudal centra. Ventral to the right LPF in MB.R.2956.1, the lateral surface possesses four nutrient foramina that are positioned centrally and extend in a dorsoventral line (Figure 6D). However, the lateral surface ventral to the left LPF lacks nutrient foramina. Except for MB.R.6053, the ventral surfaces of the caudal centra each possess a deep fossa that becomes progressively shallower posteriorly through the series. In several anterior and proximal‐middle elements, this ventral fossa is subdivided by a midline ridge that separates subfossae.

In the anterior caudal vertebrae, the transverse processes, neural arches and neural spines possess deep fossae, subfossae and accessory fossae. Moving progressively posteriorly through the caudal vertebral sequence, the transverse processes are reduced until they are lost, and the neural arches and spines become less complex. Fossae on the neural arches and spines extend into the middle and posterior elements, but the presence of accessory fossae is sporadic, that is some more posteriorly placed elements possess accessory fossae that are absent in more anteriorly placed ones. The anterior surfaces of each neural arch of the anterior and proximal‐middle caudal vertebrae are deeply excavated by centroprezygapophyseal fossae (CPRFs) located ventromedially to the prezygapophyses, and PRCDFs located laterally to the CPRFs. These fossae are absent in all more posteriorly placed elements. The posterior surfaces of the neural arch of each anterior caudal vertebra possess a POCDF ventrolateral to each postzygapophysis. A proximal‐middle caudal vertebra (MB.R.2956.4) possesses one POCDF positioned ventrolateral to the left postzygapophysis, whereas all further posteriorly positioned elements lack a POCDF. SDFs are present on the lateral surfaces of the anterior–proximal‐middle neural spines, and all anterior–posterior caudal neural spines possess a SPRF. A SPOF likely occurs in all anterior–posterior elements, but incomplete preservation of the neural spine inhibits assessment of the presence /or absence of this fossa in some elements.

**FIGURE 7:**
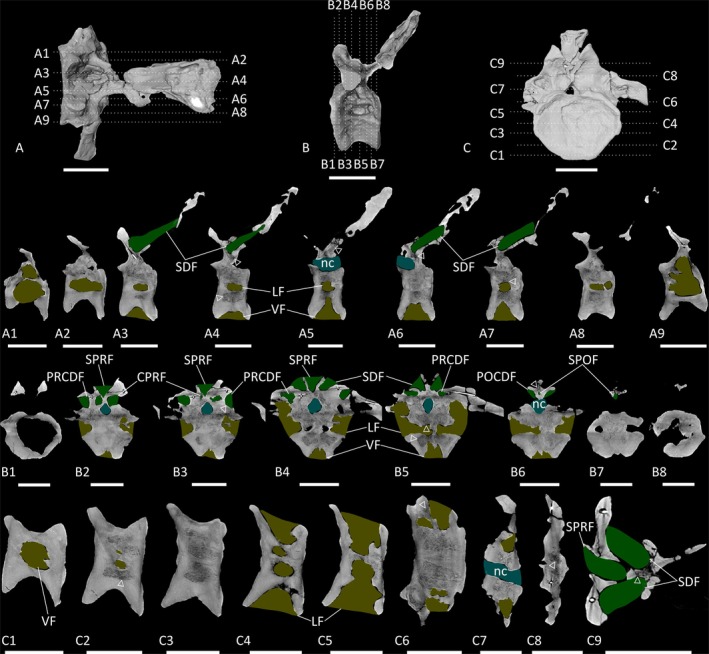
CT scan of *Tornieria africana* anterior caudal vertebra MB.R. 2956.1. (A–C) 3D rendering (A) dorsal view, (B) left lateral view, (C) anterior view; (A1–A9) CT scan slices in parasagittal cross‐section, (B1–B8) CT scan slices in transverse cross‐section, (C1–C9) CT scan slices in horizontal cross‐section. CPRF, centroprezygapophyseal fossa; LF, lateral fossa; nc, neural canal; POCDF, postzygapophyseal centrodiapophyseal fossa; PRCDF, prezygapophyseal centrodiapophyseal fossa; SDF, spinodiapophyseal fossa; SPOF, spinopostzygapophyseal fossa; SPRF, spinoprezygapophyseal fossa; VF, ventral fossa. Key: Blue highlight, neural canal; green highlight, neural spine fossa; yellow highlight, centrum fossa; white arrow, pneumatic bone texture. Scale bars = 100 mm.

**FIGURE 8:**
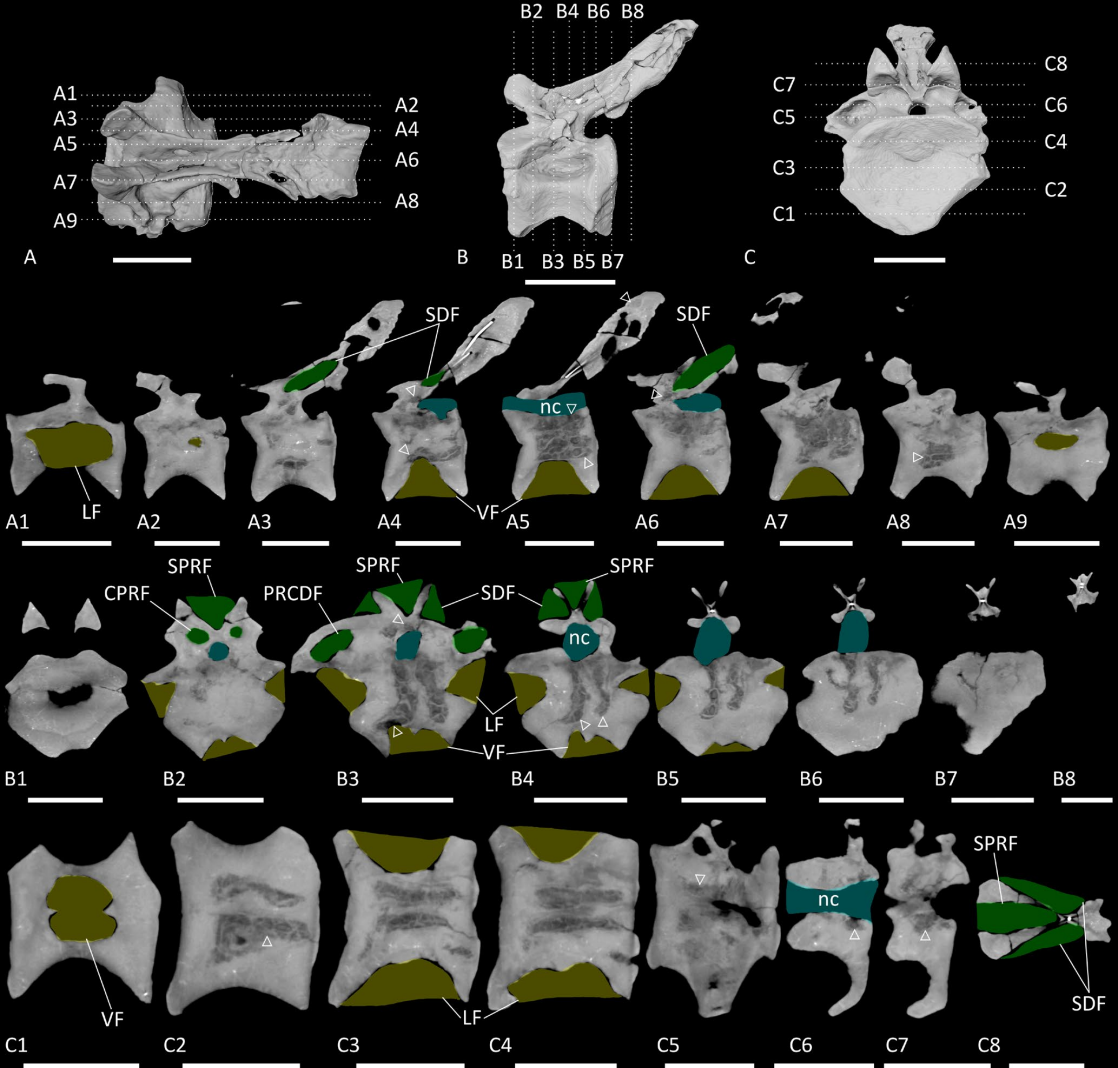
CT scan of *Tornieria africana* middle caudal vertebra MB.R.2956.4. (A–C) 3D rendering (A) dorsal view, (B) left lateral view, (C) anterior view; (A1–A9) CT scan slices in parasagittal cross‐section, (B1–B8) CT scan slices in transverse cross‐section, (C1–C8) CT scan slices in horizontal cross‐section. CPRF, centroprezygapophyseal fossa; LF, lateral fossa; nc, neural canal; PRCDF, prezygapophyseal centrodiapophyseal fossa; SDF, spinodiapophyseal fossa; SPRF, spinoprezygapophyseal fossa; VF, ventral fossa. Key: Blue highlight, neural canal; green highlight, neural spine fossa; yellow highlight, centrum fossa; white arrow, pneumatic bone texture. Scale bars = 100 mm.

**FIGURE 9:**
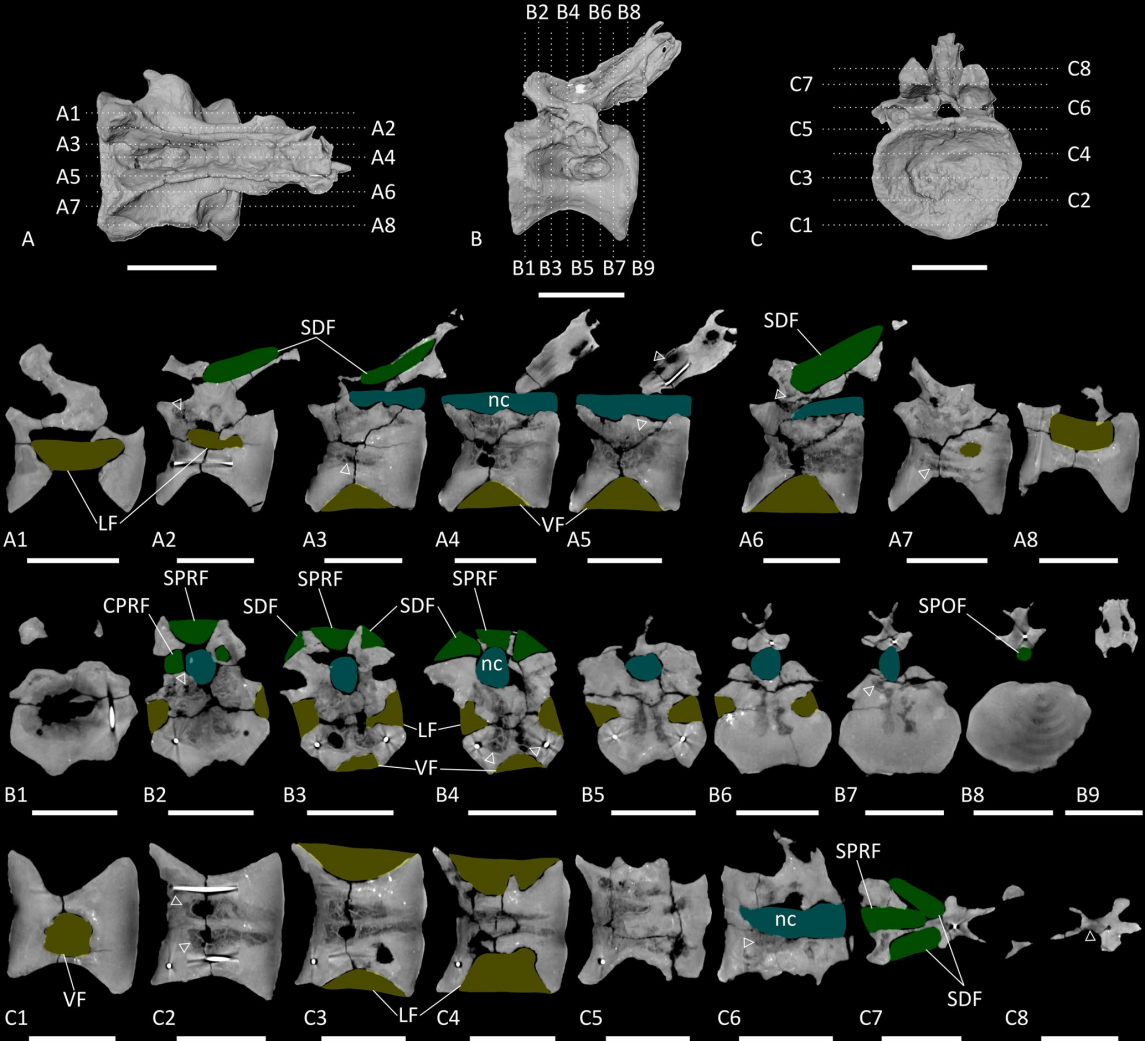
CT scan of *Tornieria africana* middle caudal vertebra MB.R.2956.5. (A–C) 3D rendering (A) dorsal view, (B) left lateral view, (C) anterior view; (A1–A8) CT scan slices in parasagittal cross‐section, (B1–B9) CT scan slices in transverse cross‐section, (C1–C8) CT scan slices in horizontal cross‐section. CPRF, centroprezygapophyseal fossa; LF, lateral fossa; nc, neural canal; SDF, spinodiapophyseal fossa; SPOF, spinopostzygapophyseal fossa; SPRF, spinoprezygapophyseal fossa; VF, ventral fossa. Key: Blue highlight, neural canal; green highlight, neural spine fossa; yellow highlight, centrum fossa; white arrow, pneumatic bone texture. Scale bars = 100 mm.

**FIGURE 10:**
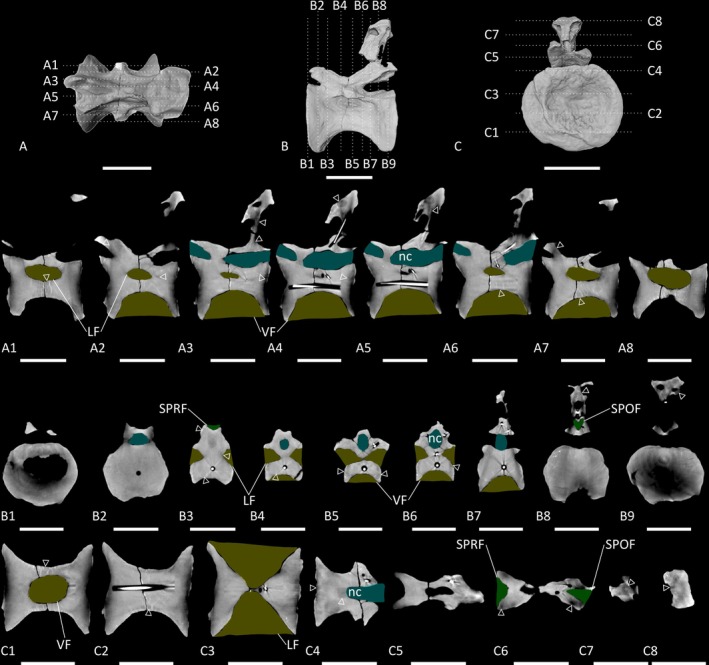
CT scan of *Tornieria africana* middle caudal vertebra MB.R.2956.6. (A–C) 3D rendering (A) dorsal view, (B) left lateral view, (C) anterior view; (A1–A8) CT scan slices in parasagittal cross‐section, (B1–B9) CT scan slices in transverse cross‐section, (C1–C8) CT scan slices in horizontal cross‐section. LF, lateral fossa; nc, neural canal; SPOF, spinopostzygapophyseal fossa; SPRF, spinoprezygapophyseal fossa; VF, ventral fossa. Key: Blue highlight, neural canal; green highlight, neural spine fossa; yellow highlight, centrum fossa; white arrow, pneumatic bone texture. Scale bars = 100 mm.

**FIGURE 11:**
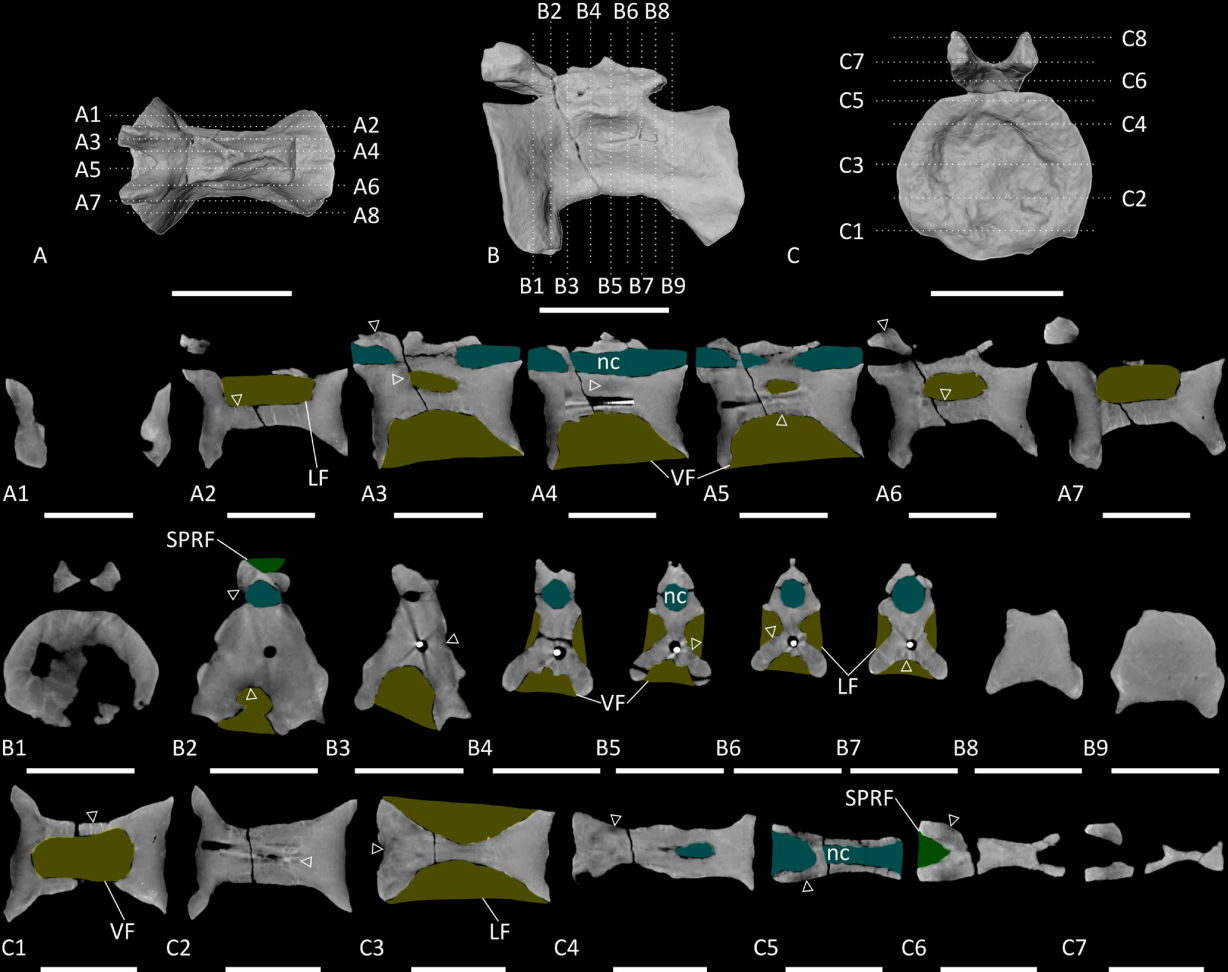
CT scan of *Tornieria africana* posterior caudal vertebra MB.R.2956.12. (A–C) 3D rendering (A) dorsal view, (B) left lateral view, (C) anterior view; (A1–A7) CT scan slices in parasagittal cross‐section, (B1–B9) CT scan slices in transverse cross‐section, (C1–C7) CT scan slices in horizontal cross‐section. LF, lateral fossa; nc, neural canal; SPRF, spinoprezygapophyseal fossa; VF, ventral fossa. Key: Blue highlight, neural canal; green highlight, neural spine fossa; yellow highlight, centrum fossa; white arrow, pneumatic bone texture. Scale bars = 100 mm.

**FIGURE 12:**
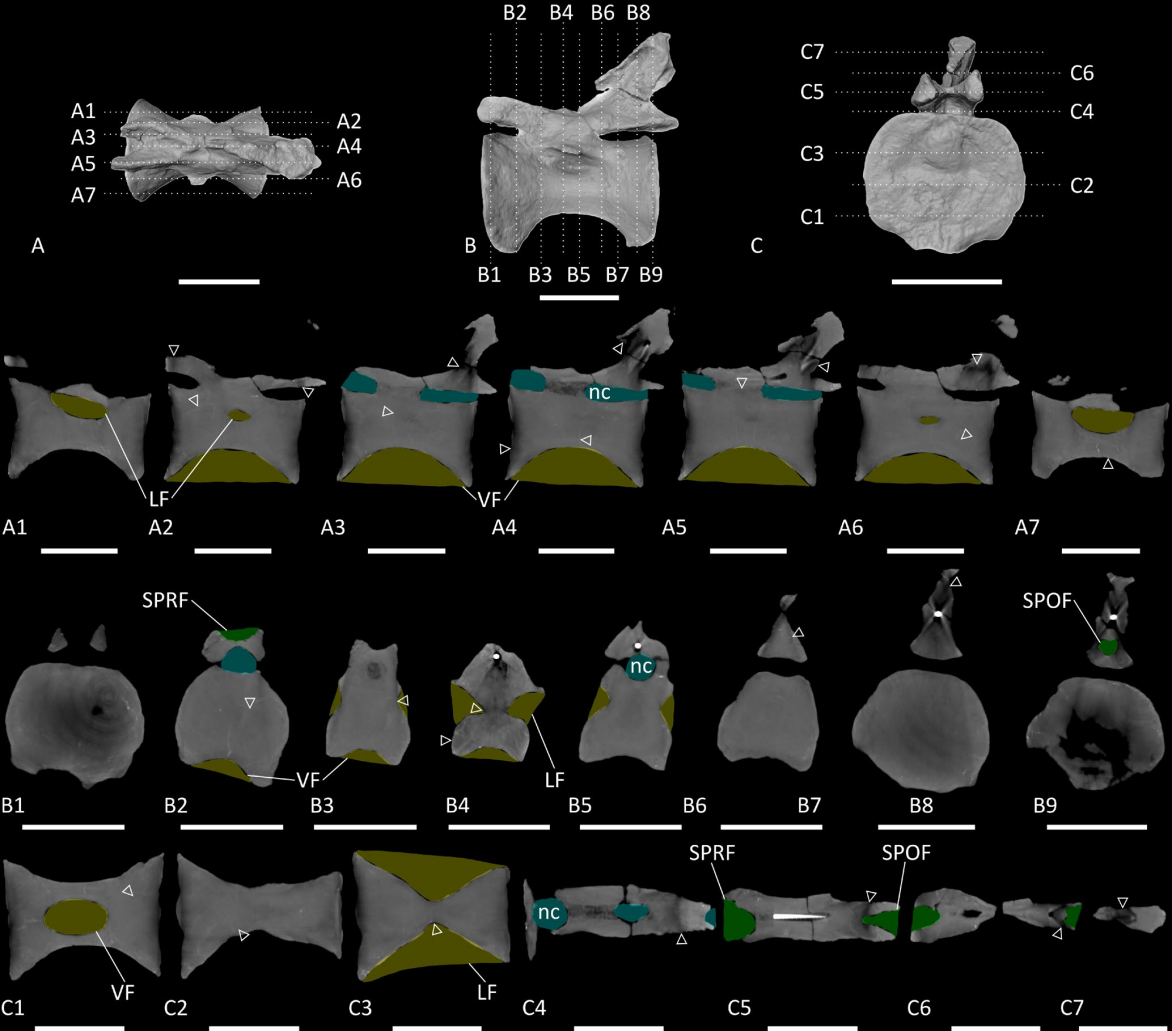
CT scan of *Tornieria africana* posterior caudal vertebra MB.R.2956.14. (A–C) 3D rendering (A) dorsal view, (B) left lateral view, (C) anterior view; (A1–A7) CT scan slices in parasagittal cross‐section, (B1–B9) CT scan slices in transverse cross‐section, (C1–C7) CT scan slices in horizontal cross‐section. LF, lateral fossa; nc, neural canal; SPOF, spinopostzygapophyseal fossa; SPRF, spinoprezygapophyseal fossa; VF, ventral fossa. Key: Blue highlight, neural canal; green highlight, neural spine fossa; yellow highlight, centrum fossa; white arrow, pneumatic bone texture. Scale bars = 100 mm.

**FIGURE 13:**
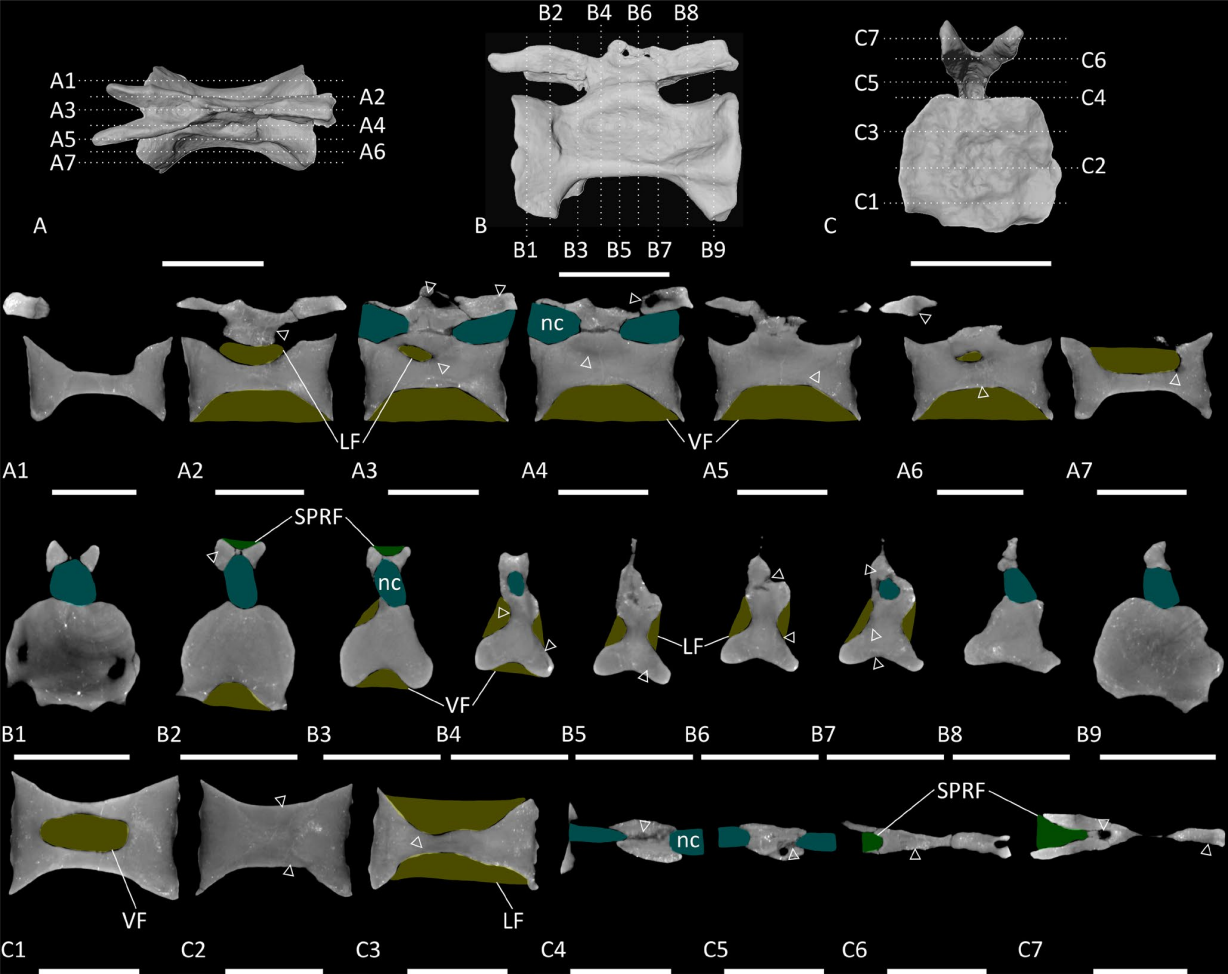
CT scan of *Tornieria africana* posterior caudal vertebra MB.R.2956.19. (A–C) 3D rendering (A) dorsal view, (B) left lateral view, (C) anterior view; (A1–A7) CT scan slices in parasagittal cross‐section, (B1–B9) CT scan slices in transverse cross‐section, (C1–C7) CT scan slices in horizontal cross‐section. LF, lateral fossa; nc, neural canal; SPRF, spinoprezygapophyseal fossa; VF, ventral fossa. Key: Blue highlight, neural canal; green highlight, neural spine fossa; yellow highlight, centrum fossa; white arrow, pneumatic bone texture. Scale bars = 100 mm.

CT scans reveal that the anterior–posterior caudal vertebrae are pneumatic (Figures 7, 8, 9, 10, 11, 12, 13). The distinction between bone and internal pneumatic chambers is difficult to determine in MB.R.2956.6, MB.R.2956.12, MB.R.2956.14 and MB.R.2956.19 due to low contrast resolution. Except for MB.R.2956.19, each element possesses one or more metal rods that contribute to the reduction in contrast resolution due to beam hardening. However, close inspection of the CT scan slices of these elements reveals internal pneumatic chambers.

In the anterior and proximal‐middle (MB.R.2956.1, MB.R.2956.4 and MB.R.2956.5) caudal centra, internal chambers communicate externally with the lateral and ventral fossae, as well as the floor of the neural canal. Chambers in the centra extend dorsally into the neural arches, the zygapophyses and the neural spine via the roof of the neural canal and the external fossae. The pneumatic chambers inside the anterior caudal vertebra (MB.R.2956.1; Figure 7) are more extensive than those of the proximal‐middle caudal vertebrae (MB.R.2956.4 and MB.R.2956.5; Figures 8 and 9). In MB.R.2956.1, the chambers occupy most of the space inside the centrum, whereas in MB.R.2956.4 and MB.R.2956.5, the centrum possesses two prominent parallel parasagittal pneumatic tubes (Figures 8 and 9). These tubes communicate with each other and the surrounding pneumatic bone, and they comprise smaller pneumatic chambers, as in the anterior element.

The distal‐middle (MB.R.2956.6; Figure 10) and proximal‐posterior (MB.R.2956.12 and MB.R.2956.14; Figures 11 and 12) caudal vertebrae possess separate internal pneumatic chambers that differ in size, do not bifurcate and communicate with external fossae and the neural canal, as described above for the more anterior elements. A thin layer of compact bone separates each chamber. In the distal‐posterior caudal vertebra (MB.R.2956.19; Figure 13), the low contrast resolution is especially prevalent, but we tentatively interpret the element as pneumatic. There indeed appear to be separate internal pneumatic chambers in the centrum (see Figure 13B4–B7) that communicate with the lateral fossa and the floor of the neural canal. The neural arches and reduced neural spine appear to be pneumatised (see Figure 13A2–A5,B5–B7,C5–C7) via the dorsal extension of the pneumatic chambers within the centrum, the roof of the neural canal and an accessory fossa on the lateral surfaces of the neural spine.

#### 3.1.6 Giraffatitan brancai

**FIGURE 14:**
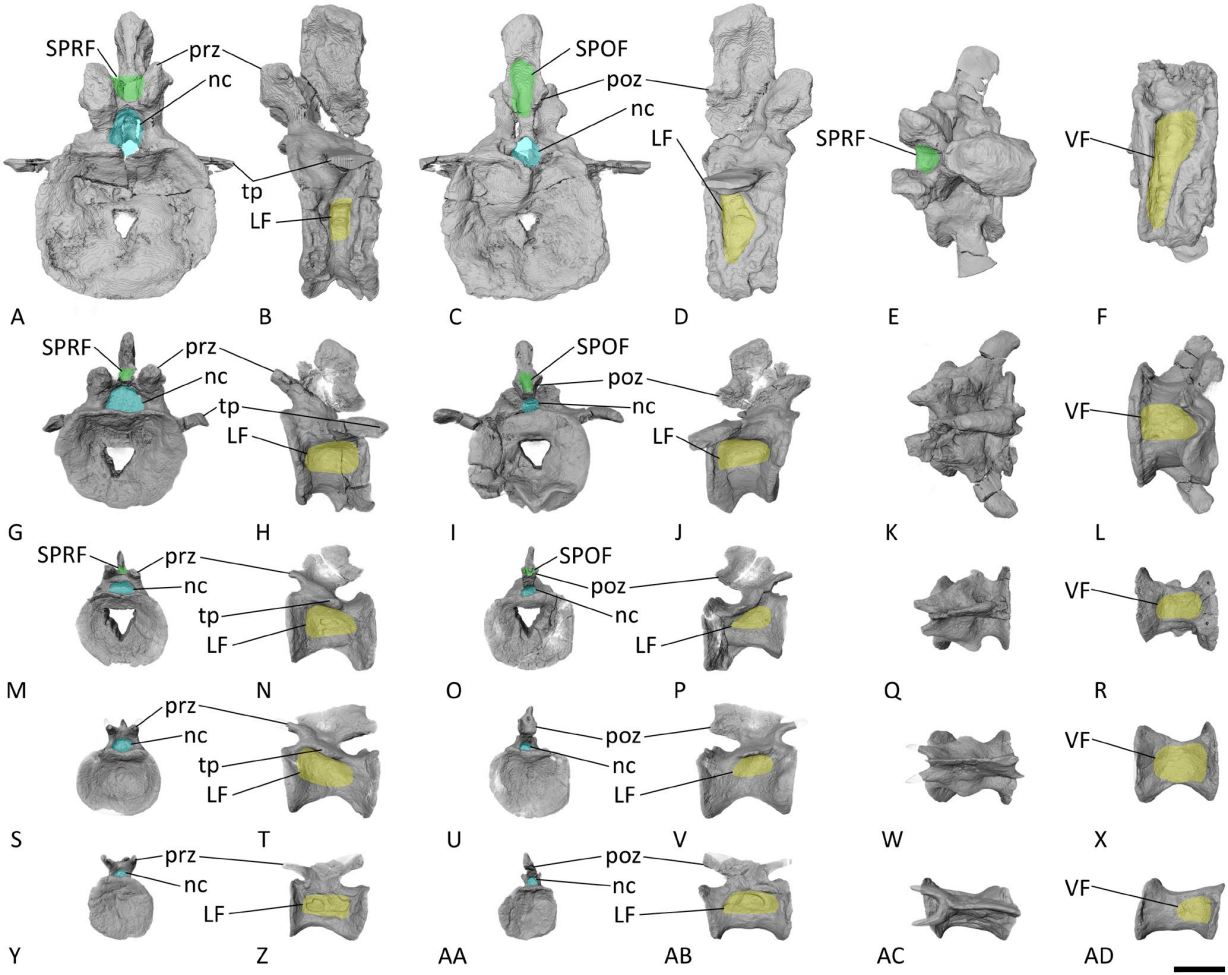
3D renderings of CT scanned *Giraffatitan brancai* caudal vertebrae MB.R.5000. (A–F) Caudal vertebra 2 MB.R.5000.1, (G–L) Caudal vertebra 7 MB.R.5000.6, (M–R) Caudal vertebra 15 MB.R.5000.14, (S–X) Caudal vertebra 19 MB.R.5000.18, (Y–AD) Caudal vertebra 24 MB.R.5000.23; (A, G, M, S, Y) anterior view, (B, H, N, T, Z) left lateral view, (C, I, O, U, AA) posterior view, (D, J, P, V, AB) right lateral view, (E, K, Q, W, AC) dorsal view, (F, L, R, X, AD) ventral view. LF, lateral fossa; nc, neural canal; poz, postzygapophysis; prz, prezygapophysis; SPOF, spinopostzygapophyseal fossa; SPRF, spinoprezygapophyseal fossa; tp, transverse process; VF, ventral fossa. Key: Blue highlight, neural canal; green highlight, neural spine fossa; yellow highlight, centrum fossa. Scale bar = 100 mm.

**FIGURE 15:**
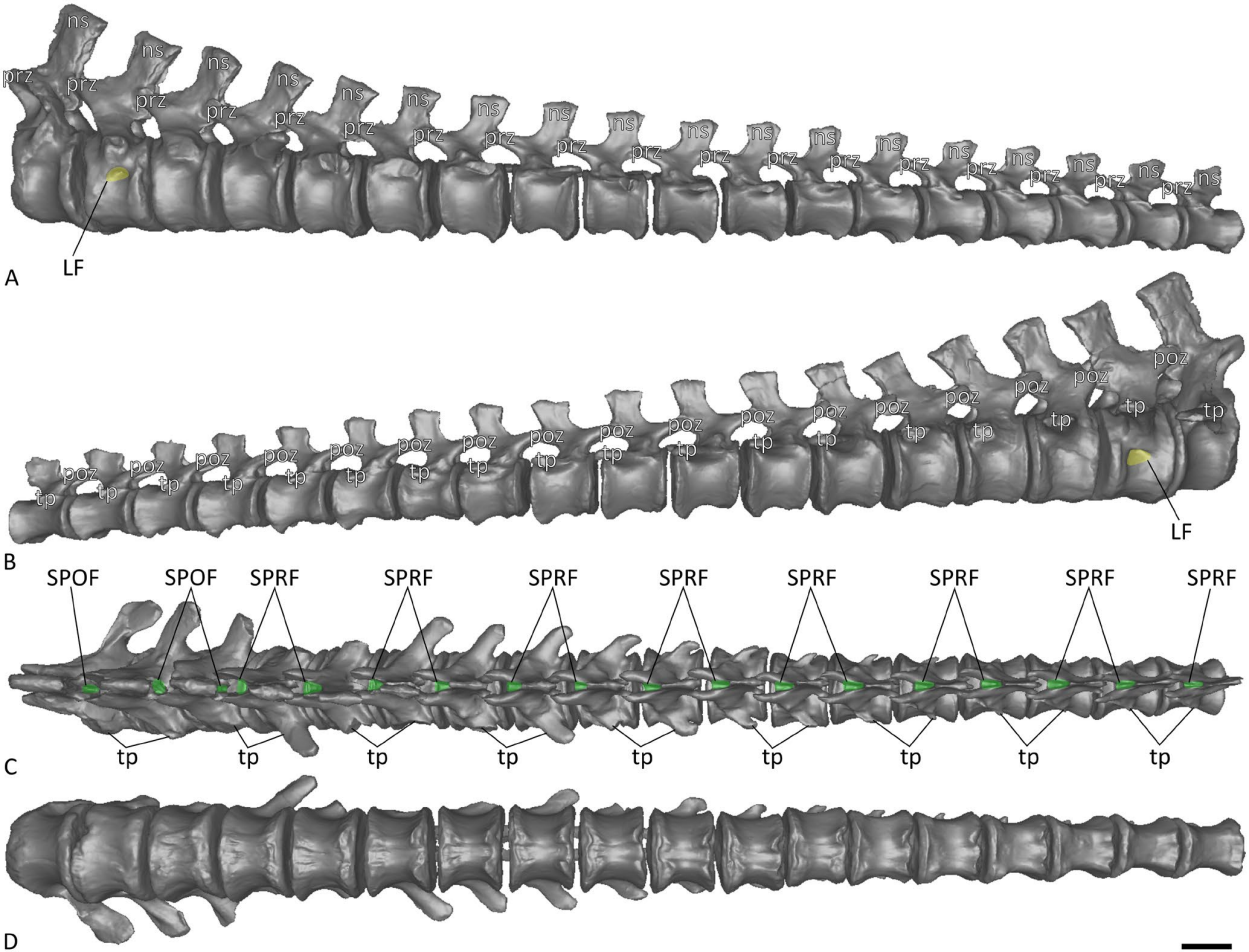
Surface scans of *Giraffatitan brancai* caudal vertebrae 1–18 MB.R.2921. (A) left lateral view, (B) right lateral view, (C) dorsal view, (D) ventral view. LF, lateral fossa; ns, neural spine; poz, postzygapophysis; prz, prezygapophysis; SPOF, spinopostzygapophyseal fossa; SPRF, spinoprezygapophyseal fossa; tp, transverse process. Key: Green highlight, neural spine fossa; yellow highlight, centrum fossa. Scale bar = 100 mm.

The external pneumatic features of the three caudal vertebral series of *Giraffatitan* (MB.R.2921, MB.R.3736 and MB.R.5000) have been extensively described by Wedel and Taylor (2013) and Díez Díaz et al. (2020). As such, the description below is primarily based upon the five anterior–middle caudal vertebrae of MB.R.5000 that were CT scanned (Ca2, MB.R.5000.1; Ca7, MB.R.5000.6; Ca15, MB.R.5000.14; Ca19, MB.R.5000.18; Ca24, MB.R.5000.23; Figure 14), with any differences in observations noted. Each caudal centrum of MB.R.5000 possesses a lateral fossa and a ventral fossa, differing in relative size, definition and depth, that do not follow a distinct pattern (e.g. diminishing in size posteriorly through the series). This condition differs from that of MB.R.2921 (Figure 15) and MB.R.3736, both of which only possess a lateral fossa on Ca2 (Wedel & Taylor, 2013). Furthermore, this observation differs from that reported by Wedel and Taylor (2013: fig. 8), who recognised a left lateral fossa only in MB.R.5000.14 and MB.R.5000.18, and a right lateral fossa only in MB.R.5000.6. Finally, MB.R.5000.1, MB.R.5000.6 and MB.R.5000.14 each possess a SPRF and a SPOF, whereas MB.R.5000.18 and MB.R.5000.23 lack such fossae (Figure 14).

**FIGURE 16:**
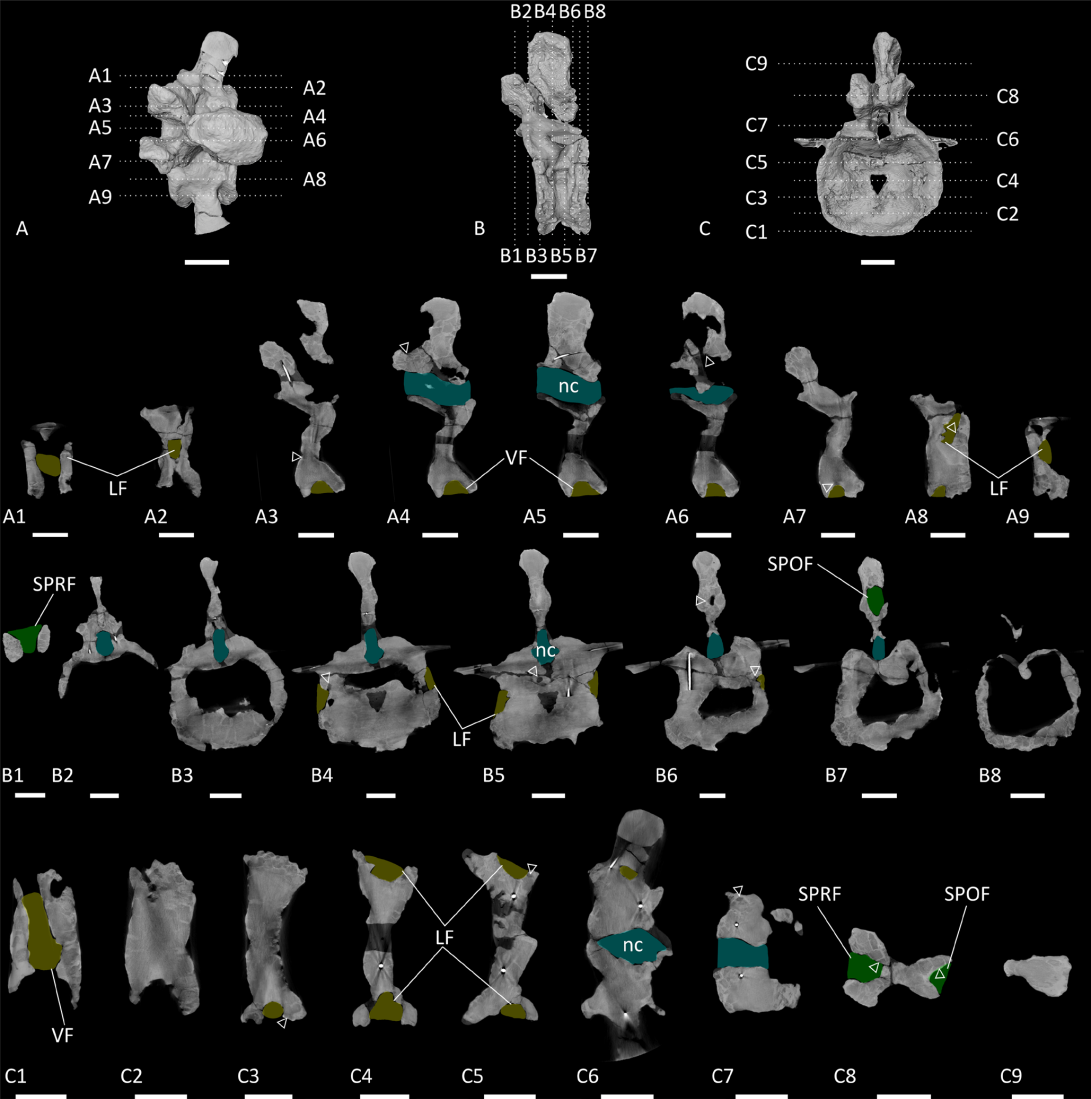
CT scan of *Giraffatitan brancai* caudal vertebra 2 MB.R.5000.1. (A–C) 3D rendering (A) dorsal view, (B) left lateral view, (C) anterior view; (A1–A9) CT scan slices in parasagittal cross‐section, (B1–B8) CT scan slices in transverse cross‐section, (C1–C9) CT scan slices in horizontal cross‐section. LF, lateral fossa; nc, neural canal; SPOF, spinopostzygapophyseal fossa; SPRF, spinoprezygapophyseal fossa; VF, ventral fossa. Key: Blue highlight, neural canal; green highlight, neural spine fossa; yellow highlight, centrum fossa; white arrow, pneumatic bone texture. Scale bars = 100 mm.

**FIGURE 17:**
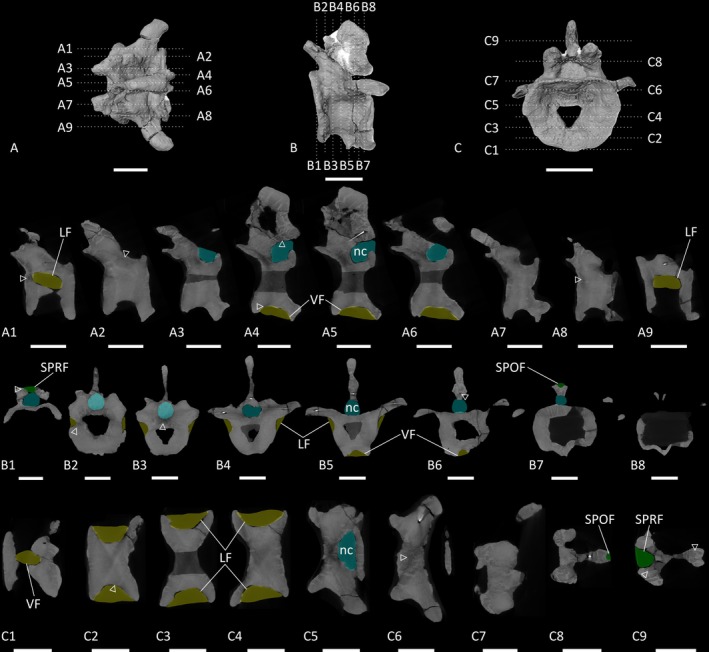
CT scan of *Giraffatitan brancai* caudal vertebra 7 MB.R.5000.6. (A–C) 3D rendering (A) dorsal view, (B) left lateral view, (C) anterior view; (A1–A9) CT scan slices in parasagittal cross‐section, (B1–B8) CT scan slices in transverse cross‐section, (C1–C9) CT scan slices in horizontal cross‐section. LF, lateral fossa; nc, neural canal; SPOF, spinopostzygapophyseal fossa; SPRF, spinoprezygapophyseal fossa; VF, ventral fossa. Key: Blue highlight, neural canal; green highlight, neural spine fossa; yellow highlight, centrum fossa; white arrow, pneumatic bone texture. Scale bars = 100 mm.

**FIGURE 18:**
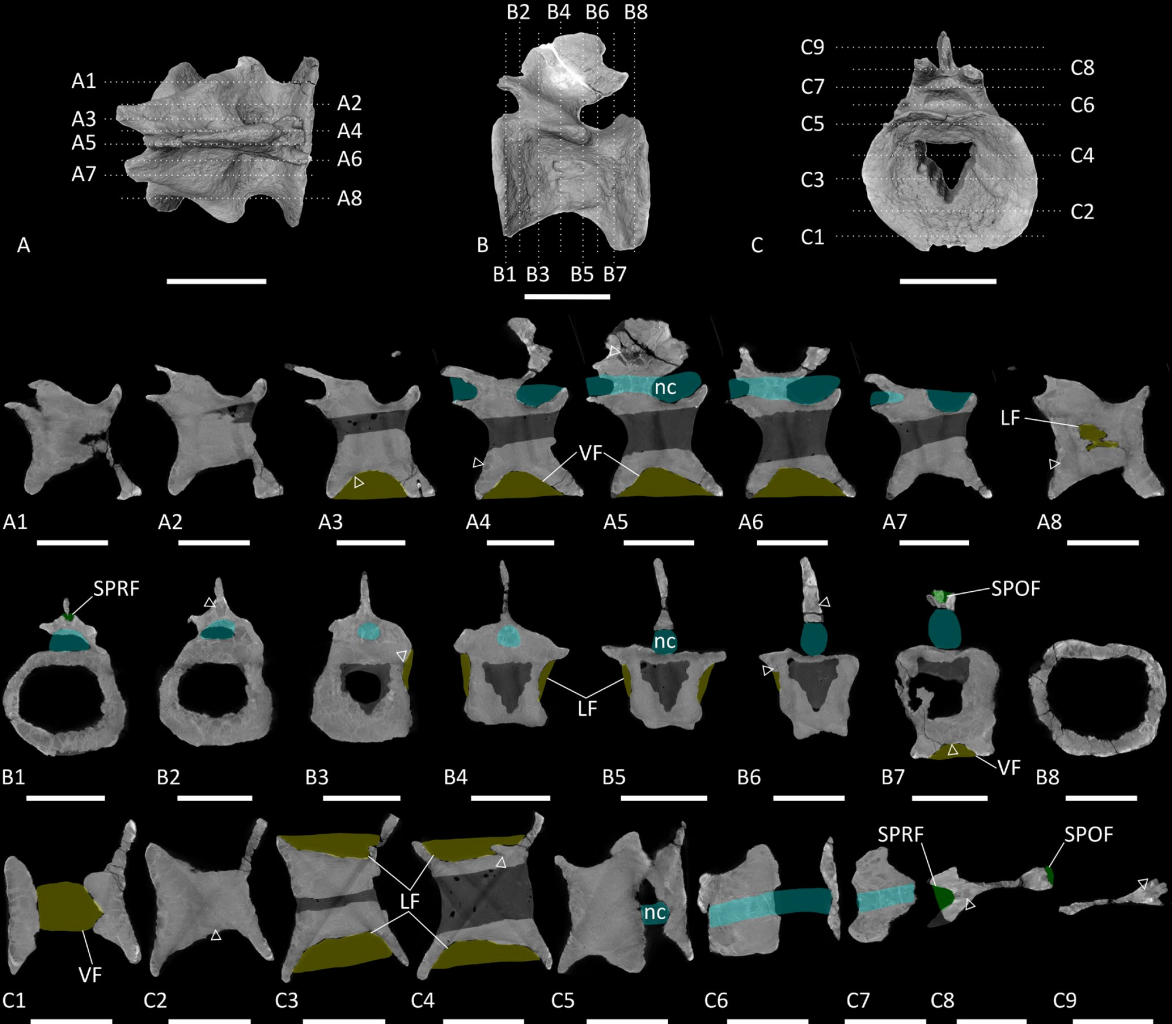
CT scan of *Giraffatitan brancai* caudal vertebra 15 MB.R.5000.14. (A–C) 3D rendering (A) dorsal view, (B) left lateral view, (C) anterior view; (A1–A8) CT scan slices in parasagittal cross‐section, (B1–B8) CT scan slices in transverse cross‐section, (C1–C9) CT scan slices in horizontal cross‐section. LF, lateral fossa; nc, neural canal; VF, ventral fossa. Key: Blue highlight, neural canal; green highlight, neural spine fossa; yellow highlight, centrum fossa; white arrow, pneumatic bone texture. Scale bars = 100 mm.

**FIGURE 19:**
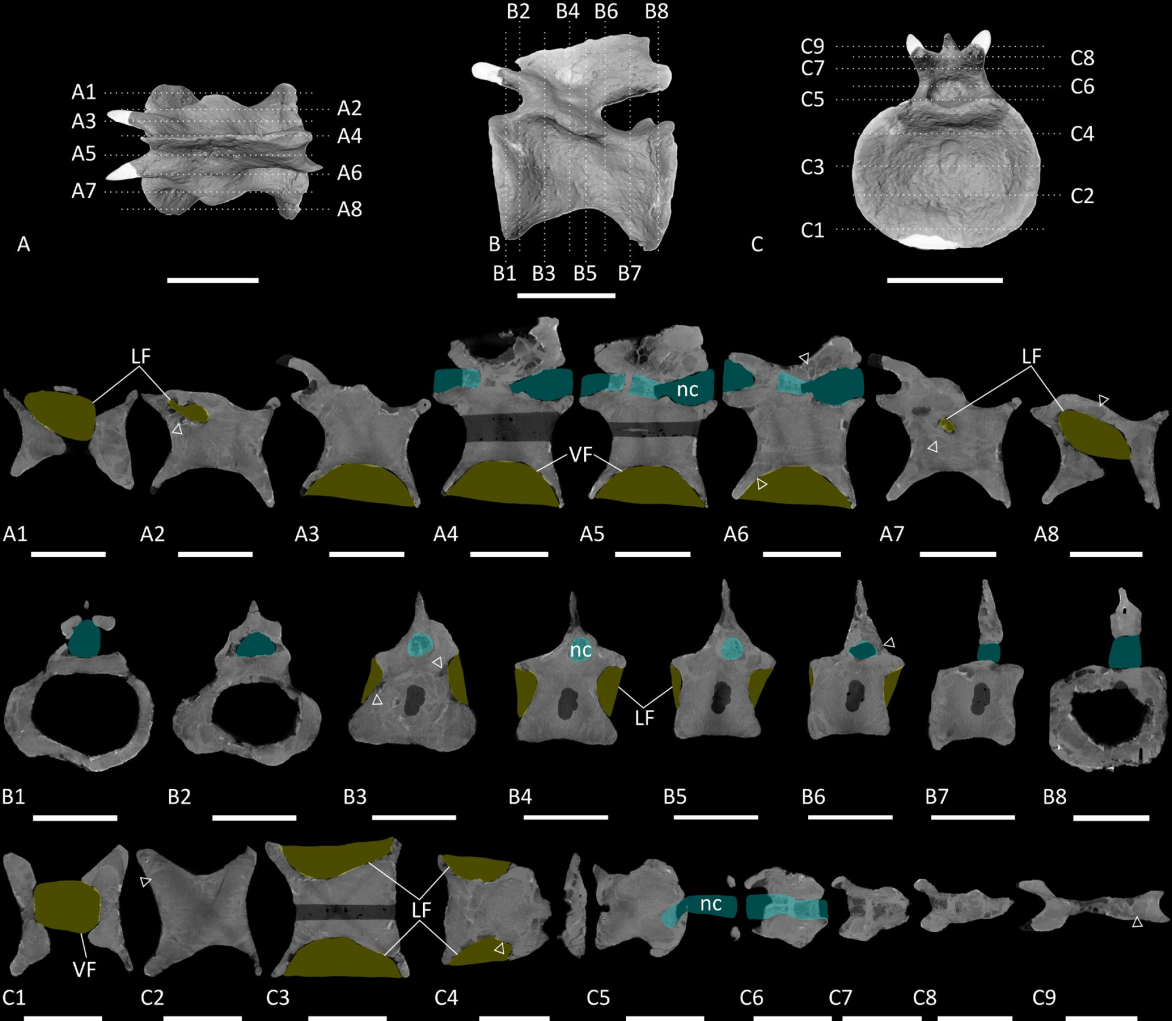
CT scan of *Giraffatitan brancai* caudal vertebra 19 MB.R.5000.18. (A–C) 3D rendering (A) dorsal view, (B) left lateral view, (C) anterior view; (A1–A8) CT scan slices in parasagittal cross‐section, (B1–B8) CT scan slices in transverse cross‐section, (C1–C9) CT scan slices in horizontal cross‐section. LF, lateral fossa; nc, neural canal; VF, ventral fossa. Key: Blue highlight, neural canal; yellow highlight, centrum fossa; white arrow, pneumatic bone texture. Scale bars = 100 mm.

**FIGURE 20:**
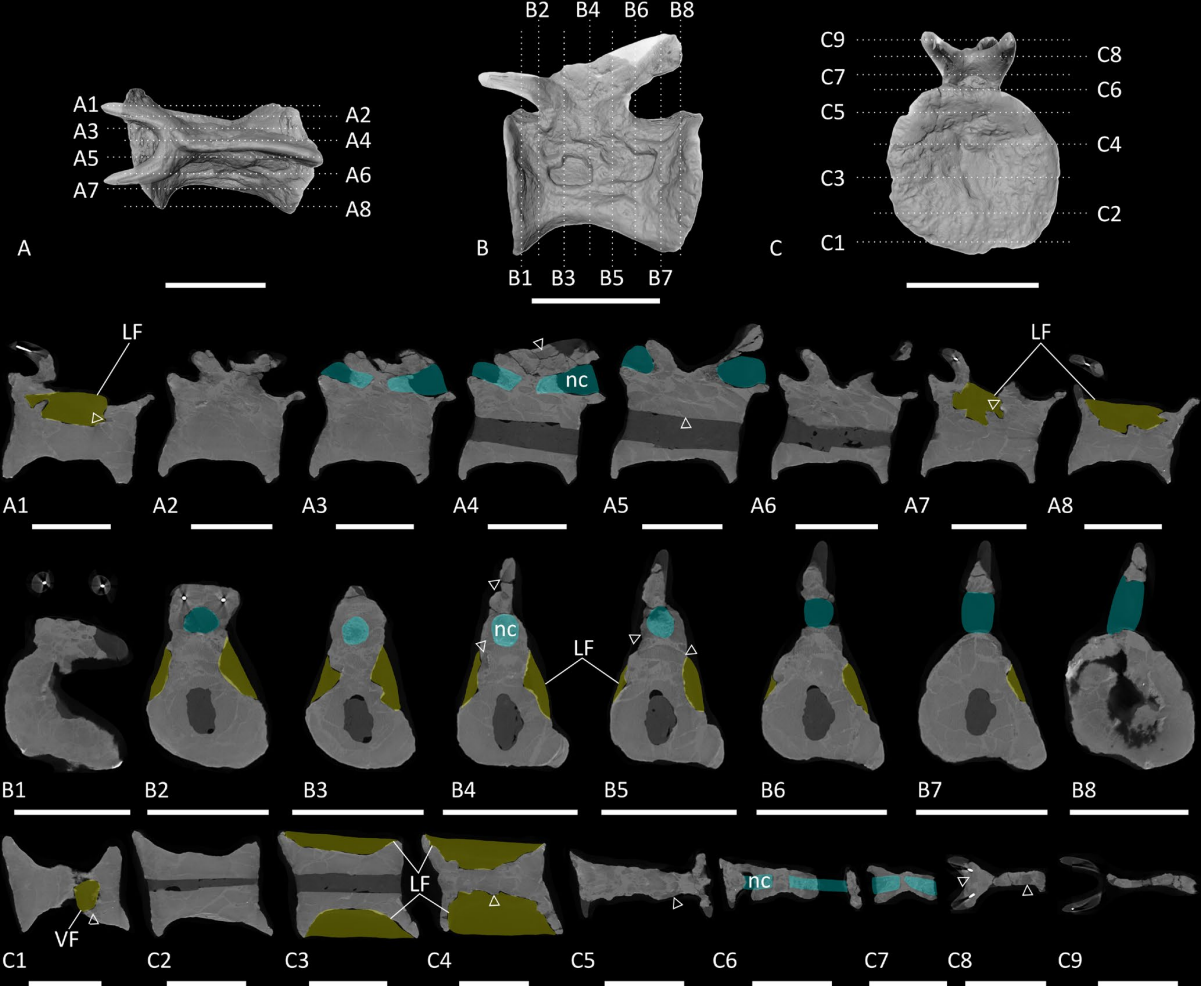
CT scan of *Giraffatitan brancai* caudal vertebra 24 MB.R.5000.23. (A–C) 3D rendering (A) dorsal view, (B) left lateral view, (C) anterior view; (A1–A8) CT scan slices in parasagittal cross‐section, (B1–B8) CT scan slices in transverse cross‐section, (C1–C9) CT scan slices in horizontal cross‐section. LF, lateral fossa; nc, neural canal; VF, ventral fossa. Key: Blue highlight, neural canal; yellow highlight, centrum fossa; white arrow, pneumatic bone texture. Scale bars = 100 mm.

CT scans reveal that each element possesses metal rods within the centrum and/or the neural arches (Figures 16, 17, 18, 19, 20). Furthermore, an anteroposteriorly elongate section of bone has been removed from the centre of each centrum, owing to previous iterations of how the tail was mounted. The internal bone texture of each element is pneumatic, defined by chambers of varying sizes. These chambers are separated from each other by compact bone of different thicknesses, and they do not bifurcate. At first glance, the neural arches appear to possess more pneumatic chambers and vascularisation than the centra. However, close examination of CT scan slices at different resolutions reveals that this is a superficial difference owing to a higher density in the internal matrix/bone texture of the centra than in the neural arches.

The external fossae of all CT scanned elements communicate internally with pneumatic chambers. In MB.R.5000.1 (Figure 16), MB.R.5000.6 (Figure 17) and MB.R.5000.14 (Figure 18), communication is via the lateral fossa, ventral fossa, SPRF and SPOF, whereas in MB.R.5000.18 (Figure 19) and MB.R.5000.23 (Figure 20), communication is via the lateral fossa only. The neural canal of each caudal vertebra also appears to communicate ventrally and dorsally with the interiors of the centrum and neural arch, respectively. However, areas around the neural canal of MB.R.5000.1 have undergone some plaster restoration, hindering true interpretation of such communication. Internal chambers extend from the anteroposterior margins of each centrum (e.g. Figure 16B3,B7,C1) as far as the zygapophyses and the dorsal tip of the neural spine (e.g. Figure 16C8–C9). Examination of the internal bone texture of the transverse processes of MB.R.5000.1 and MB.R.5000.6 is hindered by metal rods within both elements. Internal chambers are present in the transverse processes of MB.R.5000.14. However, the reduced tips of the transverse processes of MB.R.5000.18 possess a dense, apneumatic texture.

### 3.2 Review of postcranial skeletal pneumaticity in sauropodomorph dinosaur caudal vertebrae

**FIGURE 21:**
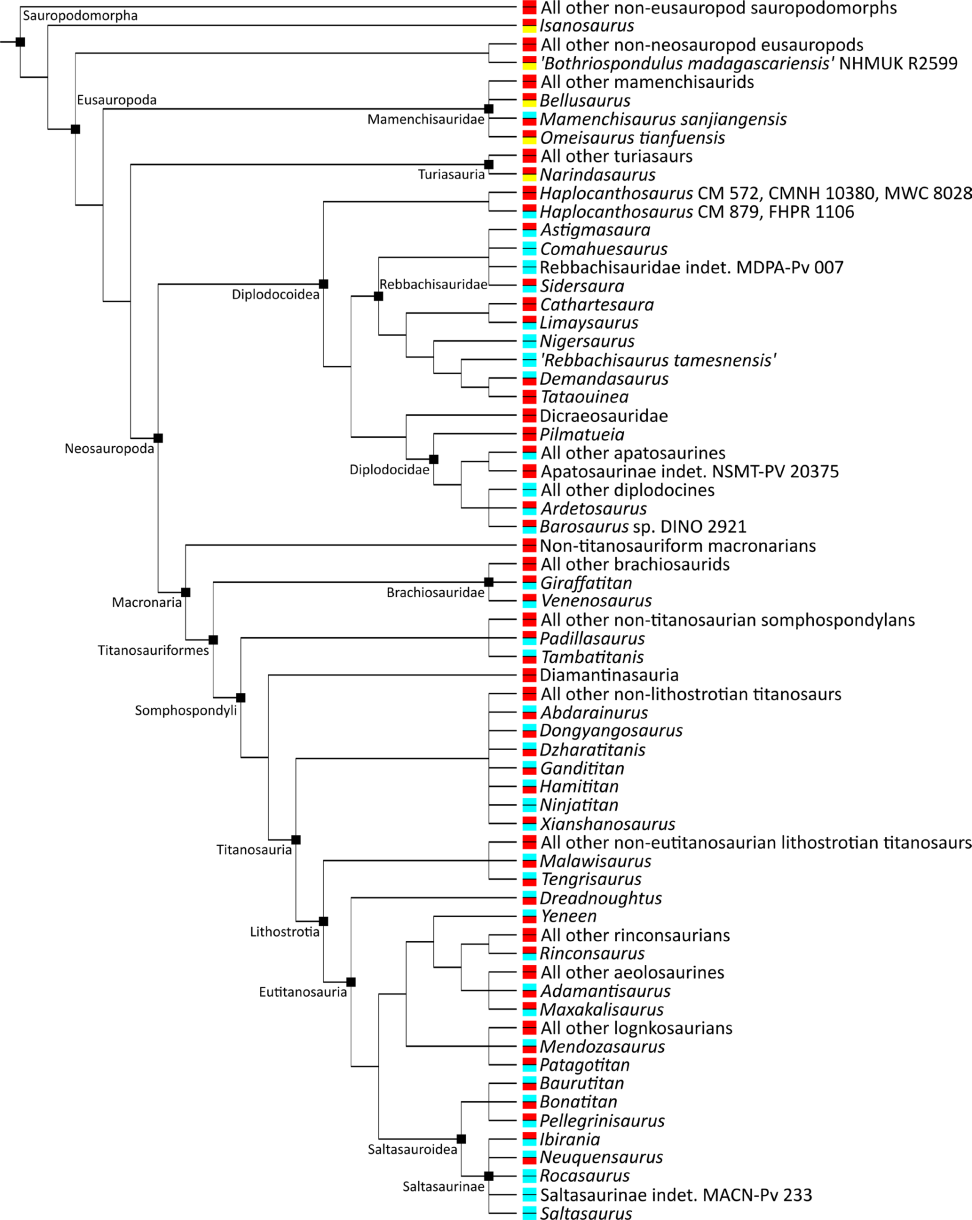
Cladogram of sauropodomorphs with distribution of external evidence for postcranial skeletal pneumaticity in the anterior caudal vertebrae. Phylogenetic positions of included taxa are based on the analyses of Poropat et al. (2023) and Mannion and Moore (2025). Key: Split box bottom half, centrum; split box top half, neural arch; blue, external evidence for PSP present; red, external evidence for PSP absent; yellow, ambiguous external evidence for PSP.

**FIGURE 22:**
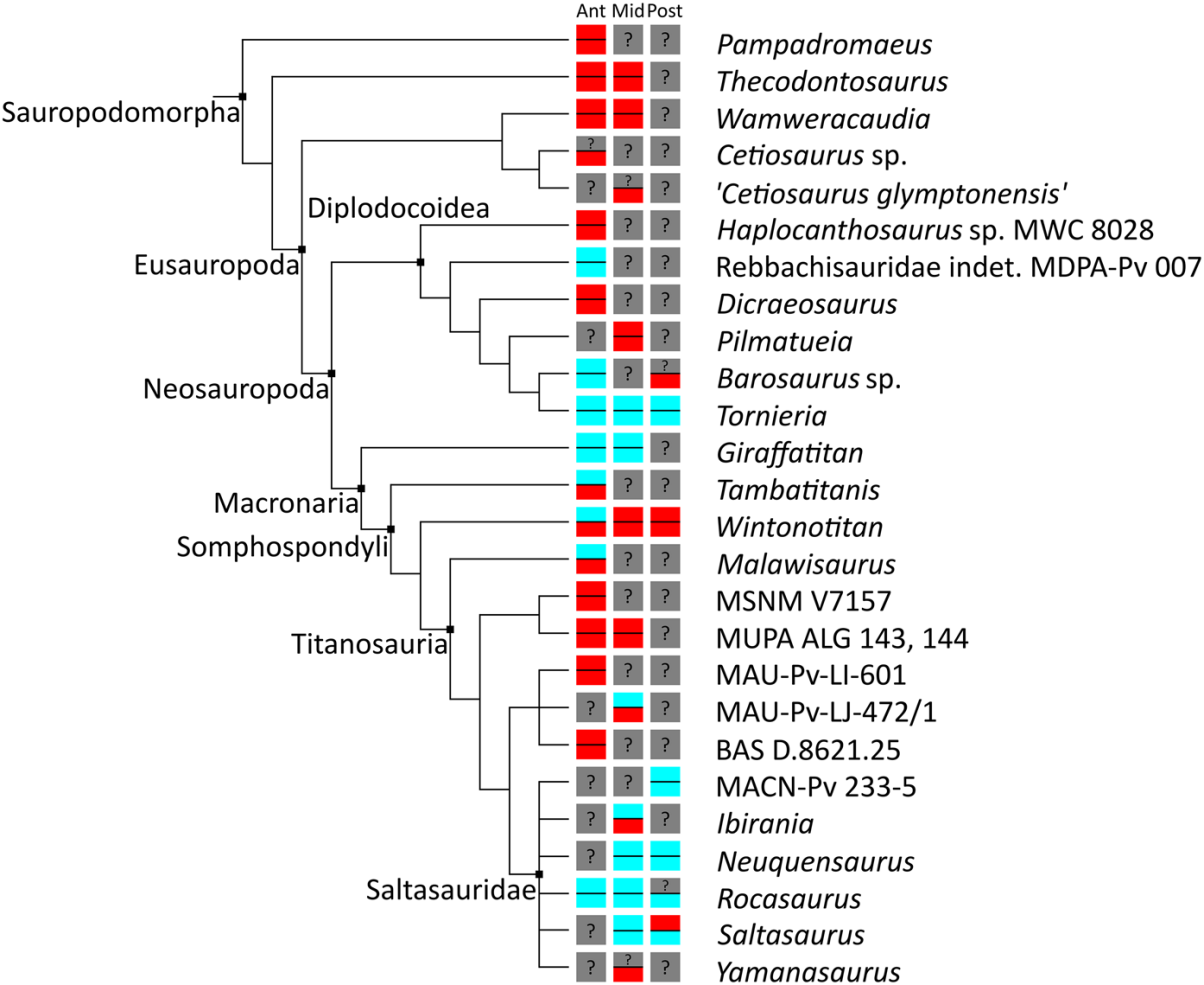
Cladogram of sauropodomorphs with CT scanned caudal vertebrae. Phylogenetic positions of included taxa are based on the analyses of Beccari et al. (2021), Poropat et al. (2023), and Mannion and Moore (2025). Ant, anterior; Mid, middle; Post, posterior. Key: Split box bottom half, centrum; split box top half, neural arch; blue, internal PSP present; grey, missing data; red, internal PSP absent.

Wedel and Taylor (2013) conducted a review of caudal vertebral pneumaticity in ornithodirans, with a focus on sauropodomorph dinosaurs. However, since then, an array of additional sauropodomorph taxa have been named, redescribed, and/or internally imaged, enabling a more comprehensive understanding of the distribution of caudal vertebral pneumaticity in sauropodomorphs. As such, below we present an updated critical appraisal to provide a clearer view of the phylogenetic distribution of caudal vertebral pneumatic features in Sauropodomorpha (Figure 21; Supplemental Data). Data included in this review stem from external evidence for PSP (i.e. the presence/absence of external fossae that might be pneumatic in origin, such as a LPF, subdivided fossae, accessory fossae and surfaces heavily excavated by fossae; Figure 21), and internal evidence for PSP (i.e. direct observation of internal bone texture from internal imaging, sectioning, and natural breaks in bone; Figure 22). If external evidence for caudal vertebral PSP is absent in the anterior elements, it is almost always absent in more posteriorly placed elements. Furthermore, if an anterior element does possess external evidence for PSP, it is almost always absent in the middle and posterior elements. As such, the included external evidence for PSP is based upon the presence and/or absence of pneumatic features in the anterior caudal vertebrae, unless otherwise specified. We have adopted a conservative approach when describing the internal bone texture based solely on natural breaks in specimens; such a description is only presented when a substantial cross‐section of the internal bone texture of an element can be seen.

#### 3.2.1 Non‐eusauropod sauropodomorphs

**FIGURE 23:**
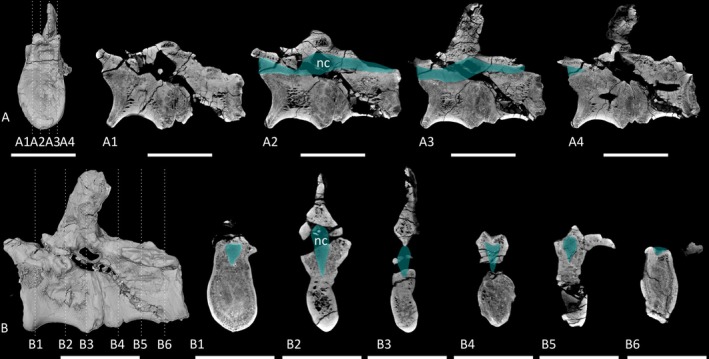
CT scan of *Pampadromaeus barberenai* anterior caudal vertebrae ULBRA‐PVT016. (A, B) 3D rendering (A) anterior view, (B) lateral view; (A1–A4) CT scan slices in parasagittal cross‐section, (B1–B6) CT scan slices in transverse cross‐section. nc, neural canal. Key: Blue highlight, neural canal. Scale bars = 20 mm.

**FIGURE 24:**
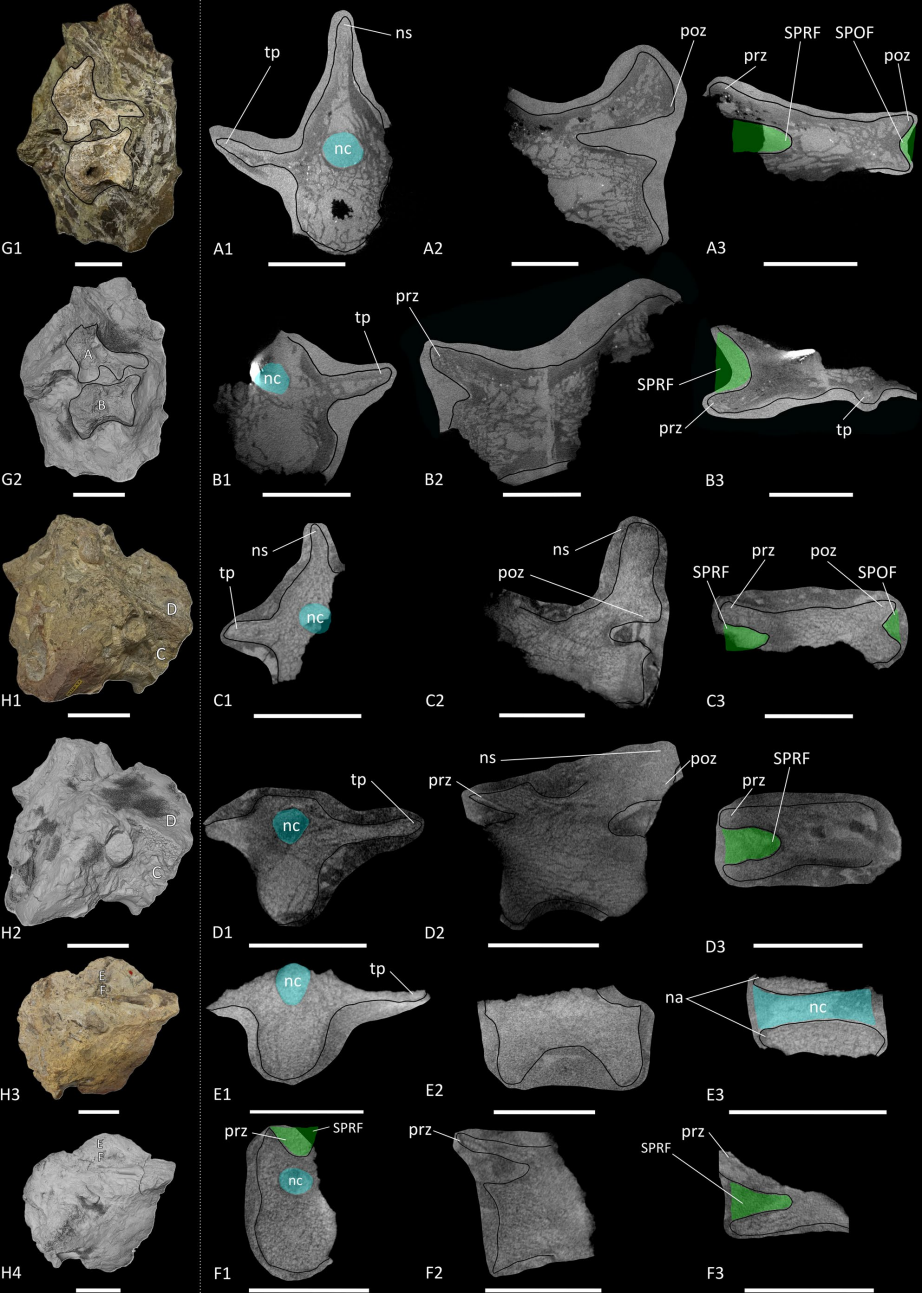
CT scan of *Thecodontosaurus antiquus* caudal vertebrae. (A1–A3) Element A, (B1–B3) element B, (C1–C3) element C, (D1–D3) element D, (E1–E3) element E, (F1–F3) element F; (A1–F1) CT scan transverse cross‐section, (A2–F2) CT scan parasagittal cross‐section, (A3–F3) CT scan horizontal cross‐section; (G1, G2) Cb 4164 block, (H1–H4) Cb 4714 block; (H1, H2) side 1, (H3, H4) side 2; (G1, H1, H3) photograph, (G2, H2, H4) surface scan. Na, neural arch; nc, neural canal; ns, neural spine; poz, postzygapophysis; prz, prezygapophysis; SPOF, spinopostzygapophyseal fossa; SPRF, spinoprezygapophyseal fossa; tp, transverse process. Key: Blue highlight, neural canal; green highlight, neural spine fossa. Scale bars = 20 mm (A–F), 50 mm (G, H).

External evidence for PSP is absent in the caudal vertebrae of all non‐gravisaurian sauropodomorph dinosaurs (Aureliano et al., 2022; Beeston et al., 2026). Within non‐sauropod sauropodomorphs, anterior and anterior–middle caudal vertebrae of *Pampadromaeus barberenai* (Figure 23; Aureliano et al., 2022) and *Thecodontosaurus antiquus* (Figure 24; Beeston et al., 2026), respectively, have been CT scanned. Whereas the caudal vertebrae of *Pampadromaeus* lack external fossae, those of *Thecodontosaurus* possess a SPRF and a SPOF. The external fossae of *Thecodontosaurus* do not communicate with internal chambers. Therefore, in both taxa, the caudal vertebrae possess an apneumatic internal texture, with regions of less densely packed trabeculae (i.e. increased vascularisation) positioned towards the centre of the centra and neural arches (Figures 23 and 24).

**FIGURE 25:**
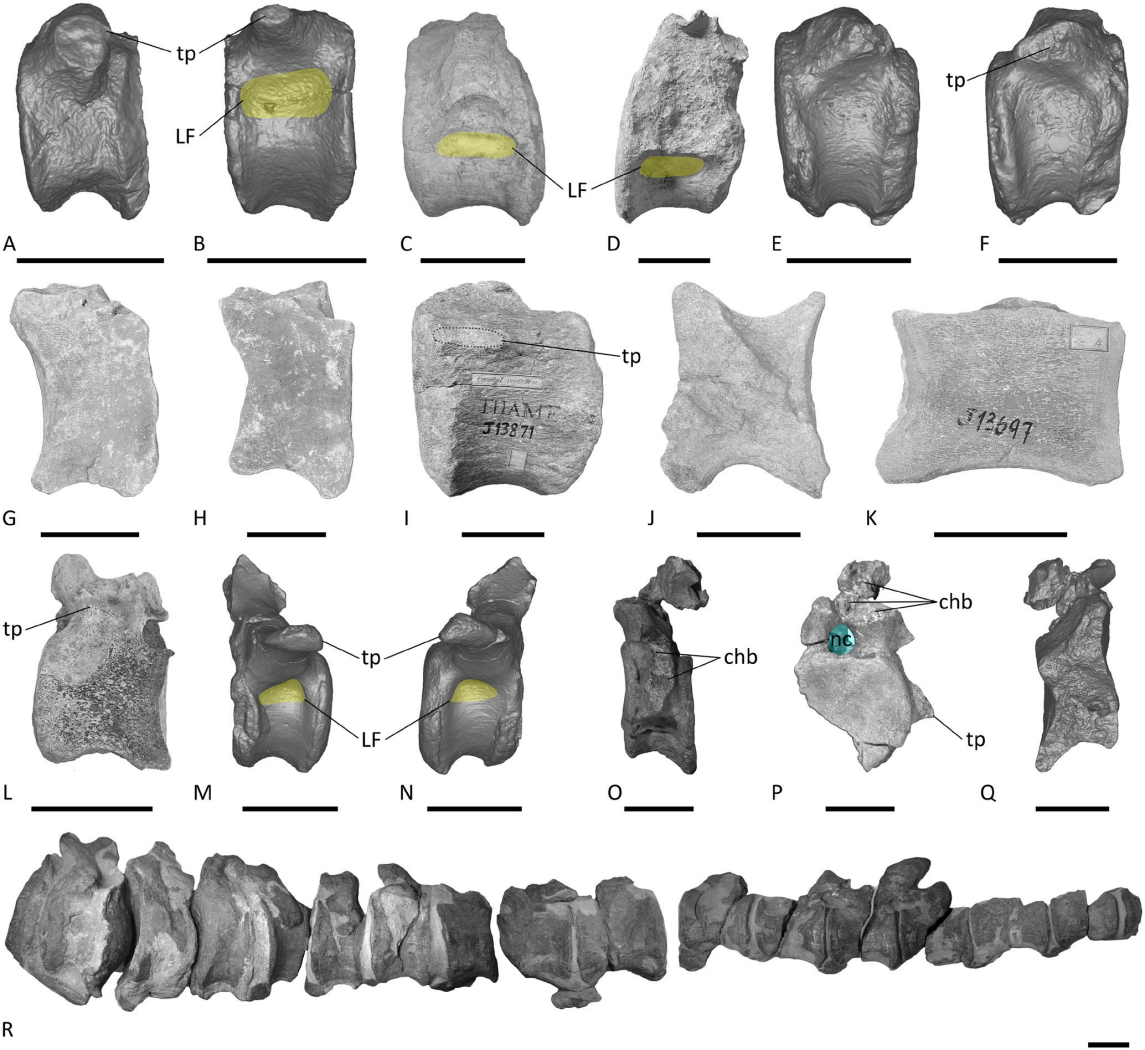
Surface scans and photographs of sauropod caudal vertebrae. (A, B) *Isanosaurus attavipachi* anterior caudal vertebra CH4‐10; (C–H) ‘*Bothriospondylus madagascariensis’* anterior caudal vertebrae (C, D) NHMUK R2599, (E, F) NHMUK R2600, (G, H) NHMUK R2612; (I–K) *Cetiosaurus* sp. (I) anterior caudal vertebra OUMNH PAL‐J.13871, (J) anterior caudal vertebra OUMNH PAL‐J.13731, (K) middle caudal vertebra OUMNH PAL‐J.13697; (L) *Nigersaurus taqueti* middle caudal vertebra MNHN.F.GDF2008; (M, N) *Narindasaurus thevenini* anterior caudal vertebra MNHN MAJ 424; (O–Q) *Savannasaurus elliottorum* anterior caudal vertebra AODF 0660; (R) *Rhoetosaurus brownei* anterior–posterior caudal vertebrae QMF1659; (A, B, E, F, M, N, Q) surface scans, (C, D, G–L, O, P, R) photographs; (A, C, E, G [mirrored], I, K–M, O, R) left lateral view, (B, D, F, H, N, Q) right lateral view, (J) dorsal view, (P) anterior view. Chb, internal chambers; LF, lateral fossa; nc, neural canal; tp, transverse process. Key: Blue highlight, neural canal; yellow highlight, centrum fossa. Scale bars = 100 mm.

There is an absence of external evidence for PSP in the caudal vertebrae of non‐eusauropod gravisaurian sauropods, including *Sanpasaurus yaoi* (McPhee et al., 2016), *Tazoudasaurus naimi* (Allain & Aquesbi, 2008) and *Vulcanodon karibaensis* (Raath, 1972). However, the ventral surfaces of the caudal centra of *Vulcanodon* each possess an anteroposteriorly elongate fossa (Raath, 1972). The right lateral surface of one anterior caudal centrum of *Isanosaurus attavipachi* possesses a fossa, but no equivalent fossa is present on the left lateral surface, or on any of the other anterior caudal centra (CH4‐10; Figure 25A,B).

#### 3.2.2 Non‐neosauropod eusauropods

In non‐neosauropod eusauropods, there is an absence of external evidence for caudal vertebral PSP in *Bagualia alba* (Gomez et al., 2021), *Barapasaurus tagorei* (SLB, personal observation), *Cetiosauriscus stewarti* (SLB, personal observation; though note that this might be a diplodocoid [e.g. Mannion & Moore, 2025]), ‘*Cetiosaurus glymptonensis*’ (CT data of a middle caudal centrum that preserves neural arch bases [OUMNH PAL‐J.13751] reveal a dense, apneumatic, spongious internal texture; Figure 2), *Cetiosaurus oxoniensis* (SLB, personal observation of OUMNH material), *Cetiosaurus* sp. (CT data [OUMNH PAL‐J.50308, anterior] and broken cross‐sections of anterior–middle caudal centra reveal a dense, apneumatic, spongious internal texture; SLB, personal observation of OUMNH material; Figures 1 and 25I–K), *Patagosaurus fariasi* (Holwerda et al., 2021: figs 19–30), *Rhoetosaurus brownei* (Figure 25R) and *Spinophorosaurus nigerensis* (Vidal et al., 2020: fig. S4); the mamenchisaurids *Analong chuanjieensis* (Sekiya, 2011: figs 27–35; Ren et al., 2020), *Chuanjiesaurus anaensis* (Sekiya, 2011: figs 21, 23–26), *Mamenchisaurus hochuanensis* (Young & Zhao, 1972), *Mamenchisaurus youngi* (Pi et al., 1996; Ouyang & Ye, 2002: figs 30–32, plates XIV–XV), *Omeisaurus jiaoi* (Jiang et al., 2011: figs 3 and 4), *Omeisaurus puxiani* (Tan et al., 2020: fig. 5) and *Wamweracaudia keranjei* (CT data of anterior–middle caudal vertebrae reveal a dense, apneumatic, spongious internal texture; Figures 3 and 4); and the turiasaurs *Losillasaurus giganteus* (Royo‐Torres et al., 2021), *Mierasaurus bobyoungi* (Royo‐Torres, Upchurch, et al., 2017), and *Moabosaurus utahensis* (Britt et al., 2017: figs 24–26).

Possible external evidence for caudal vertebral PSP occurs in one anterior caudal centrum of ‘*Bothriospondylus madagascariensis*’, which is characterised by a lateral fossa (NHMUK R2599; Figure 25C,D; Mannion, 2010). However, other anterior and middle caudal centra of this taxon lack a lateral fossa (NHMUK R2600; Figure 25E,F), and a broken cross‐section reveals a dense, apneumatic, spongious internal texture (NHMUK R2612; Figure 25G,H). The mamenchisaurids *Bellusaurus sui* (Ca1 only; Mo, 2013: fig. 33; though see some analyses for a neosauropod placement [e.g. Gomez et al., 2021]) and *Omeisaurus tianfuensis* (Ca3 only; He et al., 1988: fig. 34), as well as the turiasaur *Narindasaurus thevenini* (MNHN MAJ 424; Figure 25M,N), each possess a lateral fossa in a single anterior caudal centrum. The neural arches of Ca1–Ca4 of *Mamenchisaurus sanjiangensis* possess evidence for PSP (i.e. subfossae within fossae and accessory fossae), whereas the centra lack such features (Dai et al., 2025).

#### 3.2.3 Diplodocoid neosauropods

Within Diplodocoidea, external evidence for caudal vertebral pneumaticity is absent in *Haplocanthosaurus delfsi* (CMNH 10380; PDM and PU, personal observation), *Haplocanthosaurus priscus* (CM 572; Hatcher, 1903: plate III) and *Haplocanthosaurus* sp. (MWC 8028; Foster & Wedel, 2014). Conversely, Ca1 of *Haplocanthosaurus* sp. (FHPR 1106; PU, personal observation) and an immature specimen of *H*. *priscus* (CM 879; Wedel, 2009) each possess a LPF. CT data of Ca3 of *Haplocanthosaurus* sp. (MWC 8028) reveals a dense, apneumatic, spongious internal texture (Wedel et al., 2021: fig. 1).

**FIGURE 26:**
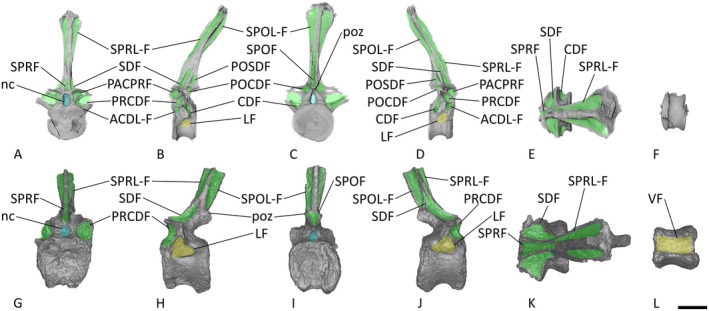
3D rendering and surface scans of rebbachisaurid anterior caudal vertebrae. (A–F) Rebbachisauridae indet. MDPA‐Pv 007 3D rendering, (G–L) ‘*Rebbachisaurus tamesnensis’*, MNHN unnumbered surface scan; (A, G) anterior view, (B, H) left lateral view, (C, I) posterior view, (D, J) right lateral view, (E, K) dorsal view, (F, L) ventral view. ACDL‐F, anterior centrodiapophyseal lamina‐fossa; CDF, centrodiapophyseal fossa; LF, lateral fossa; nc, neural canal; PACPRF, parapophyseal centroprezygapophyseal fossa; POCDF, postzygapophyseal centrodiapophyseal fossa; poz, postzygapophysis; POSDF, postzygapophyseal spinodiapophyseal fossa; PRCDF, prezygapophyseal centrodiapophyseal fossa; prz, prezygapophysis; SDF, spinodiapophyseal fossa; SPOF, spinopostzygapophyseal fossa; SPOL‐F, spinopostzygapophyseal lamina‐fossa; SPRF, spinoprezygapophyseal fossa; SPRL‐F, spinoprezygapophyseal lamina‐fossa; tp, transverse process; VF, ventral fossa. Key: Blue highlight, neural canal; green highlight, neural spine fossa; yellow highlight, centrum fossa. Scale bar = 100 mm.

**FIGURE 27:**
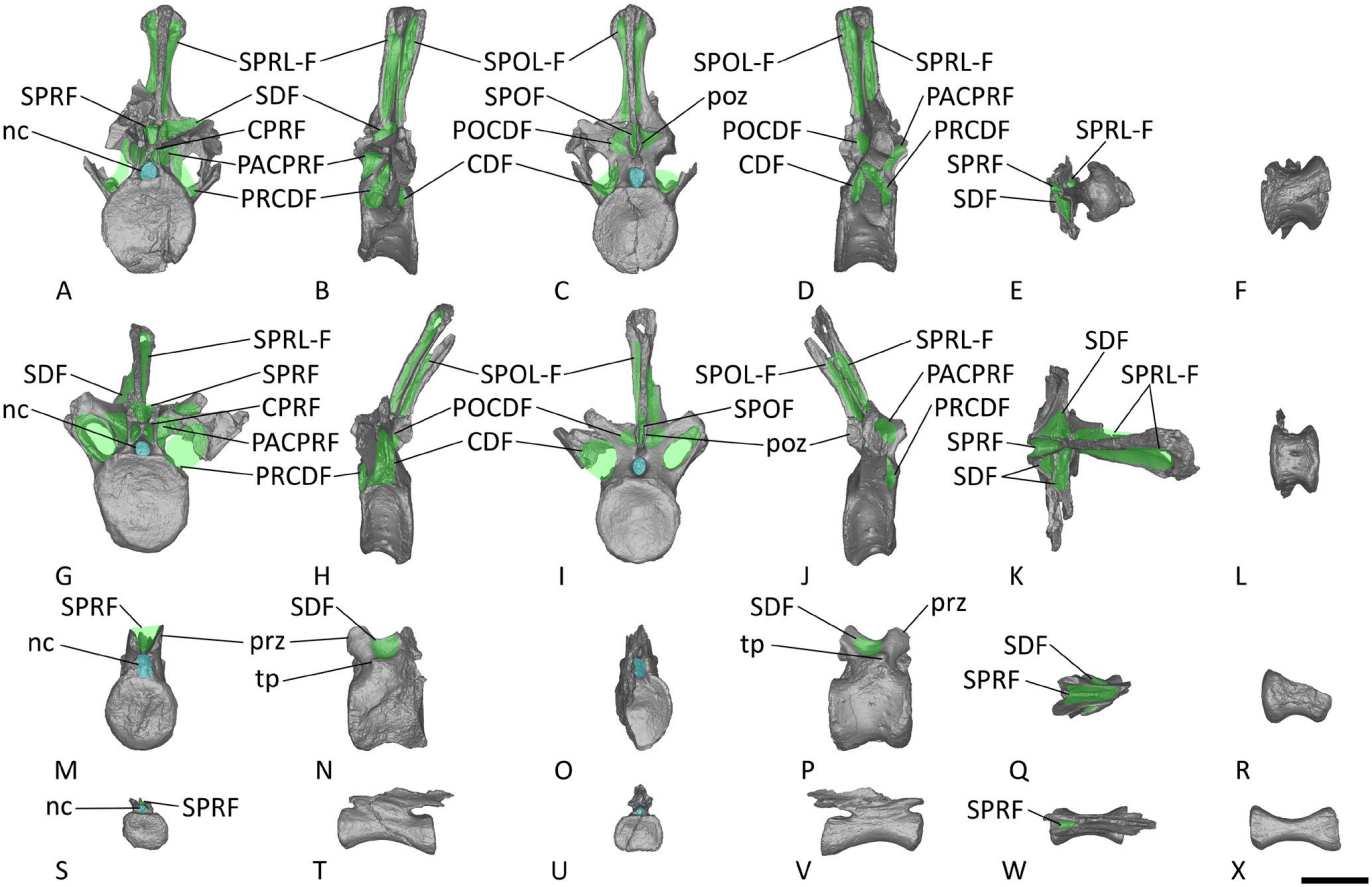
Surface scans of *Nigersaurus taqueti* caudal vertebrae. (A–F) anterior caudal vertebra MNHN unnumbered, (G–L) anterior caudal vertebra MNHN unnumbered, (M–R) middle caudal vertebra MNHN.F.GDF2008, (S–X) posterior caudal vertebra MNHN.F.GDF673; (A, G, M, S) anterior view, (B, H, N, T) left lateral view, (C, I, O, U) posterior view, (D, J, P, V) right lateral view, (E, K, Q, W) dorsal view, (F, L, R, X) ventral view. CDF, centrodiapophyseal fossa; CPRF, centroprezygapophyseal fossa; nc, neural canal; PACPRF, parapophyseal centroprezygapophyseal fossa; POCDF, postzygapophyseal centrodiapophyseal fossa; poz, postzygapophysis; PRCDF, prezygapophyseal centrodiapophyseal fossa; prz, prezygapophysis; SDF, spinodiapophyseal fossa; SPOF, spinopostzygapophyseal fossa; SPOL‐F, spinopostzygapophyseal lamina‐fossa; SPRF, spinoprezygapophyseal fossa; SPRL‐F, spinoprezygapophyseal lamina‐fossa; tp, transverse process. Key: Blue highlight, neural canal; green highlight, neural spine fossa. Scale bar = 100 mm.

In rebbachisaurids, external evidence for PSP is absent in the caudal vertebrae of *Cathartesaura anaerobica* (Gallina & Apesteguía, 2005) and *Tataouinea hannibalis* (*contra* Fanti et al., 2015: compare supplementary file S2 with fig. 8 wherein the ‘pleurocoel’ is actually collapsed and deformed bone). External evidence for PSP occurs in the caudal centra of *Astigmasaura genuflexa* (Bellardini et al., 2025), *Limaysaurus tessonei* (Calvo & Salgado, 1995: fig. 10) and *Sidersaura marae* (Lerzo et al., 2025), as well as an indeterminate rebbachisaurid (UNPSJB‐PV 580, UNPSJB‐PV 1004–1005; Ibiricu et al., 2012); the caudal centra and neural arch of *Comahuesaurus windhauseni* (Carballido, Salgado, et al., 2012), an indeterminate rebbachisaurid (MDPA‐Pv 007; Windholz et al., 2024; Figure 26A–F) and an indeterminate rebbachisaurine (‘*Rebbachisaurus tamesnensis*’, MNHN unnumbered; Mannion & Moore, 2025; Figure 26G–L); and the neural arch (but not the centrum) of *Demandasaurus darwini* (Torcida Fernández‐Baldor et al., 2011). Anterior caudal vertebrae of MNHN material probably assignable to *Nigersaurus taqueti* possess external evidence for PSP in the centra and neural arches, whereas the middle–posterior elements lack such evidence (Figure 27), and the broken cross‐section of a middle caudal centrum (MNHN.F.GDF2008) reveals a dense, apneumatic, spongious internal texture (Figure 25L).

**FIGURE 28:**
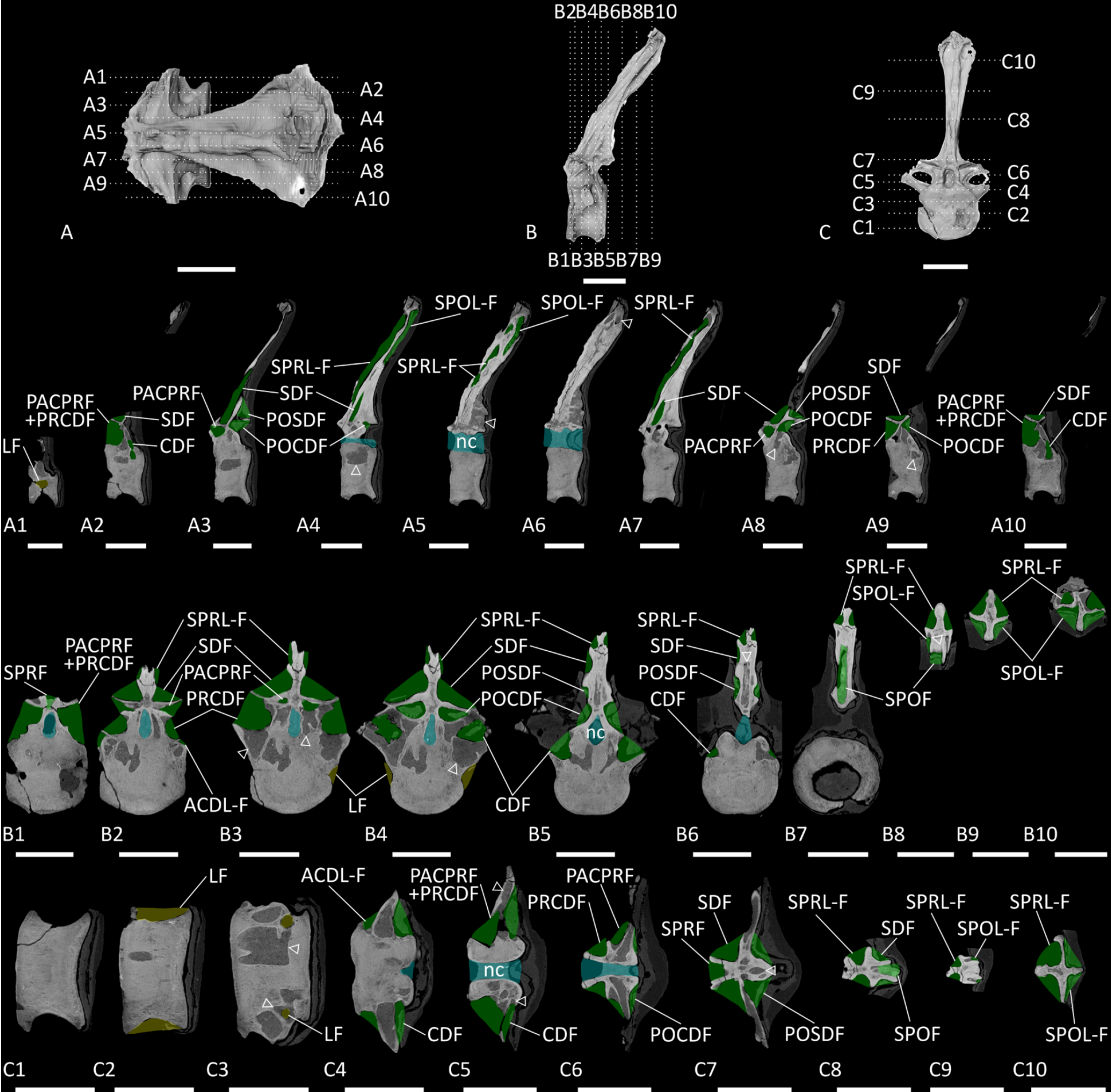
CT scan of Rebbachisauridae indet. anterior caudal vertebra MDPA‐Pv 007. (A–C) 3D rendering (A) dorsal view, (B) left lateral view, (C) anterior view; (A1–A10) CT scan slices in parasagittal cross‐section, (B1–B10) CT scan slices in transverse cross‐section, (C1–C10) CT scan slices in horizontal cross‐section. ACDL‐F, anterior centrodiapophyseal lamina‐fossa; CDF, centrodiapophyseal fossa; LF, lateral fossa; nc, neural canal; PACPRF, parapophyseal centroprezygapophyseal fossa; POCDF, postzygapophyseal centrodiapophyseal fossa; poz, postzygapophysis; POSDF, postzygapophyseal spinodiapophyseal fossa; PRCDF, prezygapophyseal centrodiapophyseal fossa; prz, prezygapophysis; SDF, spinodiapophyseal fossa; SPOF, spinopostzygapophyseal fossa; SPOL‐F, spinopostzygapophyseal lamina‐fossa; SPRF, spinoprezygapophyseal fossa; SPRL‐F, spinoprezygapophyseal lamina‐fossa; tp, transverse process. Key: Blue highlight, neural canal; green highlight, neural spine fossa; yellow highlight, centrum fossa; white arrow, pneumatic bone texture. Scale bars = 100 mm.

Internal evidence for PSP occurs in an anterior caudal vertebra of an indeterminate rebbachisaurid (Ca1 or Ca2, MDPA‐Pv 007; Windholz et al., 2024; Figure 28) that pneumatises the centrum and neural arch via external fossae, as well as the floor and roof of the neural canal. By contrast with the shallow lateral fossa on the centrum, the external fossae on the neural arch and spine of MDPA‐Pv 007 are extensive and deep. The internal pneumatic chambers are camerate (Table 1): they are large, generally separate and surrounded by thick, compact, apneumatic bone.

The dicraeosaurids *Amargasaurus cazaui* (Salgado & Bonaparte, 1991), *Dicraeosaurus hansemanni* (SLB, personal observation), *Dicraeosaurus sattleri* (Figure 5) and *Suuwassea emilieae* (Harris, 2006) lack external evidence for caudal vertebral PSP. CT data of Ca3 of *D*. *sattleri* reveals a dense, apneumatic, spongious internal texture (Figure 5).

**FIGURE 29:**
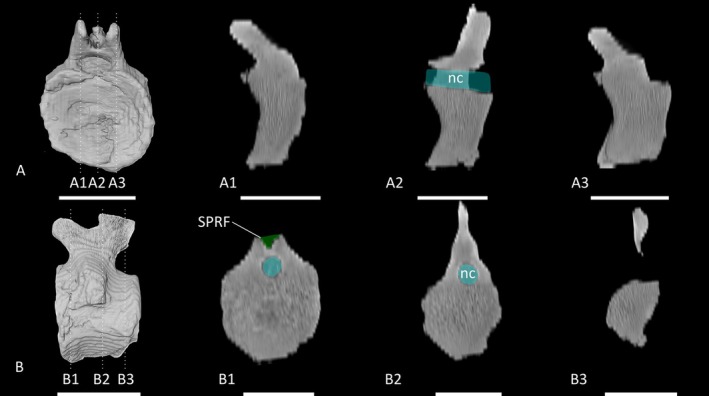
CT scan of *Pilmatueia faundezi* middle caudal vertebra MLL‐Pv‐016. (A, B) 3D rendering (A) anterior view, (B) left lateral view; (A1–A3) CT scan slices in parasagittal cross‐section, (B1–B3) CT scan slices in transverse cross‐section. nc, neural canal; SPRF, spinoprezygapophyseal fossa. Key: Blue highlight, neural canal; green highlight, neural spine fossa. Scale bars = 100 mm.

In diplodocids, external evidence for caudal vertebral PSP is absent in an indeterminate apatosaurine (NSMT‐PV 20375, formerly referred to *Apatosaurus ajax*; Upchurch et al., 2004; Tschopp et al., 2015) and *Pilmatueia faundezi* (Windholz et al., 2020; Figure 29; note though that this species is typically regarded as a dicraeosaurid, but see Mannion & Moore, 2025). CT data of a middle caudal vertebra of *Pilmatueia* reveals a dense, apneumatic, spongious internal texture (Windholz et al., 2020; Figure 29). However, external evidence for PSP occurs in the caudal centra of an indeterminate diplodocid (FMNH P25112 [formerly FMNH 7163]), previously assigned to *Brontosaurus excelsus* (Riggs, 1903; Tschopp et al., 2015; Wedel & Taylor, 2013), as well as *Apatosaurus louisae* (CM 3018; Wedel & Taylor, 2013). A more complex pattern occurs in *Brontosaurus excelsus* (YPM 1980; Tschopp et al., 2015), wherein the anterior 12 caudal centra each possess several nutrient foramina, and only the left side of Ca9 possess a LPF (Wedel & Taylor, 2013). Wedel and Taylor (2013) also described a LPF on the right side only of Ca13 of YPM 1980, but new preparation of the element reveals that the LPF was likely enhanced with plaster by the original preparators, and is instead an artefactual feature (Taylor, 2023).

Within Diplodocinae, external evidence for caudal vertebral PSP occurs in the anterior caudal centra of *Ardetosaurus viator* (van der Linden et al., 2024; note though that the anteriormost caudal neural arches are not preserved); and in the caudal centra and neural arches of Ca1–Ca19 of *Barosaurus lentus* (YPM 429; Marsh, 1890: fig. 2; Lull, 1919), Ca1–Ca18 of *Diplodocus carnegii* (CM 84; Britt, 1993), anterior and middle caudal vertebrae of *Leinkupal laticauda* (Gallina et al., 2014), anterior caudal vertebrae of *Supersaurus vivianae* (Lovelace et al., 2007; SLB, personal observation of photographs) and the ~anterior 15–20 caudal vertebrae of *Tornieria* (see above; Janensch, 1947: Abb. 9; Wedel & Taylor, 2013; Figure 6). Furthermore, in *Barosaurus*, *Diplodocus* and *Tornieria*, the anterior caudal neural arches are heavily excavated by fossae. CT data reveals that the anterior–posterior caudal vertebrae of *Tornieria* are pneumatised (see above; Figures 7, 8, 9, 10, 11, 12, 13). The internal pneumatic texture of *Tornieria* does not strictly align with the current definitions of camerate, polycamerate or camellate (Table 1). Instead, the anterior and proximal‐middle caudal vertebrae possess smaller chambers within larger chambers that communicate with each other, and the distal‐middle and posterior caudal vertebrae possess separate chambers that differ in size and do not bifurcate. Furthermore, these chambers are each separated by a thin layer of compact bone.

**FIGURE 30:**
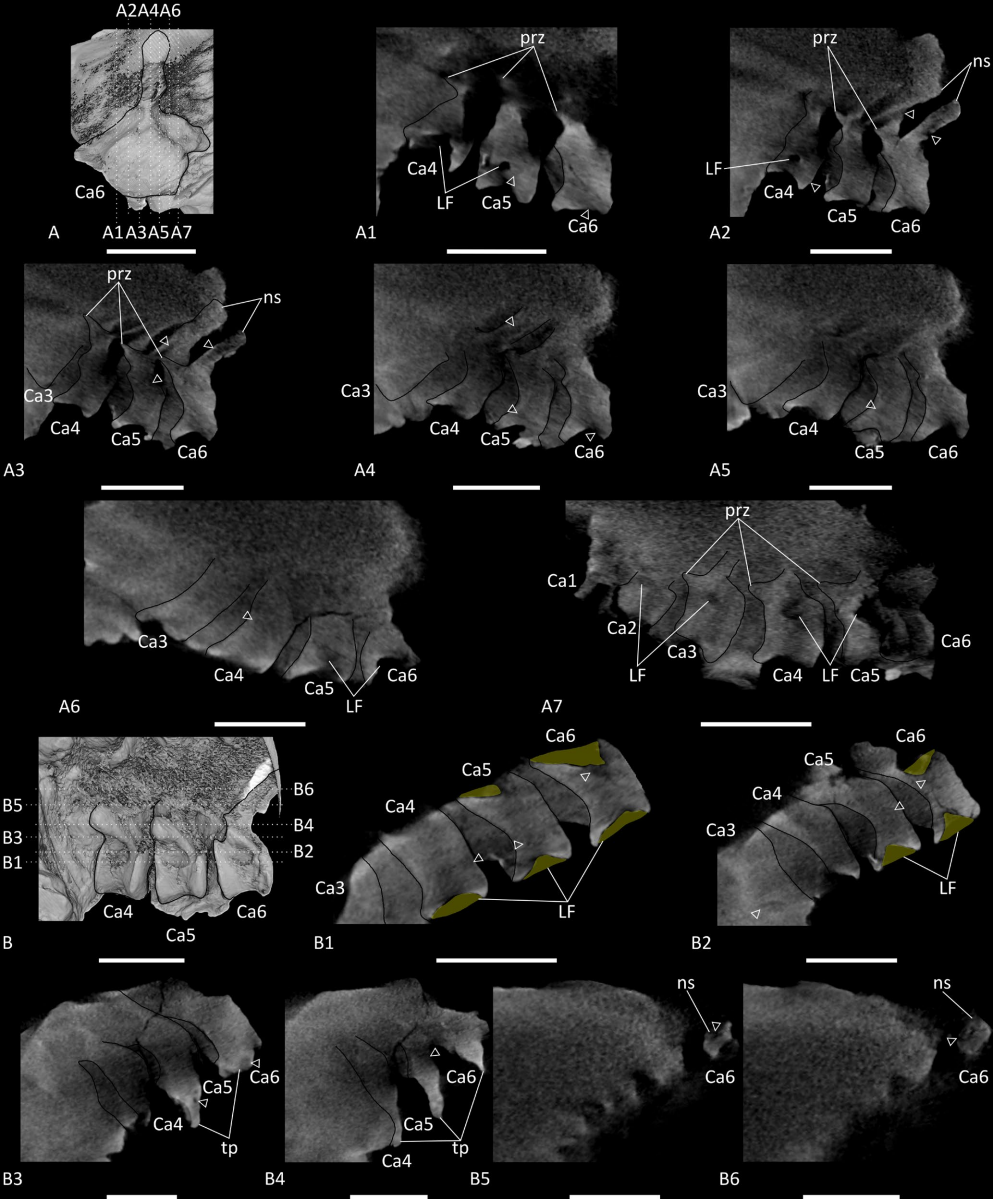
CT scan of *Barosaurus* sp. DINO 2921 anterior caudal vertebrae 1–6. (A, B) 3D rendering (A) posterior view, (B) left lateral view; (A1–A7) CT scan slices in parasagittal cross‐section, (B1–B6) CT scan slices in horizontal cross‐section. Ca, caudal vertebra; LF, lateral fossa; ns, neural spine; prz, prezygapophysis; tp, transverse process. Key: Yellow highlight, centrum fossa; white arrow, pneumatic bone texture. Scale bars = 100 mm.

**FIGURE 31:**
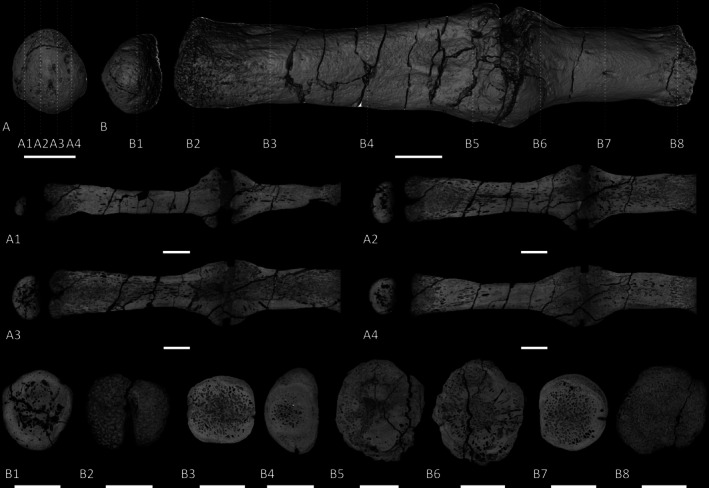
CT scan of *Barosaurus* sp. NAMAL‐106 terminal two caudal vertebrae CaA and CaB. (A, B) 3D rendering (A) CaB posterior view, (B) CaA left lateral view and CaB right lateral view; (A1–A4) CT scan slices in parasagittal cross‐section, (B1–B8) CT scan slices in transverse cross‐section. Scale bars = 10 mm.

In the anterior caudal vertebrae of an immature specimen referred to *Barosaurus* sp. (DINO 2921), Ca1 lacks a LPF, but such a structure is present in Ca4–Ca6 (note Ca2–Ca3 are externally inaccessible; Melstrom et al., 2016). CT data reveals that Ca2–Ca6 each possess a LPF that communicates with internal pneumatic chambers (Figure 30A6–A7,B1–B2). Therefore, DINO 2921 appears to possess internal evidence for PSP (Figure 30), despite not being described as such by Melstrom et al. (2016). However, we are tentative in this assertion because of the low contrast resolution of the CT scan slices (particularly in Ca1–Ca3). Nevertheless, internal pneumatic chambers also appear to be present in the transverse processes of Ca5 and Ca6 (Figure 30B3–B4), and the neural arches and spines of Ca4–Ca6 (Figure 30A2–A4,B5–B6) (note that the transverse processes and neural arches and spines of more anterior elements cannot be assessed). Presumably, the transverse processes are pneumatised by chambers that extend through the centrum. The neural arches and spines appear to be pneumatised via external fossae, but which fossae these represent cannot be determined. Given the phylogenetically nested position of *Barosaurus* within Diplodocinae (e.g. Tschopp et al., 2015), it would not be surprising for its anterior caudal vertebrae to be pneumatic. Furthermore, the development of PSP has been found to increase with ontogeny (e.g. Schwarz et al., 2007; Wedel, 2003a). Therefore, the immature status of DINO 2921 implies that adult *Barosaurus* specimens would display a comparative increase in PSP. CT data of the distalmost two caudal vertebrae of another specimen referred to *Barosaurus* sp. (NAMAL‐106; van der Linden et al., 2026) reveal an apneumatic, spongious internal texture (Figure 31).

#### 3.2.4 Non‐titanosaurian macronarian neosauropods

External evidence for caudal vertebral PSP is absent in all non‐titanosauriform macronarians. This includes *Aragosaurus ischiaticus* (Royo‐Torres et al., 2014), *Dashanpusaurus dongi* (Ren et al., 2022) and *Galveosaurus herreroi* (Pérez‐Pueyo et al., 2019); the camarasauromorphs *Europasaurus holgeri* (immature and adult individuals; Carballido & Sander, 2014) and *Oceanotitan dantasi* (Mocho, Royo‐Torres, & Ortega, 2019: figs 2 and 3); and the camarasaurids *Camarasaurus grandis* (GMNH 101; McIntosh et al., 1996), *Camarasaurus lentus* (CM 11338, immature individual; Gilmore, 1925: Plate XIV; Carpenter et al., 2026), *Camarasaurus lewisi* (BYU 9047; Britt, 1993), *Camarasaurus supremus* (AMNH 5760–5761; Osborn & Mook, 1921) and *Lourinhasaurus alenquerensis* (Mocho et al., 2014). External evidence for caudal vertebral PSP is relatively rare in non‐somphospondylan titanosauriforms, being absent in the brachiosaurids *Brachiosaurus* sp. (SMA 0009, immature individual; Schwarz et al., 2007; Carballido, Marpmann, et al., 2012), *Brachiosaurus altithorax* (Riggs, 1904), *Cedarosaurus weiskopfae* (note that we follow Tidwell et al., 1999 in regarding the lateral depression to not be a true LPF because it lacks defined rims), *Lusotitan atalaiensis* (Mannion et al., 2013), *Soriatitan golmayensis* (note that we regard the lateral depression described by Royo‐Torres, Fuentes, et al., 2017 to not be a true LPF because it lacks defined rims) and *Vouivria damparisensis* (Mannion et al., 2017). Conversely, the anterior–middle caudal centra of the brachiosaurid *Venenosaurus dicrocei* possess a true LPF with defined rims (Tidwell et al., 2001: figs 11.3–11.4).

External evidence for caudal vertebral PSP occurs in the centra of the brachiosaurid *Giraffatitan brancai*, which possesses a complex pattern in Ca2–Ca24 of one specimen (MB.R.5000), with several pneumatic hiatuses in this series (Wedel & Taylor, 2013: figs 4, 5 and 8; Figure 14), whereas another two individuals (MB.R.2921, Ca1–Ca18; and MB.R.3736, Ca1–Ca31) lack a LPF in all elements except for Ca2 (Wedel & Taylor, 2013: fig. 7; Figure 15). CT data from anterior–middle caudal vertebrae of MB.R.5000 reveals pneumatic internal texture in the centra and neural arches (Figures 16, 17, 18, 19, 20). There are fewer external fossae in *Giraffatitan* than in *Tornieria* and Rebbachisauridae indet. (MDPA‐Pv 007), and they are not as extensive or as deep in the former as they are in the latter two taxa. Furthermore, the internal bone texture of *Giraffatitan* does not align with that of *Tornieria* or Rebbachisauridae indet. (MDPA‐Pv 007), nor with current internal pneumaticity definitions (Table 1).

Most non‐titanosaurian somphospondylans lack external evidence for caudal vertebral PSP, including *Astrophocaudia slaughteri* (D'Emic, 2013), *Chubutisaurus insignis* (Carballido et al., 2011; SLB, personal observation of photographs; although Poropat et al., 2020 noted the presence of a LPF in *Chubutisaurus*, we consider the lateral surfaces of the anterior caudal centra to possess small nutrient foramina, and not a true LPF), *Dongbeititan dongi* (SLB, personal observation of photographs), *Europatitan eastwoodi* (Torcida Fernández‐Baldor et al., 2017: figs 10 and 11), *Garumbatitan morellensis* (*contra* Mocho, Escaso, Gasulla, et al., 2024: see figs 5 and 6 wherein the fossa is a shallow lateral concavity and not a true LPF), *Huabeisaurus allocotus* (D'Emic et al., 2013), *Ligabuesaurus leanzai* (Bellardini et al., 2022), the euhelopodid *Phuwiangosaurus sirindhornae* (subadult and adult specimens; Suteethorn et al., 2009, 2010), *Ruixinia zhangi* (Mo et al., 2023), *Sonidosaurus saihangaobiensis* (Xu et al., 2006: fig. 1), *Tastavinsaurus sanzi* (Canudo et al., 2008: figs 7 and 8) and *Yunmenglong ruyangensis* (Lü et al., 2013). External evidence for caudal vertebral PSP, in the form of a LPF, occurs in the centra of Ca2 and Ca4 of the probable somphospondylan *Padillasaurus leivaensis* (note that a LPF is absent in Ca1, Ca3, Ca5–Ca8; though see Carballido et al., 2015 for a brachiosaurid identification). It is also present in the anterior caudal neural arches of the probable euhelopodid somphospondylan *Tambatitanis amicitiae* (note that the small oval depression on the right side of the centrum of Ca2 does not appear to be pneumatic, *contra* Saegusa & Ikeda, 2014: figs 8 and 10). In *Tambatitanis*, CT data of Ca1–Ca6 reveals pneumatised neural arches in Ca1–Ca5, whereas the centra of Ca1–Ca6 and the neural arch of Ca6 are apneumatic (Saegusa & Ikeda, 2014: figs 9–14). The external fossae on Ca1–Ca5 of *Tambatitanis* deeply excavate the neural arches and communicate with internal pneumatic chambers that are separated from each other by compact bone (Saegusa & Ikeda, 2014: figs 9–13).

**FIGURE 32:**
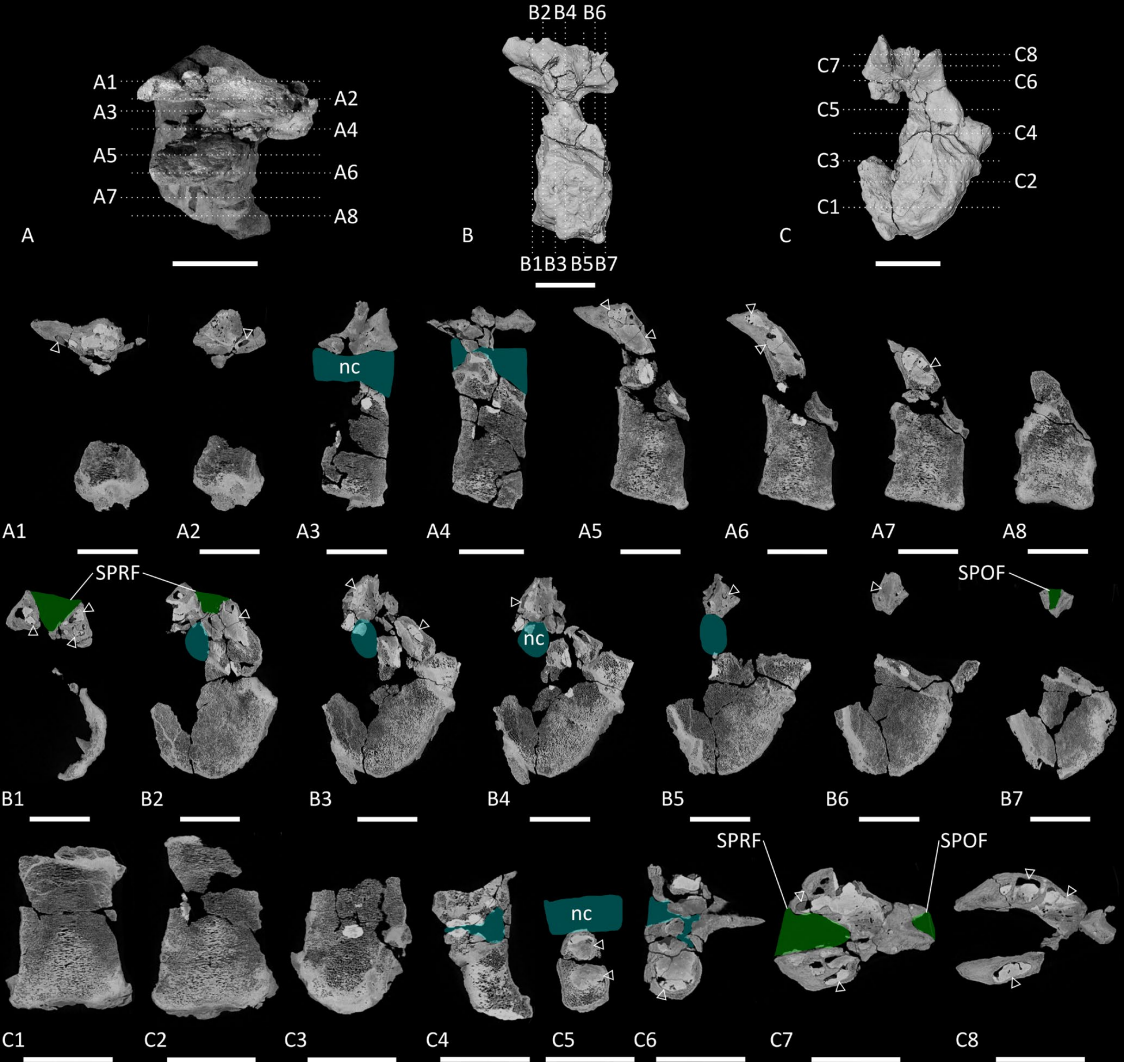
CT scan of *Wintonotitan wattsi* anterior caudal vertebra ‘D’ QMF7292. (A) photograph dorsal view, (B) 3D rendering left lateral view, (C) 3D rendering anterior view; (A1–A8) CT scan slices in parasagittal cross‐section, (B1–B7) CT scan slices in transverse cross‐section, (C1–C8) CT scan slices in horizontal cross‐section. Nc, neural canal; SPOF, spinopostzygapophyseal fossa; SPRF, spinoprezygapophyseal fossa. Key: Blue highlight, neural canal; green highlight, neural spine fossa; white arrow, pneumatic bone texture. Scale bars = 100 mm.

**FIGURE 33:**
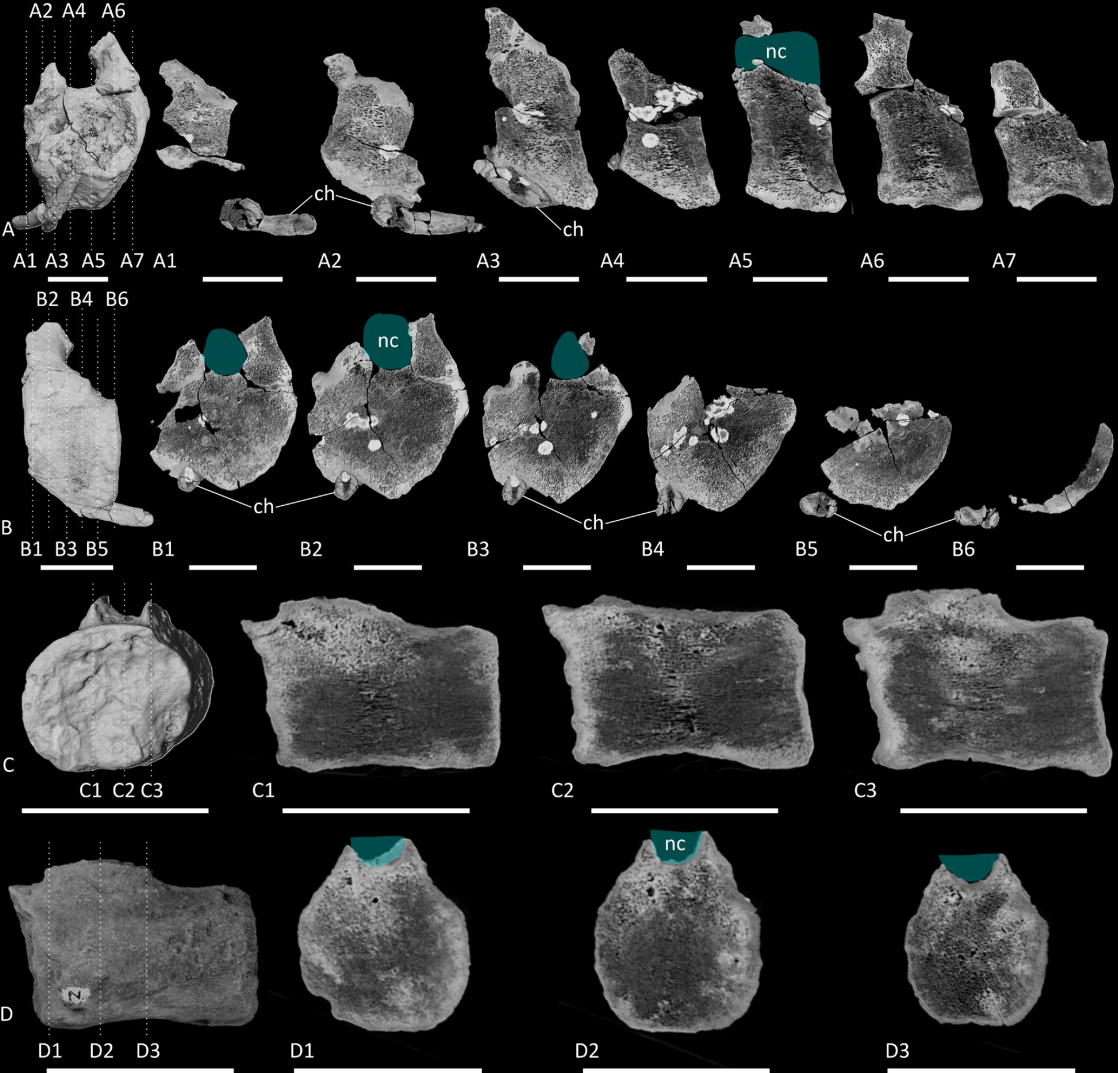
CT scan of *Wintonotitan wattsi* anterior and middle caudal vertebrae QMF7292. (A, C) 3D rendering anterior view, (B) 3D rendering left lateral view, (D) photograph left lateral view; (A, B) anterior caudal vertebra ‘E’, (C, D) middle caudal vertebra ‘N’; (A1–A7, C1–C3) CT scan slices in parasagittal cross‐section, (B1–B6, D1–D3) CT scan slices in transverse cross‐section. Ch, chevron; nc, neural canal. Key: Blue highlight, neural canal. Scale bars = 100 mm.

**FIGURE 34:**
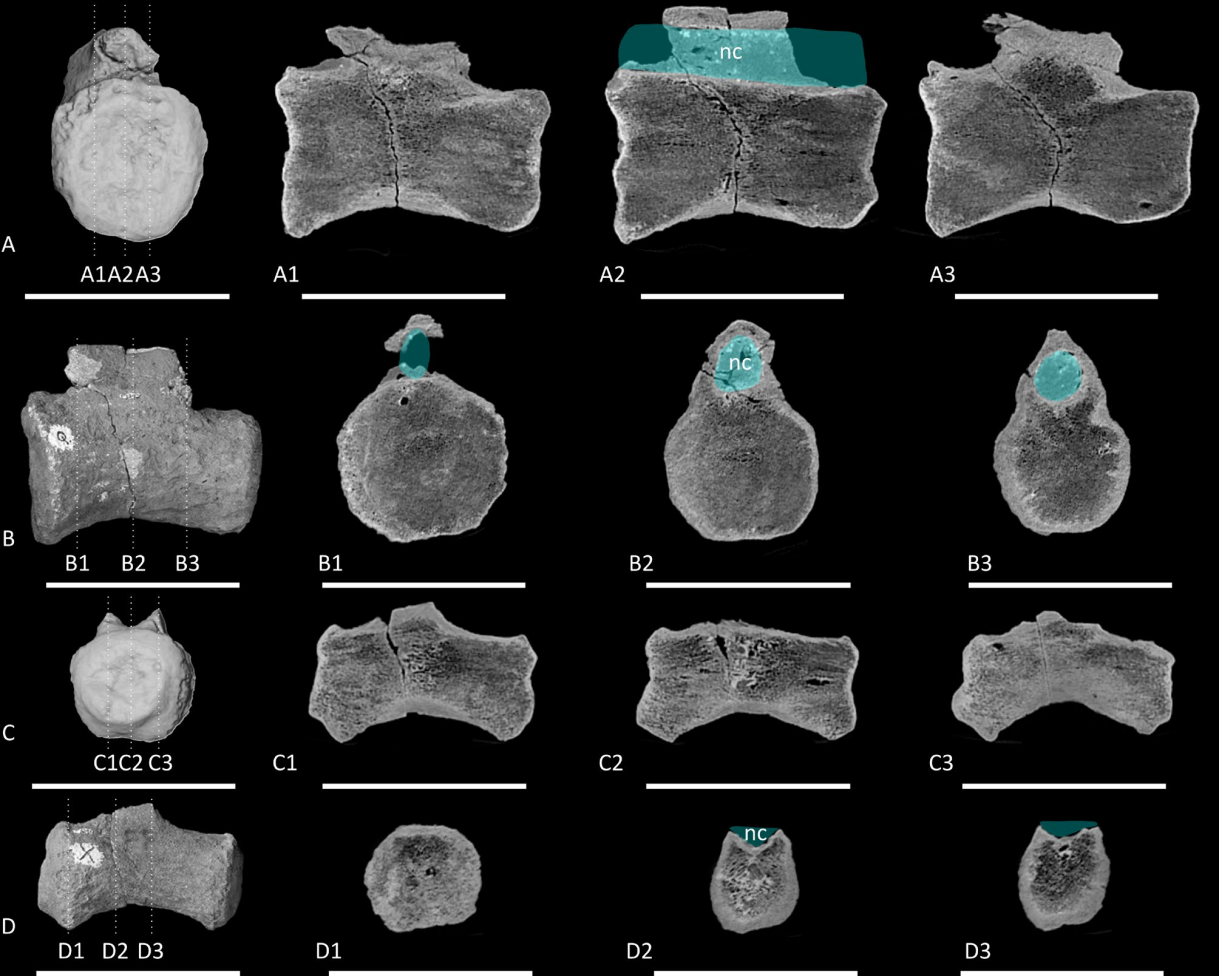
CT scan of *Wintonotitan wattsi* posterior caudal vertebrae QMF7292. (A, C) 3D rendering anterior view, (B, D) photograph left lateral view; (A, B) posterior caudal vertebra ‘Q’, (C, D) posterior caudal vertebra ‘X’; (A1–A3, C1–C3) CT scan slices in parasagittal cross‐section, (B1–B3, D1–D3) CT scan slices in transverse cross‐section. Nc, neural canal. Key: Blue highlight, neural canal. Scale bars = 100 mm.

Whereas the anterior caudal centra of the diamantinasaurian somphospondylan *Savannasaurus elliottorum* possess small nutrient foramina, they are unlikely to be pneumatic in origin because broken surfaces of the centra reveal a dense, spongious internal texture (Figure 25Q). Conversely, broken surfaces of one anterior caudal neural arch of *Savannasaurus* reveals internal pneumatic chambers (AODF 0660; Poropat et al., 2016, 2020; Figure 25O,P). External evidence for PSP is absent in the caudal vertebrae of the diamantinasaurians *Diamantinasaurus matildae* (Poropat et al., 2023; SLB, personal observation), *Wintonotitan wattsi* (Poropat et al., 2015; SLB, personal observation) and indeterminate specimens of the diamantinasaurian clade (AODF 0032, AODF 0590, AODF 2296; Beeston et al., 2024). However, CT data of *Wintonotitan* reveals a pneumatised neural arch in the only anterior caudal vertebra to preserve this region (caudal vertebra ‘D’ of Poropat et al., 2015: fig. 3; Hocknull et al., 2021; Figure 32). The internal pneumatic texture of the lower neural arch, zygapophyses and neural spine of anterior caudal vertebra ‘D’ communicates with the external fossae (i.e. the SPRF and the SPOF), and potentially the roof of the neural canal. The internal pneumatic chambers are not camellate (*contra* Hocknull et al., 2021); instead, they are large, separate chambers that do not bifurcate, and they are more closely aligned with the definition of camerae (Table 1). However, they differ to the camerate chambers described above for Rebbachisauridae indet. (MDPA‐Pv 007) (compare Figures 28 and 32). CT data of *Wintonotitan* further reveal that several nutrient foramina on the lateral and ventral surfaces of the anterior–posterior caudal centra extend a considerable distance inside the apneumatic centra (e.g. see Figure 33D3), and, except for caudal vertebra ‘D’, all other anterior–posterior caudal centra and neural arch bases of *Wintonotitan* possess dense, apneumatic spongious internal texture (Figures 33 and 34). Finally, a chevron attached to caudal vertebra ‘E’ of *Wintonotitan* possesses densely packed apneumatic spongious texture (Figure 33A,B).

#### 3.2.5 Non‐saltasaurine titanosaurs

Because of the uncertain phylogenetic relationships of many titanosaurian OTUs (e.g. see Díez Díaz et al., 2025), with the exception of Saltasaurinae, we comment upon all titanosaurian taxa together, rather than in their separate, sometimes unstable clades. Some of these species might also lie outside of Titanosauria, with contrasting placements in competing phylogenetic hypotheses (e.g. see Díez Díaz et al., 2025). External evidence for PSP is absent in the caudal vertebrae of *Alamosaurus sanjuanensis* (Gilmore, 1946: plates 5 and 8; note that we regard the putatively autapomorphic [sensu Wilson, 2002; Tykoski & Fiorillo, 2017: see fig. 10] foramina that pierce the lateral surface of the centrum of Ca1 to be nutrient foramina), *Ampelosaurus atacis* (Le Loeuff, 2005: fig. 4.8), *Arrudatitan maximus* (SLB, personal observation of photographs), *Atsinganosaurus velauciensis* (Díez Díaz et al., 2018: figs 7 and 8; however, those authors described one anterior centrum as possessing camellate internal texture), *Baotianmansaurus henanensis* (Zhang et al., 2009), *Bonitasaura salgadoi* (Gallina & Apesteguía, 2015), *Epachthosaurus sciuttoi* (SLB, personal observation of photographs), *Futalognkosaurus dukei* (SLB, personal observation of photographs), *Gondwanatitan faustoi* (Kellner & Azevedo, 1999; SLB, personal observation of photographs), *Isisaurus colberti* (SLB, personal observation), *Lirainosaurus astibiae* (Díez Díaz et al., 2013: figs 5 and 6), *Lohuecotitan pandafilandi* (Díez Díaz et al., 2016: figs 3 and 5), *Magyarosaurus dacus* (Díez Díaz et al., 2025: fig. 24), *Menucocelsior arriagadai* (note Ca1 centrum lateral surfaces possess large nutrient foramina; Aranciaga Rolando et al., 2022: fig. 2), *Mnyamawamtuka moyowamkia* (Gorscak & O'Connor, 2019), *Muyelensaurus pecheni* (Pérez Moreno et al., 2026: fig. 10; SLB, personal observation of photographs), *Normanniasaurus genceyi* (Le Loeuff et al., 2013; SLB, personal observation of photographs), *Notocolossus gonzalezparejasi* (González Riga et al., 2016), *Nullotitan glaciaris* (note that the lateral surfaces of anterior caudal centra possess small nutrient foramina; Novas et al., 2019: figs 16 and 17), *Opisthocoelicaudia skarzynskii* (Borsuk‐Białynicka, 1977), *Overosaurus paradasorum* (Coria et al., 2013: fig. 6), *Paludititan nalatzensis* (Csiki et al., 2010: fig. 3), *Punatitan coughlini* (Hechenleitner et al., 2020: fig. 2), *Quetecsaurus rusconii* (González Riga & Ortiz David, 2014: fig. 8), *Qunkasaura pintiquiniestra* (SLB, personal observation of 3D models from Mocho, Escaso, Marcos‐Fernández, et al., 2024: supplementary note 5), *Rapetosaurus krausei* (immature individual; Curry Rogers, 2009), *Rukwatitan bisepultus* (Gorscak et al., 2014), *Uberabatitan ribeiroi* (Silva Junior et al., 2019), *Volgatitan simbirskiensis* (Averianov & Efimov, 2018: figs 2–4; note that broken surfaces of the anterior–middle caudal centra and anterior caudal neural arches reveal a camellate internal texture), and the following indeterminate specimens – BAS D.8621.25 (Barrett et al., 2026), CPPLIP 0031 (immature individual; Silva Junior et al., 2017), MAU‐Pv‐LI‐601 (Cruzado‐Caballero et al., 2023), MSNM V7157 (Dal Sasso et al., 2016) and MUPA ALG 143 (*contra* Mocho, Perez‐Garcia, et al., 2019; see justification below).

**FIGURE 35:**
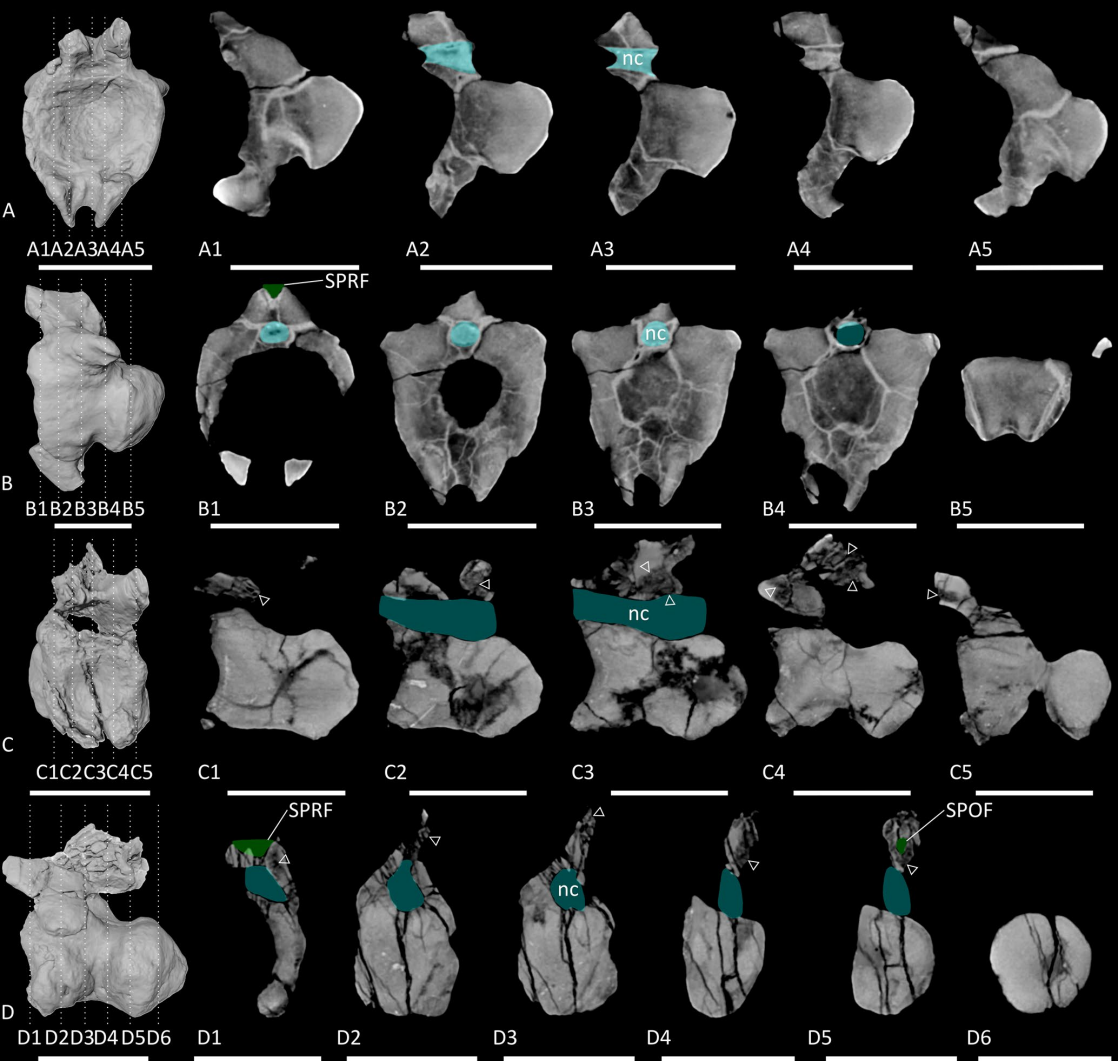
CT scan of indeterminate titanosaur caudal vertebrae. (A–D) 3D rendering (A, C) anterior view, (B, D) left lateral view; (A, B) Titanosauria indet. anterior caudal vertebra MAU‐Pv‐LI‐601, (C, D) Titanosauria indet. middle caudal vertebra MAU‐Pv‐LJ‐472/1; (A1–A5, C1–C5) CT scan slices in parasagittal cross‐section, (B1–B5, D1–D6) CT scan slices in transverse cross‐section. Nc, neural canal; SPRF, spinoprezygapophyseal fossa. Key: Blue highlight, neural canal; green highlight, neural spine fossa; white arrow, pneumatic bone texture. Scale bars = 100 mm.

**FIGURE 36:**
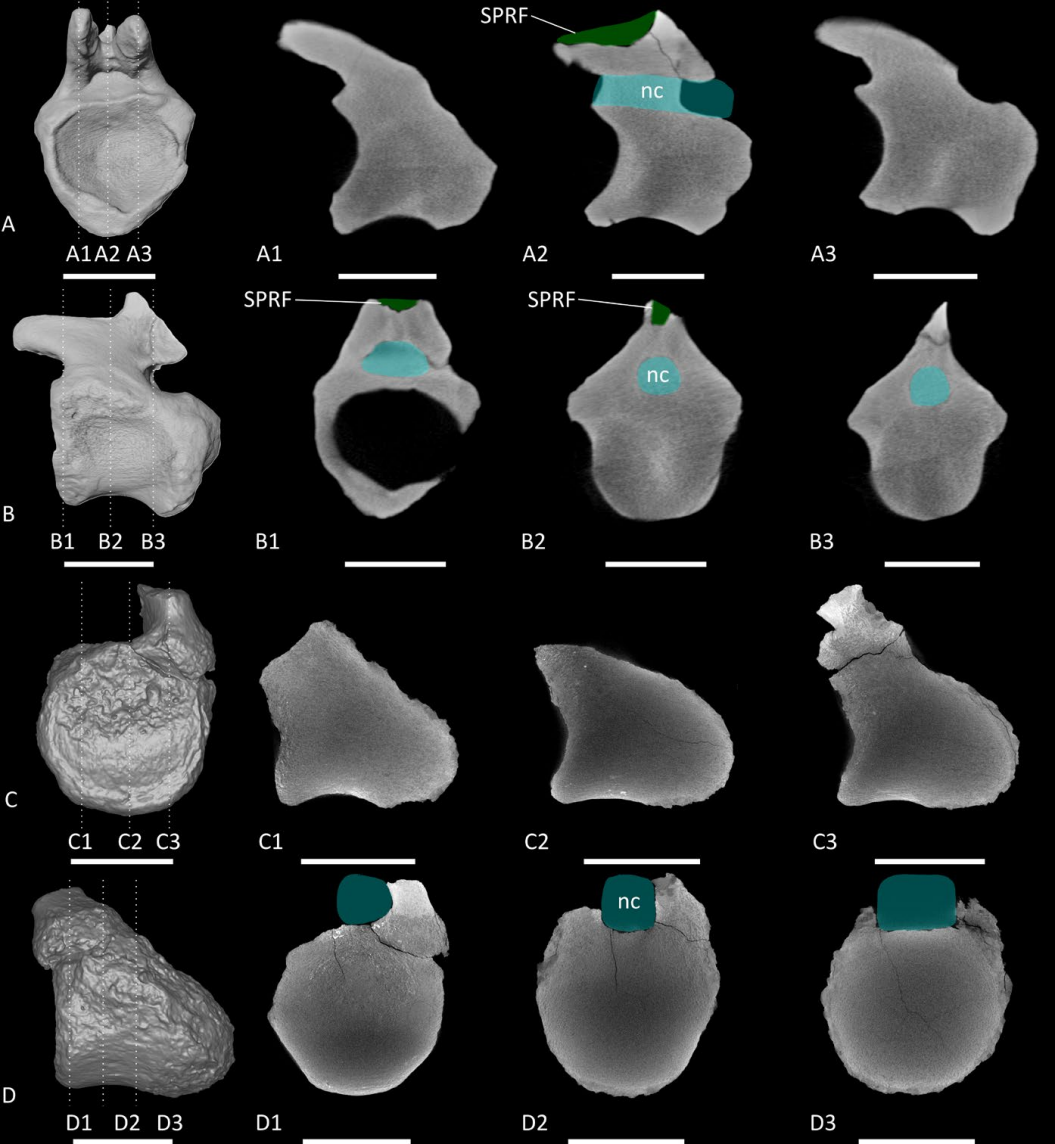
CT scan of indeterminate titanosaur caudal vertebrae. (A, B) 3D rendering, (C, D) surface scan; (A, C) anterior view, (B, D) left lateral view; (A, B) Titanosauria gen. et sp. indet. anterior caudal vertebra MSNM V7157, (C, D) Eutitanosauria gen. et. sp. indet. anterior caudal vertebra BAS D.8621.25; (A1–A3, C1–C3) CT scan slices in parasagittal cross‐section, (B1–B3, D1–D3) CT scan slices in transverse cross‐section. Nc, neural canal; SPRF, spinoprezygapophyseal fossa. Key: Blue highlight, neural canal; green highlight, neural spine fossa. Scale bars = 50 mm.

CT data of indeterminate titanosaurs reveals a dense, apneumatic internal texture in the anterior caudal centra and neural arches of MAU‐Pv‐LI‐601 (Lithostrotia indet.; Cruzado‐Caballero et al., 2023; Figure 35A,B), MSNM V7157 (Lithostrotia indet.; Dal Sasso et al., 2016; Figure 36A,B), MUPA ALG 143 (Lithostrotia indet.; Mocho, Perez‐Garcia, et al., 2019: fig. 11) and BAS D.8621.25 (Eutitanosauria indet.; Barrett et al., 2026; Figure 36C,D); and in the middle caudal centrum of MUPA ALG 144 (Lithostrotia indet.; Mocho, Perez‐Garcia, et al., 2019: fig. 12). This interpretation of MUPA ALG 143–144 differs from the description of camellae in both elements by Mocho, Perez‐Garcia, et al. (2019). The external fossae (= camellae sensu Mocho, Perez‐Garcia, et al., 2019: fig. 11) on the neural arch bases of MUPA ALG 143 are actually shallow external fossae or eroded surfaces, and they do not communicate with internal chambers. The centrum of MUPA ALG 144 possesses areas of less densely packed trabeculae (= camellae sensu Mocho, Perez‐Garcia, et al., 2019: fig. 12) that do not communicate with external fossae. Due to a lack of communication between the supposed camellae with external fossae, these elements cannot be considered pneumatic in origin. The neural arches of a middle caudal vertebra of an indeterminate lithostrotian (MAU‐Pv‐LJ‐472/1; Cruzado‐Caballero et al., 2023; Figure 35C,D) possesses internal pneumatic chambers that communicate with external fossae (i.e. the SPRF and the SPOF). These chambers extend into the zygapophyses and the dorsal tip of the neural spine, but they do not extend into the neural arch bases or centrum.

The lateral surfaces of one anterior caudal centrum of *Ninjatitan zapatai* possess a deep LPF, and the neural arches have deep fossae (Gallina et al., 2021). A LPF occurs on the anterior caudal centra of *Maxakalisaurus topai* (SLB, personal observation of photographs), *Patagotitan mayorum* (Carballido et al., 2017: fig. 2; SLB, personal observation of photographs), *Pellegrinisaurus powelli* (Cerda et al., 2021) and *Rinconsaurus caudamirus* (Ca3 only; broken cross‐section reveals an apneumatic, spongious internal texture; Pérez Moreno et al., 2022). Three posterior caudal centra of *Andesaurus delgadoi* (Ca23–Ca25) possess lateral fossae that are absent in the anterior–middle caudal centra, although the lateral surfaces of the centra of Ca2–Ca7 possess small nutrient foramina (Mannion & Calvo, 2011; SLB, personal observation of photographs). Given the posterior position of Ca23–Ca25, and the absence of such fossae in more anterior elements, it is unlikely that these lateral fossae are pneumatic in origin, and broken surfaces of more anterior elements reveal an apneumatic, spongious internal texture. Some anterior caudal centra of *Xianshanosaurus shijiagouensis* possess nutrient foramina and a small, slit‐like foramen positioned just ventral to the transverse process (Lü et al., 2009; SLB, personal observation of photographs), but whether these features are truly pneumatic in origin is currently unknown.

External evidence for PSP occurs in the anterior caudal neural arches of *Abdarainurus barsboldi* (Averianov & Lopatin, 2020: fig. 11), *Adamantisaurus mezzalirai* (Santucci & Bertini, 2006: plate I), *Baurutitan britoi* (Ca1; Kellner et al., 2005: fig. 11), *Bonatitan reigi* (Zurriaguz, 2025), *Dongyangosaurus sinensis* (Lü et al., 2008), *Dreadnoughtus schrani* (Lacovara et al., 2014), *Dzharatitanis kingi* (Ca1; Averianov & Sues, 2021), *Gandititan cavocaudatus* (Ca1–Ca11; Han et al., 2024: fig. 5), *Hamititan xinjiangensis* (only Ca5 accessible; Wang et al., 2021: fig. 4), *Malawisaurus dixeyi* (anteriormost preserved element only; Gomani, 2005: fig. 14), *Mendozasaurus neguyelap* (Ca1; González Riga et al., 2018: fig. 9), *Tengrisaurus starkovi* (Averianov & Skutschas, 2017: figs 4 and 5; Averianov et al., 2021: fig. 2) and *Yeneen houssayi* (Filippi et al., 2026: fig. 20). These aforementioned taxa possess deep and extensive fossae and/or subfossae on their anterior caudal neural arches, whereas their anterior caudal centra lack external evidence for PSP. The lateral surfaces of some anterior caudal centra of *Adamantisaurus mezzalirai* (Santucci & Bertini, 2006: plate I), *Dreadnoughtus schrani* (Lacovara et al., 2014) and *Gandititan cavocaudatus* (Han et al., 2024) possess prominent nutrient foramina. CT data of one anterior caudal vertebra referred to the lithostrotian titanosaur *Malawisaurus* reveals an apneumatic centrum and a pneumatised neural arch, with small external fossae communicating with internal pneumatic chambers that are separated by thin, compact bone (Wedel, 2009: fig. 2). This internal pneumaticity pattern is comparable to that in the neural arches of Ca1–Ca5 of *Tambatitanis* (Saegusa & Ikeda, 2014).

#### 3.2.6 Saltasaurine titanosaurs

**FIGURE 37:**
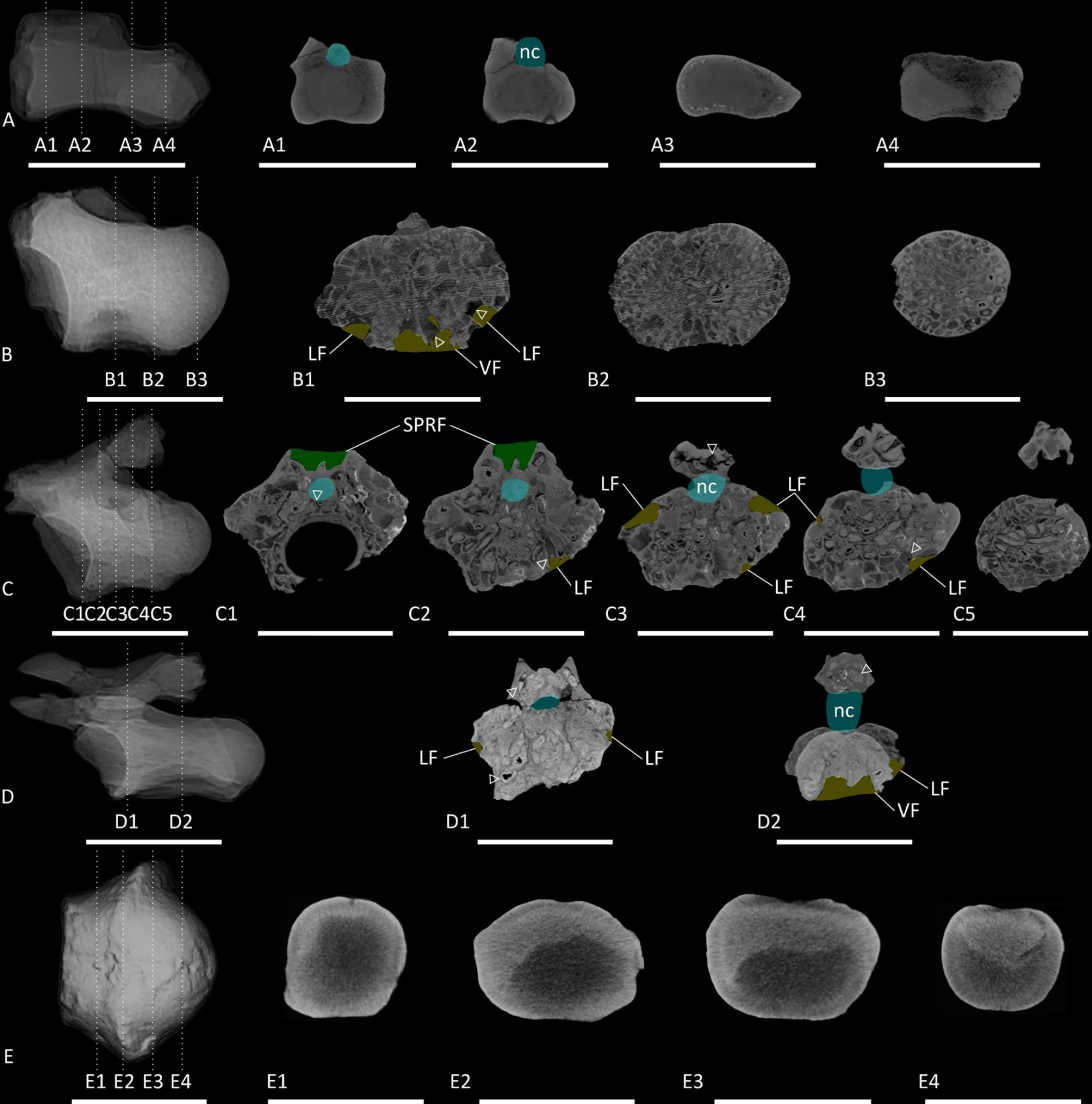
CT scan of saltasaurine sauropod caudal vertebrae. (A–D) CT scan left lateral view, (E) SEM scan dorsal view; (A) *Neuquensaurus australis* middle caudal centrum MCS‐Pv 5/8, (B) *Rocasaurus muniozi* anterior caudal vertebra MPCA‐Pv 47, (C) *Rocasaurus muniozi* middle caudal vertebra MPCA‐Pv 57, (D) *Rocasaurus muniozi* middle caudal vertebra MPCA‐Pv 58, (E) *Yamanasaurus lojaensis* middle caudal vertebra YM‐INPC‐014; (A1–A4, B1–B3, C1–C5, D1, D2, E1–E4) CT scan slices in transverse cross‐section. LF, lateral fossa; nc, neural canal; SPRF, spinoprezygapophyseal fossa; VF, ventral fossa. Key: Blue highlight, neural canal; green highlight, neural spine fossa; yellow highlight, centrum fossa; white arrow, pneumatic bone texture. Scale bars = 100 mm.

**FIGURE 38:**
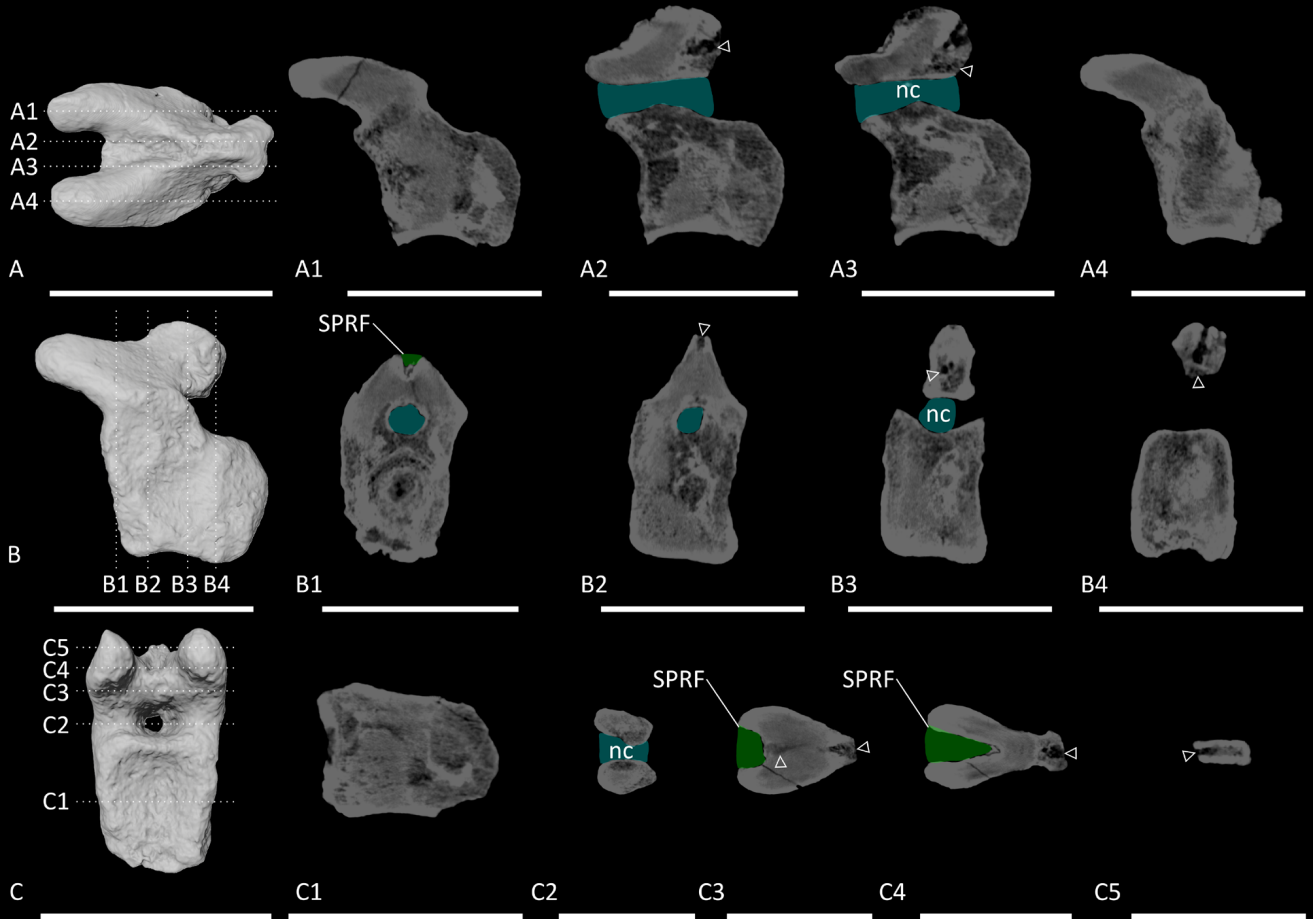
CT scan of *Ibirania parva* middle caudal vertebra MPMA 08–0060‐07. (A–C) 3D rendering (A) dorsal view, (B) left lateral view, (C) anterior view; (A1–A4) CT scan slices in parasagittal cross‐section, (B1–B4) CT scan slices in transverse cross‐section, (C1–C5) CT scan slices in horizontal cross‐section. nc, neural canal; SPRF, spinoprezygapophyseal fossa. Key: Blue highlight, neural canal; green highlight, neural spine fossa; white arrow, pneumatic bone texture. Scale bars = 100 mm.

External and internal evidence for PSP in the caudal vertebrae of saltasaurines has been well‐documented from CT scans, natural breaks and sectioning, with extreme PSP occurring in several taxa (Cerda et al., 2012; Zurriaguz et al., 2017; Zurriaguz & Cerda, 2017). Apesteguía et al. (2020) described pneumatic cavities in the dorsolateral region of a partial middle caudal centrum of *Yamanasaurus lojaensis*. However, CT data of this element reveal a dense, apneumatic internal bone texture (Figure 37E1–E4). Ca1 of *Ibirania parva* possesses a LPF, but the broken cross‐section of this centrum reveals an apneumatic, spongious internal texture (Navarro et al., 2022: fig. 7). CT data of a middle caudal vertebra of *Ibirania* reveals a partially pneumatised neural arch and an apneumatic centrum (MPMA 08‐0060‐07; Figure 38). Although the neural arch of MPMA 08‐0060‐07 is incompletely preserved in the corresponding area in which a SPOF would be located, another middle caudal neural arch of *Ibirania* (LPP‐PV‐0206) preserves the corresponding area, and indeed possesses a SPOF (Navarro et al., 2022: fig. 8). Therefore, we postulate that the pneumatic chambers inside the neural arch of MPMA 08‐0060‐07 communicate externally with the SPRF and the SPOF. These small, separate pneumatic chambers do not extend into the prezygapophyses or the neural arch bases (Figure 38).

*Neuquensaurus australis* possesses small external fossae on the anterior–posterior neural arches (Cerda et al., 2012; Zurriaguz & Cerda, 2017). CT data of one middle caudal centrum and neural arch base shows a dense, apneumatic internal bone texture (MCS‐Pv 5/8; Figure 37A1–A4). From observations of natural breaks and sectioning, another middle caudal centrum and neural arch possesses large, separate internal chambers that communicate with the neural canal and neural arch fossae (Cerda et al., 2012: fig. 1). Another middle caudal vertebra of *Neuquensaurus* possesses two large, separate internal chambers in the centrum, and one large chamber in the neural arch (Zurriaguz & Cerda, 2017: fig. 5). Finally, two posterior caudal centra and neural arches of *Neuquensaurus* each possess several large, separate internal chambers (Cerda et al., 2012: fig. 1; Zurriaguz & Cerda, 2017: fig. 5).

Small external fossae occur on the anterior–posterior caudal centra and neural arches of *Rocasaurus muniozi* (note that the immature individual represented by the holotype does not preserve anterior caudal vertebrae; Cerda et al., 2012; Garcia & Salgado, 2013; Zurriaguz & Cerda, 2017; SLB, personal observation of photographs). From CT data of anterior (MPCA‐Pv 47) and middle (MPCA‐Pv 57–58) caudal vertebrae, these external fossae communicate with extensive internal pneumatic camellate chambers that occupy each element entirely (Figure 37B–D). A broken cross‐section of a posterior caudal centrum possesses several large, separate internal chambers (Cerda et al., 2012: fig. 1), whereas another posterior caudal centrum possesses a single large chamber that communicates externally with the ventral fossa (Zurriaguz & Cerda, 2017: fig. 5).

Approximately the 25 anteriormost caudal centra and neural arches of *Saltasaurus loricatus* possess small external fossae (Cerda et al., 2012; Wedel & Taylor, 2013; Zurriaguz & Cerda, 2017). Broken cross‐sections reveal that middle caudal centra and neural arches possess pneumatic camellate chambers that communicate with the small external fossae (Cerda et al., 2012: fig. 1; Zurriaguz & Cerda, 2017: fig. 4). Several posterior caudal centra each possess a singular, large chamber, and in at least one element, this chamber communicates with the floor of the neural canal (Cerda et al., 2012: fig. 1; Zurriaguz & Cerda, 2017: fig. 5). However, one posterior caudal neural arch possesses a dense, apneumatic internal texture (Zurriaguz & Cerda, 2017: fig. 5). Finally, the anterior–posterior caudal centra and neural arches of an indeterminate saltasaurine possess small external fossae (MACN‐Pv RN 233; Zurriaguz et al., 2017). CT data of the posterior caudal vertebra of this specimen (MACN Pv 233‐5; Zurriaguz et al., 2017: fig. 15) reveals that the external fossae communicate with pneumatic chambers inside the centrum and neural arch.

In general, the small‐sized fossae on the centra and neural arches of saltasaurine caudal vertebrae communicate internally with small interconnected camellate chambers, as well as large camerate chambers, depending on the element. However, there are elements that lack external fossae, yet possess large internal camerate chambers that appear to have expanded into the centrum and/or neural arches via the neural canal.

## 4 DISCUSSION

### 4.1 Phylogenetic and serial distribution of postcranial skeletal pneumaticity in sauropodomorph dinosaur caudal vertebrae

From our review of evidence for PSP in sauropodomorph caudal vertebrae, lateral fossae (i.e. fossae that do not possess defined rims) occur in several non‐neosauropod gravisaurian lineages, including at least one non‐eusauropod, as well as in Mamenchisauridae and Turiasauria (Figure 21). We do not regard a lateral fossa as evidence for PSP. A true lateral pneumatic fossa evolved in diplodocoid neosauropods, with this feature lost in dicraeosaurids. Within Macronaria, a true LPF appears to have evolved (and/or been lost) a number of times, given its presence in some (but not most) brachiosaurids, at least one non‐titanosaurian somphospondylan, and several titanosaurian lineages, including some early diverging members, as well as within Aeolosaurini, Lognkosauria, Rinconsauria and Saltasaurinae. External evidence for PSP in the caudal neural arches (i.e. subfossae within fossae, accessory fossae and surfaces heavily excavated by fossae) is absent in all non‐neosauropods, except for one mamenchisaurid (*Mamenchisaurus sanjiangensis*). Within Diplodocoidea, no evidence of caudal vertebral PSP has been identified in the neural arches of early diverging members, dicraeosaurids and apatosaurine diplodocids, whereas it has been identified in some rebbachisaurids and several diplodocine diplodocids. All non‐titanosaurian macronarian caudal neural arches lack external evidence for PSP, except for one euhelopodid somphospondylan (*Tambatitanis*). Within Titanosauria, external evidence for caudal neural arch PSP occurs in several early‐diverging members, Lognkosauria and Saltasauroidea, including Saltasaurinae. Except for *Ninjatitan* and Saltasaurinae, titanosaurs that possess external evidence for caudal neural arch PSP lack such evidence in their centra.

Based upon our review, confirmation of caudal vertebral PSP via direct observation of communication between external fossae and internal chambers does not always align with that predicted from external evidence alone (compare Figures 21 and 22). The anterior caudal centra of the non‐neosauropod eusauropod ‘*Bothriospondylus madagascariensis*’, the rinconsaurian titanosaur *Rinconsaurus* and the saltasaurine *Ibirania* each possess a lateral fossa/or a true LPF, but lack a pneumatic centrum. The distal‐middle–posterior caudal neural arches of the diplodocine *Tornieria*, the anterior–middle caudal neural arches of the brachiosaurid *Giraffatitan*, the anterior caudal neural arches of the diamantinasaurian somphospondylans *Savannasaurus* and *Wintonotitan*, as well as the anterior–middle caudal centra of the lithostrotian titanosaur *Volgatitan*, each lack external evidence for PSP; however, internal evidence confirms its presence. Some saltasaurine caudal centra and neural arches lack external evidence for PSP in elements that are pneumatic, and the external fossae that do communicate with internal chambers often lack defined rims, are not subdivided, nor do they possess foramina. Given that such external features are typically used as criteria for assessing the internal pneumaticity of an element (Britt, 1993; Wedel et al., 2000; O'Connor, 2006), this further complicates the assessment of internal pneumatic features of an element based purely on external anatomy. There are currently no occurrences of sauropodomorph caudal neural arches possessing external evidence for PSP but lacking internal evidence for PSP. However, more CT scan data in elements with such external features are needed to determine if this observation holds true more broadly. We suggest that the presence of a lateral fossa or a true LPF in a caudal centrum should not be equated with the presence of internal pneumaticity, unless there is direct data on internal features that support such an interpretation.

Our survey of the external and internal indicators of PSP in sauropodomorph dinosaur caudal vertebrae reveals a greater diversity and plasticity both inter‐ and intra‐specifically than previously recognised. Furthermore, acquisitions and losses of caudal vertebral PSP are numerous and widespread across several clades. Given this, it is difficult to draw general conclusions for the pneumatic features of higher‐level phylogenetic clades because there is often such disparity within a given lineage (Figures 21 and 22).

We find no evidence for pneumatic caudal vertebrae in sauropodomorph taxa with apneumatic presacral vertebrae. Apneumatic caudal vertebrae also occur in many taxa that possess presacral PSP. It therefore appears that presacral PSP is a precursor (and perhaps therefore a prerequisite) for caudal vertebral PSP in sauropodomorph dinosaurs.

Combining the currently known distribution of external and internal evidence for caudal vertebral pneumaticity, we observe a recurring pattern in which PSP invades the anterior caudal vertebrae across several neosauropod lineages, with evidence for at least five independent acquisitions and/or reversals. We hypothesise an acquisition of caudal vertebral PSP in each of Diplodocidae and Rebbachisauridae; one acquisition in Titanosauriformes, but whether this occurred independently in each of Brachiosauridae and Somphospondyli is not currently known; at least one loss in Lithostrotia and/or Eutitanosauria; and one acquisition in Saltasauridae.

At least three independent acquisitions of extreme caudal vertebral PSP (i.e. pneumatisation of middle and/or posterior caudal vertebrae, in addition to the anterior ones) can be recognised within Neosauropoda in Diplodocinae (i.e. *Tornieria*; anterior–posterior caudal vertebrae), Brachiosauridae (i.e. *Giraffatitan*; at least anterior–middle caudal vertebrae) and Saltasaurinae (i.e. *Neuquensaurus*, *Rocasaurus* and *Saltasaurus*; anterior–posterior caudal vertebrae) (Figure 22). Diplodocine taxa known from relatively complete tails (i.e. *Barosaurus*, *Diplodocus* and *Tornieria*) possess the most invasive external pneumatic fossae on the lateral and ventral surfaces of the anterior–middle caudal centra and neural arches. The brachiosaurid *Giraffatitan* only shallowly pneumatises the lateral and ventral surfaces of its anterior–middle caudal centra. Similarly, the pneumatic fossae on the lateral and ventral surfaces of the anterior–posterior caudal centra and neural arches of saltasaurine taxa are much smaller and less invasive than those of diplodocines. This new observation reveals that saltasaurines are not the only sauropod taxa to possess pneumatic posterior caudal vertebrae. No middle caudal vertebrae of rebbachisaurids have been imaged internally, so it is possible that a fourth acquisition of extreme caudal vertebral PSP occurred in this group, given the extensive external fossae from which foramina invade the anterior caudal neural spines of some taxa (e.g. Figures 26, 27, 28). However, the middle caudal vertebrae of rebbachisaurids do not possess the extensive external fossae of their anterior counterparts (e.g. Mannion et al., 2019 and Figure 27M–R). Furthermore, the broken cross‐section of a middle caudal centrum of *Nigersaurus* (MNHN.F.GDF2008) reveals an apneumatic, spongious internal texture (Figure 25L).

We urge caution when drawing macroevolutionary conclusions about sauropod caudal vertebral PSP solely from external indicators, because their presence is not necessarily indicative of PSP (see above). Some theropods (including extant Aves) and crocodylians possess deep lateral fossae on vertebrae spanning the axial skeleton that do not invade the centrum, and vascular foramina can look identical to pneumatic foramina (Atterholt & Wedel, 2023; Aureliano, Almeida, et al., 2026; O'Connor, 2004; O'Connor, 2006; Watanabe et al., 2015; Wedel & Taylor, 2013). Furthermore, PSP in the vertebrae of extant Aves is highly variable, with independent acquisitions of an expansion and a reduction of a common pattern of pneumatised elements prevalent across clades (Gutherz & O'Connor, 2021; Gutherz & O'Connor, 2022; O'Connor, 2006; O'Connor, 2009; Smith, 2012). Although seemingly rare in terms of numbers of species with external evidence for PSP, independent acquisitions of caudal vertebral pneumaticity in non‐avialan theropod dinosaurs have also been recognised in carcharodontosaurids, megalosaurids, megaraptorans, oviraptorosaurians and therizinosauroids (Aranciaga Rolando et al., 2020; Benson et al., 2012; Kotevski et al., 2025). Given this, it is not unreasonable to infer that sauropods possessed a similar level of plasticity in the distribution of caudal vertebral PSP.

The internal bone texture of the caudal vertebrae in each of the CT scanned sauropodomorph taxa is highly variable, and, in some instances, it is difficult to ascertain which pneumaticity category (Table 1) should be applied (e.g. camerate, camellate). Furthermore, there is variation in how and/or where an element is pneumatised: pneumatisation can occur via lateral fossae, ventral fossae, neural canal fossae (roof and floor), neural arch and spine fossae; and a vertebral centrum can be pneumatised via the neural arch, and vice versa. Pneumatic internal bone texture also differs between elements within a taxon: for example, it varies along the caudal vertebral series of *Tornieria*. External pneumatic fossae can be small and/or large, and there does not appear to be a distinct pattern that can be followed to estimate the internal pneumatic features of an element, either intra‐specifically (i.e. communication with all or only some external fossae) or interspecifically (i.e. phylogenetic position of a taxon). Therefore, we urge caution in utilising ‘novel’ external pneumatic features in the diagnosis of a species (e.g. Cerda et al., 2021; Fanti et al., 2013; Filippi et al., 2026; Gallina et al., 2014; Navarro et al., 2022; Novas et al., 2019; Wilson, 2002) because of their high intraspecific plasticity. Furthermore, the intraspecific plasticity in internal bone texture complicates the characterisation of pneumatic features (e.g. camerate versus camellate internal texture) in phylogenetic analyses (e.g. some middle caudal vertebrae of *Neuquensaurus* are apneumatic, whereas others are pneumatic). Such intraspecific variability in the development of caudal vertebra PSP has also been observed in accipitriform birds (Gutherz & O'Connor, 2022).

The posterior expansion of PSP along the caudal vertebral column has been described as following a ‘neural arch‐first’ pattern in theropods (Benson et al., 2012) and rebbachisaurids (Fanti et al., 2013). Whereas we find some evidence for a ‘neural arch‐first’ pattern in somphospondylans (Figures 21 and 22), the distribution of pneumatisation across the sauropod evolutionary tree does not appear to follow a distinct pattern. External features indicate that some eusauropods might possess PSP in the anterior caudal centra, and the phylogenetically earliest external evidence for PSP in the caudal neural arches occurs in a species of mamenchisaurid. Evidence from internal imaging does not resolve the ‘centrum‐first’ or ‘neural arch‐first’ pattern in sauropods, and therefore, further work is needed.

### 4.2 Is postcranial skeletal pneumaticity in sauropodomorph dinosaur caudal vertebrae correlated with mass distribution and body size?

It has been hypothesised that PSP might have facilitated the evolution of gigantism by lightening the skeleton (Britt, 1997; Witmer, 1997, Wedel, 2009; Sander, 2013). Under this scenario, once established, the reduction in skeletal mass and body density would have saved energy during locomotion and foraging, benefiting sauropods of all sizes (Benson et al., 2012; Sander, 2013). Alternatively, it has been suggested that PSP did not initially evolve as a way of lightening the skeleton, but was secondarily exapted for that purpose (Wedel, 2006, 2007).

The centre of mass in sauropods is located between the pectoral and pelvic girdles (Alexander, 1985). Any anterior or posterior deviation from this centre of mass would be due to differences in the weight and/or relative proportions of the cantilevered neck and tail. Theoretically, an expansion of PSP into the neck or tail would affect this centre of mass due to changes in relative weights, shifting it anteriorly or posteriorly to keep the cantilever level. Among their sampled taxa, Bates et al. (2016: fig. 4) recorded the most anterior shift in centre of mass for sauropods in *Mamenchisaurus*. Within Mamenchisauridae, two taxa possess external evidence for PSP in the anterior caudal centra (*Bellusaurus* and *Omeisaurus tianfuensis*), one taxon possesses external evidence for PSP in the anterior caudal neural arches (*Mamenchisaurus sanjianensis*), and CT data of another taxon shows an absence of internal evidence for caudal vertebral PSP (*Wamweracaudia*). Among taxa with extreme caudal vertebral PSP (i.e. diplodocine diplodocoids, the brachiosaurid titanosauriform *Giraffatitan* and saltasaurine titanosaurs), the diplodocine *Barosaurus* and *Giraffatitan* have a more anterior shift in their centre of mass than that of the diplodocine *Diplodocus* and the saltasaurine *Neuquensaurus* (Bates et al., 2016: fig. 4). Furthermore, the eusauropod *Cetiosaurus* and the dicraeosaurine diplodocoid *Dicraeosaurus*, which possess apneumatic tails, have a centre of mass similar to that of *Diplodocus* and *Neuquensaurus* (Bates et al., 2016: fig. 4). Therefore, the expansion of PSP into sauropod caudal vertebrae does not appear to correspond with a change in centre of mass. We postulate that the anterior shift in centre of mass for *Mamenchisaurus* is likely due to the extreme neck length of mamenchisaurids (Christian et al., 2013), and not a consequence of caudal vertebral PSP.

Qualitatively, caudal vertebral PSP in sauropods also does not appear to correspond with body mass: the small‐bodied *Dicraeosaurus* (10.2 tonnes; Benson et al., 2014) lacks evidence for caudal vertebral PSP, whereas *Saltasaurus* (5.8 tonnes; Benson et al., 2014) possesses extreme caudal PSP (see above). Conversely, *Tornieria* (12.3 tonnes; Benson et al., 2014) and *Giraffatitan* (34 tonnes; Benson et al., 2014) both possess extreme caudal PSP. Among the largest sauropods, the lognkosaurian titanosaurs (Carballido et al., 2017), some taxa lack external evidence for PSP in the caudal vertebrae (e.g. *Futalognkosaurus*, *Notocolossus*, *Quetecsaurus*), whereas others possess external evidence for PSP in the anterior caudal centra (*Patagotitan*) and anterior caudal neural arches (*Mendozasaurus*). Furthermore, whereas Diplodocidae is characterised by an elongated whiplash tail with ~80 vertebrae, saltasaurids are characterised by a short tail with ~35 vertebrae (Wilson, 2005). This implies that an increase in tail length alone is also not a driver of increased caudal vertebral PSP.

In non‐avialan theropod dinosaurs, taxa with external evidence for caudal vertebral PSP range from small‐ (26 kg) to large‐bodied (2994 kg) species (Benson et al., 2012). Extreme caudal vertebral PSP also occurs across species with a wide range of body masses, with two orders of magnitude separating the oviraptorosaurians *Khaan mckennai* and *Gigantoraptor erlianensis* (Benson et al., 2014). Therefore, as we find for sauropods, caudal vertebral PSP in theropods does not appear to correspond with an increase (or decrease) in body mass. Buchmann et al. (2019: pp. 3) stated that ‘caudal vertebrae are normally not pneumatised in pterosaurs’; however, we are unable to find any evidence of pterosaur caudal vertebrae possessing either external or internal evidence for PSP.

Pneumatic caudal vertebrae have been recognised in several groups of extant Aves (e.g. Apostolaki et al., 2015; Gutherz & O'Connor, 2022; O'Connor, 2004). In flightless members, pneumatic foramina occur on the caudal vertebrae of large‐bodied species of Struthioniformes (ostrich) and Rheiformes (rhea), but are absent in Casuariiformes (cassowary) and Dromaiformes (emu) (Apostolaki et al., 2015). Gutherz and O'Connor (2022) found that small‐ and large‐bodied members of Accipitriformes possess pneumatic caudal vertebrae, and that while intra‐ and interspecifically variable, caudal vertebral pneumaticity exhibits some phylogenetic signal. Those authors concluded that such variation could be evidence of stochastic development. Such initially neutral evolution (Witmer, 1997) was also hypothesised by Wedel and Taylor (2013) as occurring in the pneumatic caudal vertebrae of *Giraffatitan* and *Apatosaurus*, which negligibly reduced the weight of the tail. Our results lend support to the hypothesis that PSP did not initially evolve as a way of lightening the skeleton (Wedel, 2006, 2007), and we further hypothesise that it would not have been an advantageous function. Instead, the extension of PSP into the caudal vertebrae of several sauropod clades evolved multiple times as a result of the opportunistic nature of pneumatic diverticula (Witmer, 1997; Taylor & Wedel, 2013). This would serve to explain why both small‐ and large‐bodied sauropods, including those with relatively short and long tails, evolved (and lost) caudal vertebral PSP.

Whereas we qualitatively suggest that the development of caudal vertebral PSP in sauropods does not correspond with changes in body shape and mass, this requires quantitative assessment. The lack of an obvious signal between caudal vertebral PSP and a particular body size/shape parameter could instead be evidence for multiple overlapping signals that represent complex selective pressures favouring the expansion (and possibly also the loss) of PSP into the tail. To test this, further research is needed to better understand the physiological effects of PSP development in the caudal vertebrae of sauropod dinosaurs, such as tail mass, centre of mass and an increased requirement to shed excess heat (e.g. Sander, 2013). Furthermore, what drives the development and expansion of PSP in extant taxa needs to be better understood so that we can test for such evidence in extinct taxa.

## 5 CONCLUSIONS

Within Sauropodomorpha, we find evidence for at least five independent acquisitions and/or reversals of postcranial skeletal pneumaticity (PSP) invading the anterior caudal vertebrae in several neosauropod lineages. Extreme caudal vertebral PSP (i.e. the pneumatisation of middle and/or posterior caudal vertebrae, in addition to the anterior caudal vertebrae) is demonstrated for the diplodocine diplodocoid *Tornieria africana*, the brachiosaurid titanosauriform *Giraffatitan brancai* and several saltasaurine titanosaurs. Internal imaging reveals a high diversity in the pneumatic internal bone texture of sauropod caudal vertebrae both inter‐ and intra‐specifically, indicating that sauropod caudal vertebral PSP is more plastic than previously recognised. We find that both small‐ and large‐bodied sauropods, including those with relatively short and long tails, possess evidence for caudal vertebral PSP. Therefore, there does not currently appear to be a relationship between body size or shape with caudal vertebral pneumaticity. However, internal imaging of the largest sauropods—the lognkosaurian titanosaurs—is needed to better understand this. Future research should also expand taxonomic sampling of CT scanned specimens because current observations on the distribution of external indicators of PSP do not closely align with those confirmed from internal imaging.

## AUTHOR CONTRIBUTIONS

SLB conceived, designed and performed the experiments, analysed and interpreted the data, produced figures and tables, and led the writing of the manuscript. DS facilitated CT scanning of specimens, analysed and interpreted the data, contributed to drafts of the manuscript and approved the final manuscript. PU interpreted the data, contributed to drafts of the manuscript and approved the final manuscript. SFP interpreted the data, contributed to drafts of the manuscript and approved the final manuscript. PA conducted CT scanning, analysed the data and approved the final manuscript. PDM contributed to the project design, interpreted the data, made critical revisions of the manuscript and approved the final manuscript.

## Supporting information


Data S1.

## Data Availability

The data that supports the findings of this study are available in the supplementary material of this article.
